# Randomized, Controlled, Thorough QT/QTc Study Shows Absence of QT Prolongation with Luseogliflozin in Healthy Japanese Subjects

**DOI:** 10.1371/journal.pone.0139873

**Published:** 2015-10-07

**Authors:** Yuji Kumagai, Tomoko Hasunuma, Soichi Sakai, Hidekazu Ochiai, Yoshishige Samukawa

**Affiliations:** 1 School of Medicine, Kitasato University, Kanagawa, Japan; 2 Division of Rheumatology, Department of Internal Medicine, School of Medicine, Toho University, Tokyo, Japan; 3 Taisho Pharmaceutical Co., Ltd., Tokyo, Japan; University Heart Center, GERMANY

## Abstract

Luseogliflozin is a selective sodium glucose co-transporter 2 (SGLT2) inhibitor. To evaluate the cardiac safety of luseogliflozin, a thorough QT/QTc study was conducted in healthy Japanese subjects. The effects of moxifloxacin on QT prolongation in Japanese subjects were also evaluated. In this double-blind, placebo- and open-label positive-controlled, 4-way crossover study, 28 male and 28 female subjects received a single dose of luseogliflozin 5 mg (therapeutic dose), luseogliflozin 20 mg (supratherapeutic dose), placebo, and moxifloxacin 400 mg. Serial triplicate digital 12-lead electrocardiograms (ECGs) were recorded before and after dosing, and results were analyzed using the Fridericia correction (QTcF) method. Serial blood sampling was performed for pharmacokinetic analyses of luseogliflozin and moxifloxacin to analyze the relationship between QTcF interval and plasma concentration. The upper limits of the two-sided 90% confidence intervals (CIs) for baseline and placebo-adjusted QTcF intervals (ΔΔQTcF) in the 5 mg and 20 mg luseogliflozin groups were less than 10 ms at all time points. No correlation between plasma luseogliflozin concentrations and ΔΔQTcF was observed. In the moxifloxacin group, the lower limits of the two-sided 90% CIs for ΔΔQTcF were greater than 5 ms at all time points. A positive relationship was observed between plasma moxifloxacin concentration and change in ΔΔQTcF. Luseogliflozin was well tolerated at both dose levels. The majority of adverse events were mild in severity, and no serious or life-threatening adverse events occurred. Neither therapeutic (5 mg) nor supratherapeutic (20 mg) doses of luseogliflozin affected QT prolongation in healthy Japanese subjects.

## Introduction

Luseogliflozin, a novel 1-thio-D-glucitol derivative, is a highly selective sodium glucose cotransporter 2 (SGLT2) inhibitor that is approved for marketing in Japan for the treatment of type 2 diabetes [1]. Luseogliflozin specifically inhibits the activity of SGLT2 in the renal proximal tubule and shows hypoglycemic effects based on promotion of urinary glucose excretion in various animal models [2]. The action of luseogliflozin is postulated to normalize hyperglycemia by stimulating excretion of glucose from blood into urine via inhibition of the reabsorption of filtered glucose in the renal proximal tubule. Therefore, the pharmacological effects of SGLT2 inhibitors are insulin independent, making them an attractive new therapeutic target in type 2 diabetes mellitus [3]. Several SGLT2 inhibitors have already appeared on the market, and others are under the development. Safety and efficacy have been demonstrated in most [4].

In clinical studies, a single dose of luseogliflozin (1–25 mg) was rapidly absorbed (time to maximum plasma concentration [T_max_] ≤ 2.25 hours), maximum plasma concentration (C_max_) and area under the plasma concentration–time curve from time zero to infinity (AUC_inf_) were dose proportional, and half-life (T_1/2_) ranged from 9.2 to 14 h. None of the major pharmacokinetic (PK) parameters (C_max_, AUC_inf_, T_max_, and T_1/2_) were affected by feeding. Additionally, PK parameters were not affected after multiple doses of luseogliflozin (5 or 10 mg/day), indicating no clinically significant accumulation. In these studies, no serious adverse events were observed. All adverse drug reactions were transient and mild in severity in healthy subjects [5]. In Japanese patients with type 2 diabetes mellitus, PK parameters were similar to those in healthy subjects. Pharmacodynamic activity as measured by urinary glucose excretion was significantly increased in all luseogliflozin treatment groups (0.5, 1, 2.5, and 5 mg/day) in comparison to placebo, and cumulative urinary glucose excretion for the 24-h period after dosing was dose dependent. Additionally, significant hypoglycemic activity persisted from dosing before breakfast until after supper on Day 7 [6].

In non-clinical studies, effects of luseogliflozin on the cardiovascular system were investigated via the human ether-a-go-go-related gene (hERG) current in hERG-transfected human embryonic kidney cells (HEK293 cells) *in vitro*; action potential in the papillary muscle of anesthetized guinea pigs; and blood pressure, heart rate, and electrocardiogram (ECG) in conscious beagle dogs. A slight suppression of hERG current (7.7%) *in vitro* was observed at 9.6 μmol/L, a concentration that was approximately 370 times greater than the maximum observed C_max_ of a 5 mg dose in diabetic subjects in a clinical study. No effect on the cardiovascular system was observed in guinea pigs or dogs.

Establishing the cardiac safety of new drugs is critically important, particularly for drugs used to treat diabetes, because patients with type 2 diabetes have increased risk of cardiovascular disease [7], and several antidiabetic agents have the potential for QT prolongation. [8] To evaluate the cardiac safety of therapeutic (5 mg) and supratherapeutic (20 mg) doses of luseogliflozin, a thorough QT/QTc study based on the International Conference of Harmonization (ICH) E14 guidelines [9] was conducted in healthy Japanese subjects at a single clinical site in Japan. The effects of moxifloxacin, a positive control, on QT prolongation in Japanese subjects were also evaluated.

## Methods

### Study design

This study employed a randomized, single dose, double-blind, placebo- and open-label positive-controlled (moxifloxacin), 4-way crossover study based on a Williams design consisting of 4 treatment sequences to reduce carry-over effects. The study was conducted at Kitasato Institute Hospital (Tokyo, Japan) from July 6^th^ 2011 to September 15^th^ 2011. Eligible subjects received a single dose of luseogliflozin 5 mg, luseogliflozin 20 mg, placebo, and moxifloxacin 400 mg (positive control; Avelox^®^ tablets 400mg; manufactured by Bayer Yakuhin, Ltd.) with a minimum 7-day interim washout period after each dose period. All treatments were administered with 200 mL of water after at least 10-hour overnight fast, and no food was allowed until 4 hours after dosing. All treatments were administered in a randomized sequence. The random assignment manager randomly assigned treatment groups using the Williams Design to determine the order of treatments within each treatment sequence by the time of study drug administration to the first admission group. A randomization code was created for each treatment sequence sealed and securely retained until unblinding and were provided to the sponsor after unblinding.

Prior to the initial review by the IRB, amendments were made in the protocol including changes in the volume of blood and number of blood sampling for pharmacokinetics and changes in wordings for clarification; the protocol was revised from Version 1 to Version 2. There were no other changes in the protocol.

### Trial registration

This study is registered at JAPIC Clinical Trial Information. Trial registration number: JapicCTI–132352. The authors confirm that all ongoing and related trials for this drug/intervention are registered. The trial was registered post patient enrollment. This was due to the assumption the authors followed, which was a joint statement by JPMA (the Japanese Pharmaceutical Manufacturers Association), EFPIA, and PhRMA’s that requires that all clinical trials conducted in patients should be registered, the statement did not extend to a healthy normal subject population in whom the current study was conducted.

### Subjects

We included healthy Japanese males and females between the ages of 20 and 44 y, with body mass indices (BMIs) between 18.0 and 24.9 kg/m^2^. Exclusion criteria were QT interval corrected for heart rate using Fridericia’s formula (QTcF) at screening or prior to first study drug administration >450 ms; congenital disorder or cardiac disorder, or history thereof; risk factor for or history of torsade de pointes (TdP), e.g., heart failure, hypokalemia, or a family history of long QT syndrome; history of attack of unconsciousness possibly associated with TdP; unsuitable 12-lead ECG waveform for the evaluation of QT/QTc interval prolongation at screening and prior to study drug administration, e.g., drift, myogram, T wave morphology, remarkable sinus arrhythmia, or frequent extrasystoles; and if female, pregnancy, lactation, possible pregnancy, intention to become pregnant during the course of the study, or positive pregnancy test at screening or prior to study drug administration. No concomitant therapy including over-the-counter medications was allowed throughout the study, except lacrimal fluid (eye lotion) for contact lenses.

Every subject provided his/her own written informed consent prior to participation in the study. The protocol was approved by the local institutional review board. This study was conducted in compliance with the Good Clinical Practice, ICH E14 guidelines, and the Declaration of Helsinki. This study is registered at JAPIC Clinical Trial Information as record number JapicCTI–132352.

The sample size was selected in reference to thorough QT/QTc studies carried out in other populations with a crossover design [10] similarly to the present study as the results of thorough QT/QTc studies in Japanese subjects have not been made public to date.

### Electrocardiogram assessment

Serial triplicate digital 12-lead electrocardiograms (ECGs) were recorded over 5 min in the supine position after resting for 10 min for each time point (pre-dose [50–70 min before dosing], 0.5, 1, 2, 3, 4, 6, 8, and 24 h post-dose) using an electrocardiograph (ELI250, Mortara). The same model of 12-lead digital surface ECG monitor was employed for all subjects at all time points. The same lead was used throughout the study. The interval between the start of each recording was ≥1 min, and ECG was recorded for 10 s for each chart. All 12-lead ECG charts for QT/QTc analysis were sent electronically to the central ECG analysis facility. RR and QT intervals were measured in accordance with the procedures of the facility. The time of recording and subject identification information were blinded before measurement. QTc intervals were corrected by Fridericia’s (QTcF = QT/RR^1/3^) and Bazett’s (QTcB = QT/RR^1/2^) formulae. QTcF was the primary endpoint, and QTcB was the secondary endpoint.

### Pharmacokinetic evaluation

Plasma concentrations of unchanged luseogliflozin and its metabolites (M2, O-deethyl form and M17, carboxylic acid form formed by hydroxylation at the hydroxyl group of the terminal ethyl group) and plasma concentrations of unchanged moxifloxacin were determined by validated methods using high-performance liquid chromatography tandem mass spectrometry (LC-MS/MS). Blood samples for pharmacokinetic measurements were collected at pre-dose and 0.5, 1, 2, 3, 4, 6, 8, 12, 24, and 48 h post-dose in each study period. If blood collection coincided with 12-lead ECG recording, blood was collected after ECG recording. To measure unchanged luseogliflozin and its metabolites or moxifloxacin, 5 mL or 7mL blood samples were collected into tubes containing sodium heparin, respectively. Whole blood was centrifuged immediately after collection at the site (4°C, 3000 rpm, 15 min) to obtain plasma samples, which were stored at -70°C until analysis. The plasma concentrations of luseogliflozin and its metabolites were determined by two separate methods at JCL Bioassay Corp. (Hyogo, Japan). Luseogliflozin, its metabolites and its deuterium-labeled internal standard (luseogliflozin-d5, M2-d5 and M17-d5, Taisho Pharmaceutical Co. Ltd., Japan) were extracted from plasma by solid phase extraction. Extracts were analysed by LC-MS/MS. The lower limit of quantification (LLOQ) for luseogliflozin and its metabolites (M2 and M17) in plasma were 0.05 and 0.1 ng/mL, respectively. The plasma concentrations of moxifloxacin in plasma were determined at Taisho Pharmaceutical Co. Ltd. (Saitama, Japan). Moxifloxacin and internal standard (moxifloxacin-d4, Toronto Research Chemicals Inc., Canada) were extracted from plasma by protein precipitation. Extracts were analysed by LC-MS/MS. The LLOQ for moxifloxacin was 0.1 μg/mL. The pharmacokinetic parameters, including C_max_, T_max_, and AUC_inf_, were derived by noncompartmental analysis based on the plasma concentrations of unchanged luseogliflozin and its metabolites (M2 and M17) and unchanged moxifloxacin. Summary statistics were provided for the pharmacokinetic parameters by treatment group.

### Statistical analysis

Treatment comparisons between luseogliflozin therapeutic dose or supratherapeutic dose and placebo (ΔΔQTc) were analyzed using a linear mixed model with treatment, sequence, period, scheduled time, and the interaction of treatment with scheduled time as fixed effects, subject as a random effect, and baseline QTc as a covariate. The effect of luseogliflozin on QT/QTc interval prolongation was regarded as negative when the upper bound of the 90% two-sided confidence interval (CI) for the mean effect on the QT/QTc interval exceeded 10 ms at all time points in the luseogliflozin groups [8].

A categorical analysis was also performed. New onset of QTc greater than 450, 480, or 500 ms after dosing or change from baseline in QTc greater than 30 or 60 ms was categorized in each treatment group. Scatter plots of ΔΔQTcF and plasma concentrations were prepared for luseogliflozin groups and the moxifloxacin group to investigate the relationship between ΔΔQTcF and plasma concentrations.

### Safety

Safety assessments included recording of adverse events, vital signs (body temperature, blood pressure, and pulse rate), physical examinations, ECGs, and clinical laboratory tests. Vital signs were recorded at pre-dose and 0.5, 1, 2, 3, 4, 24, and 48 h post-dose in each study period. ECGs for safety analysis were recorded at pre-dose and 0.5, 1, 2, 3, 4, 6, 8, 12, 24, and 48 h post-dose in each study period. Clinical laboratory tests were performed at pre-dose and 24 h post-dose in each study period.

No changes were made in the Statistical Analysis Plan (Version TS071-02-11-01 dated October 5, 2011) and Statistical Analysis Plan for Digital ECGs (dated October 17, 2011).

## Results

### Subject demographics

**Fig 1:**
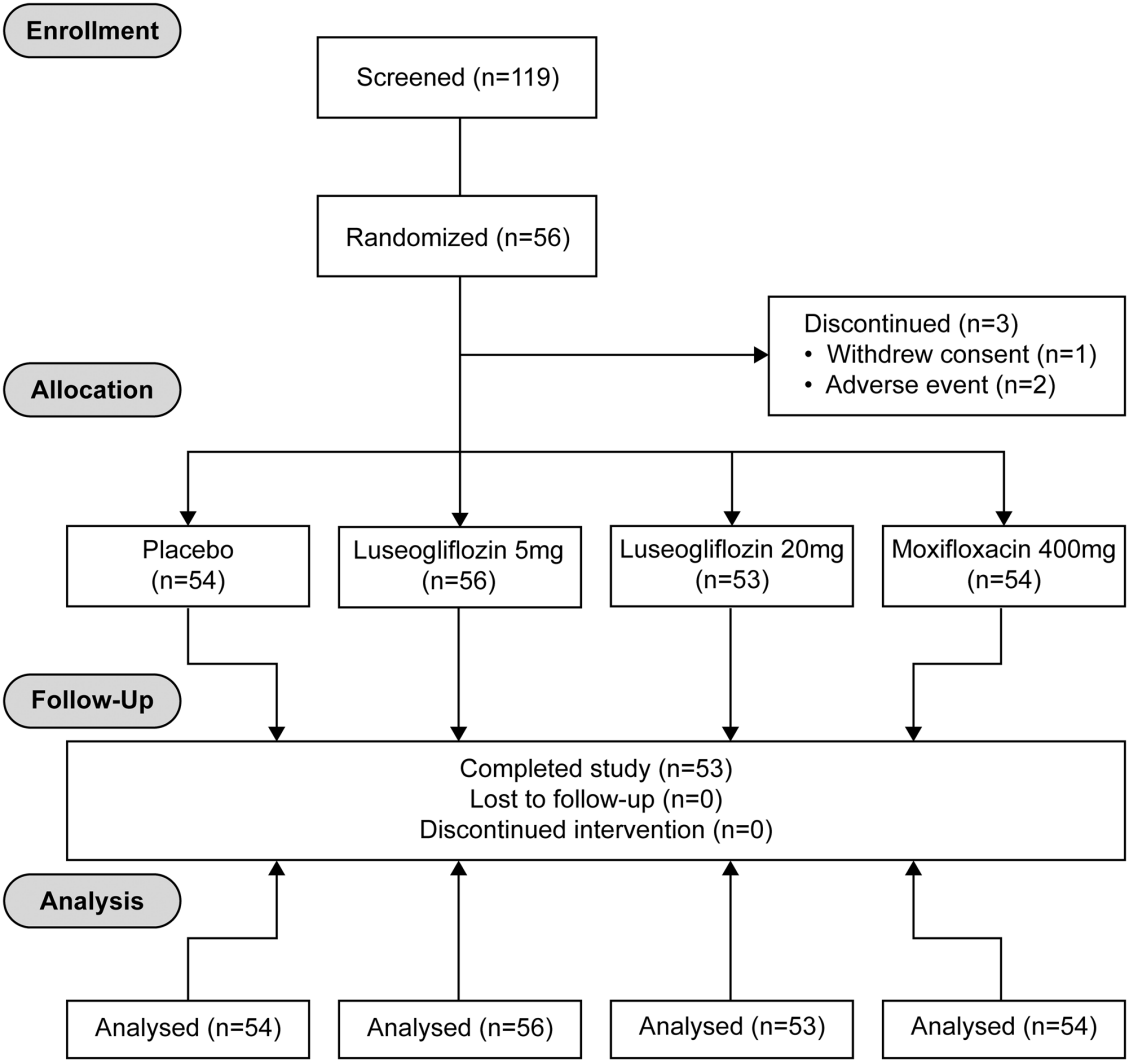
CONSORT 2010 Flow Diagram. Study design: A randomized, single dose, 4-period crossover study consisting of 4 treatment sequences.

A total of 56 Japanese subjects (28 male and 28 female) ranging in age from 20 to 44 years, in body weight from 43.2 to 77.5 kg, and in BMI from 18.5 to 24.2 kg/m^2^ were enrolled into this study. Age and BMI were similar between males and females (Table 1) (Fig 1). Fifty-three subjects completed the study. Three subjects discontinued the study prematurely due to adverse events (2 subjects) and withdrawal of consent (1 subject).

**Table 1:** Baseline Characteristics.

	Male	Female	Total
N	28	28	56
**Age, years**	31.1 ± 6.6	31.5 ± 7.5	31.3 ± 7.0
**Body weight, kg**	62.85 ± 7.31	52.68 ± 5.84	57.76 ± 8.32
**BMI, kg/m** ^**2**^	21.18 ± 1.58	20.88 ± 1.44	21.03 ± 1.51

Mean ± S.D.

### QTc interval

**Fig 2:**
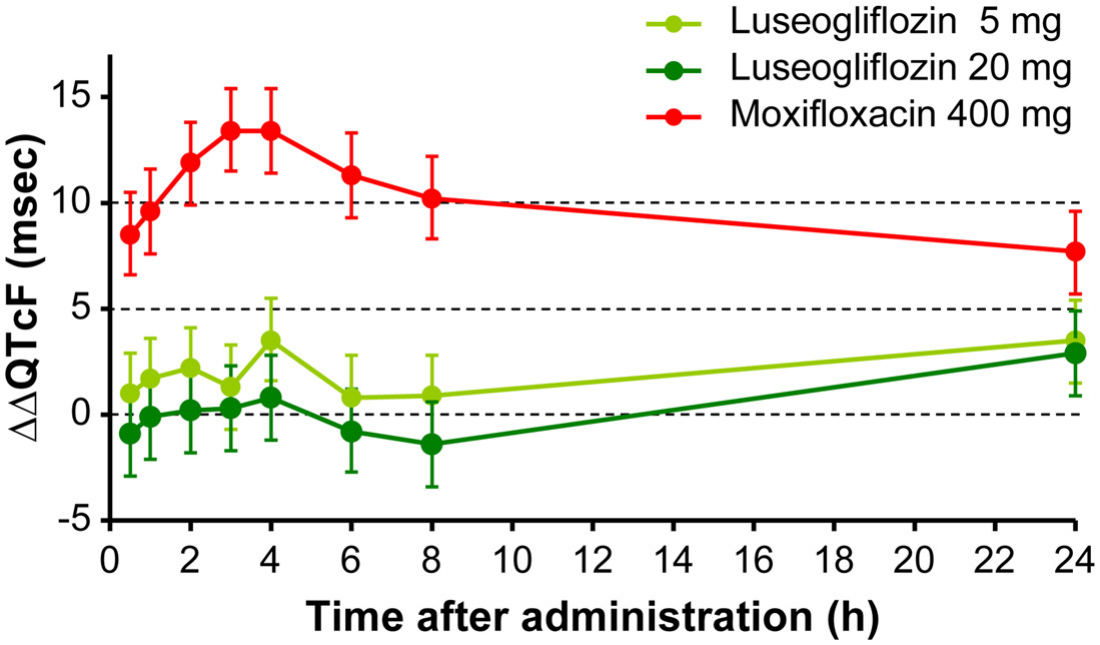
Least-squares mean difference and 90% CI of ΔΔQTcF. Abbreviation: CI, confidence interval. Serial triplicate digital 12-lead electrocardiograms (ECGs) were recorded for Luseogliflozin 5 mg, Luseogliflozin 20 mg, and Moxifloxacin 400 mg at 0.5, 1, 2, 3, 4, 6, 8, and 24 hours post-dose. Y-axis represents ΔΔQTcF (msec) and X-axis represents post-dose time points (0.5, 1, 2, 3, 4, 6, 8, and 24 hours), when ECGs were recorded. Least-squares mean difference and 90% CI of ΔΔQTcF were represented by a dot and a vertical bar, respectively.

The upper bound of the 90% two-sided CI for the least squares mean ΔΔQTcF was below the 10 ms threshold at all evaluation time points for both the therapeutic dose (5 mg) and supratherapeutic dose (20 mg) of luseogliflozin. For the moxifloxacin group, the least squares mean ΔΔQTcF ranged from 7.7 to 13.4 ms, with the lower bound of the corresponding 90% two-sided CI ranging from 5.7 to 11.5 ms, which establishes assay sensitivity for the study (Fig 2). ΔΔQTcB was analyzed similarly. For both the anticipated therapeutic dose and supratherapeutic dose, the upper bounds of the 90% two-sided CI of the least squares mean ΔΔQTcB values were below the 10 ms threshold at all evaluation time points (data not shown).

### Pharmacokinetic–pharmacodyamic relationship

**Fig 3:**
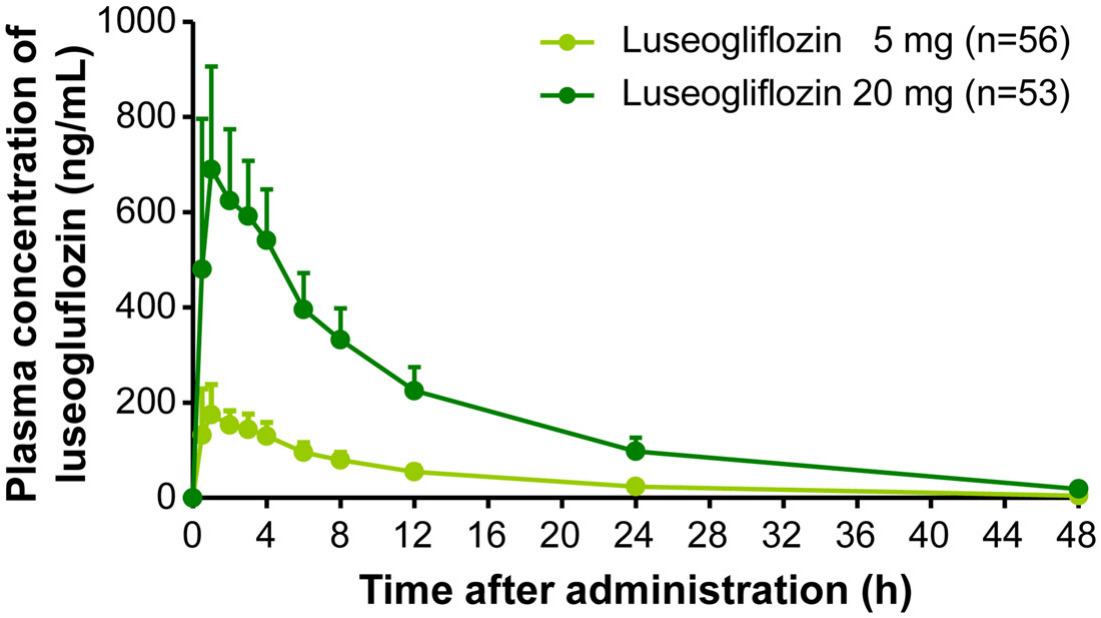
Plasma concentrations of luseogliflozin (mean + S.D.) Abbreviation: S.D., standard deviation. Plasma concentration of Luseogliflozin 5 mg and Luseogliflozin 20 mg was measured at 0.5, 1, 2, 3, 4, 6, 8, 24, and 48 hours post-dose. Y-axis represents plasma concentration of Luseogliflozin (ng/mL) and X-axis represents plasma concentration of Luseogliflozin measurements at 0.5, 1, 2, 3, 4, 6, 8, 24, and 48 hours post-dose. Here, mean and S.D. were represented by a dot and a vertical bar (upper bound), respectively.

**Fig 4:**
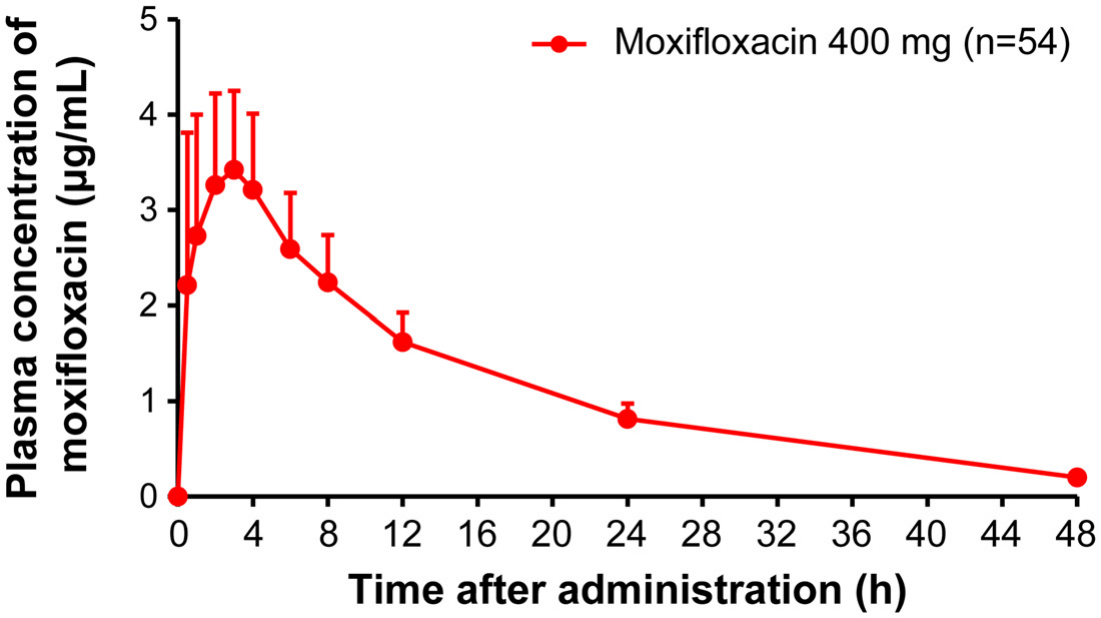
Plasma concentrations of moxifloxacin (mean + S.D.) Abbreviation: S.D., standard deviation. Plasma concentration of Moxifloxacin 400 mg was measured at 0.5, 1, 2, 3, 4, 6, 8, 24, and 48 hours post-dose. Y-axis represents plasma concentration of Moxifloxacin (μg/mL) and X-axis represents plasma concentration of Moxifloxacin measurements at 0.5, 1, 2, 3, 4, 6, 8, 24, and 48 hours post-dose. Here, mean and S.D. were represented by a dot and a vertical bar (upper bound), respectively.

**Fig 5:**
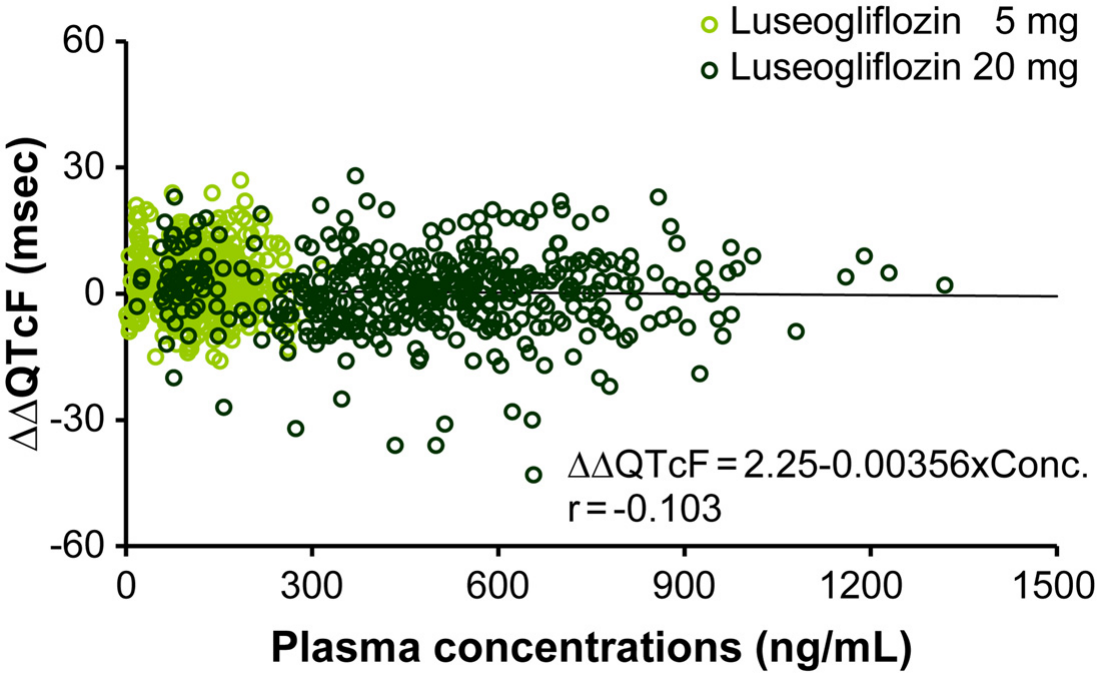
Scatter plots of ΔΔQTcF and plasma concentrations of luseogliflozin. Abbreviation: r, correlation coefficient. Here, scatter plot represents correlation between ΔΔQTcF and plasma concentrations of Luseogliflozin 5 mg and 20 mg doses. Y-axis represents ΔΔQTcF (msec) and X-axis represents plasma concentration of Luseogliflozin (ng/mL).

**Fig 6:**
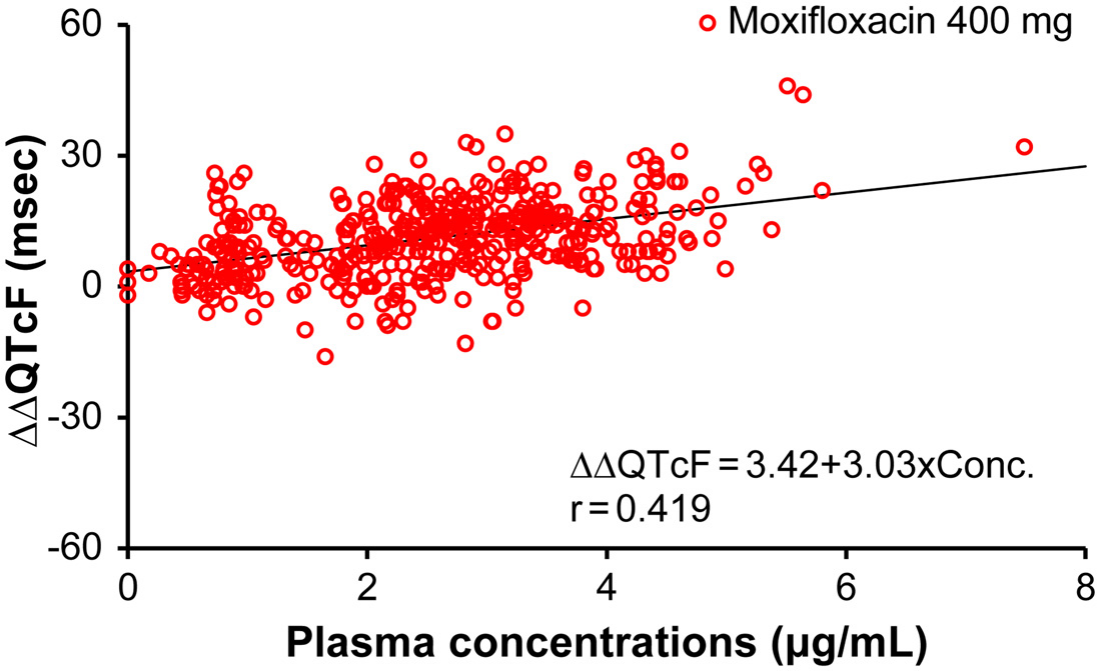
Scatter plots of ΔΔQTcF and plasma concentrations of moxifloxacin. Abbreviation: r, correlation coefficient. Here, scatter plot represents correlation between ΔΔQTcF and plasma concentrations of Moxifloxacin 400 mg. Y-axis represents ΔΔQTcF (msec) and X-axis represents plasma concentration of Moxifloxacin (μg/mL).

Pharmacokinetic parameters are summarized in Table 2. After administration of luseogliflozin 5 or 20 mg, plasma luseogliflozin concentrations increased dose dependently. Luseogliflozin and moxifloxacin were absorbed rapidly after oral administration (Figs 3 and 4). T_max_ was 1.3, 1.5, and 2.2 h for luseogliflozin 5 mg, 20 mg, and moxifloxacin 400 mg, respectively. No meaningful correlation was observed between ΔΔQTcF and plasma concentration of luseogliflozin unchanged compound, despite a positive correlation between ΔΔQTcF and plasma concentrations of moxifloxacin (Figs 5 and 6). The plasma concentration of luseogliflozin metabolites (M2, M17) was consistent with the result of a previous study conducted in healthy subjects and patients with type 2 diabetes (unpublished data).

**Table 2:** Pharmacokinetic parameters of luseogliflozin and its metabolites (M2, M17) and moxifloxacin.

Analyte	Treatment*	Cmax (ng/mL)**		Tmax (h)		AUCinf (ng·h/mL)***		T1/2 (h)	
		N	Mean ± S.D.	N	Mean ± S.D.	N	Mean ± S.D.	N	Mean ± S.D.
**Luseogliflozin**	L 5 mg	56	209 ± 48.9	56	1.34 ± 0.935	56	2110 ± 418	56	10.0 ± 1.18
**Unchanged compound**	L 20 mg	53	787 ± 186	53	1.50 ± 0.961	53	8650 ± 1780	53	9.90 ± 1.22
**Metabolites**	L 5 mg	55	8.85 ± 1.95	55	5.71 ± 2.14	55	257 ± 48.3	55	13.8 ± 2.61
**(M2)**	L 20 mg	52	35.1 ± 7.36	52	5.83 ± 1.90	52	1030 ± 212	52	13.9 ± 2.53
**Metabolites**	L 5 mg	56	6.02 ± 2.10	56	4.89 ± 1.14	56	120 ± 41.9	56	10.9 ± 1.49
**(M17)**	L 20 mg	53	24.3 ± 9.98	53	5.13 ± 1.14	53	487 ± 196	53	10.9 ± 1.71
**Moxifloxacin unchanged compound**	M 400 mg	54	3.88 ± 0.939	54	2.19 ± 1.09	54	60.0 ± 11.1	54	12.0 ± 1.45

*L 5 mg: Luseogliflozin 5mg, L 20 mg: Luseogliflozin 20 mg, M 400 mg: Moxifloxacin 400 mg

** Moxifloxacin unchanged compound (μg/mL)

*** Moxifloxacin unchanged compound (μg·hr/mL)

### QT hysteresis

**Fig 7:**
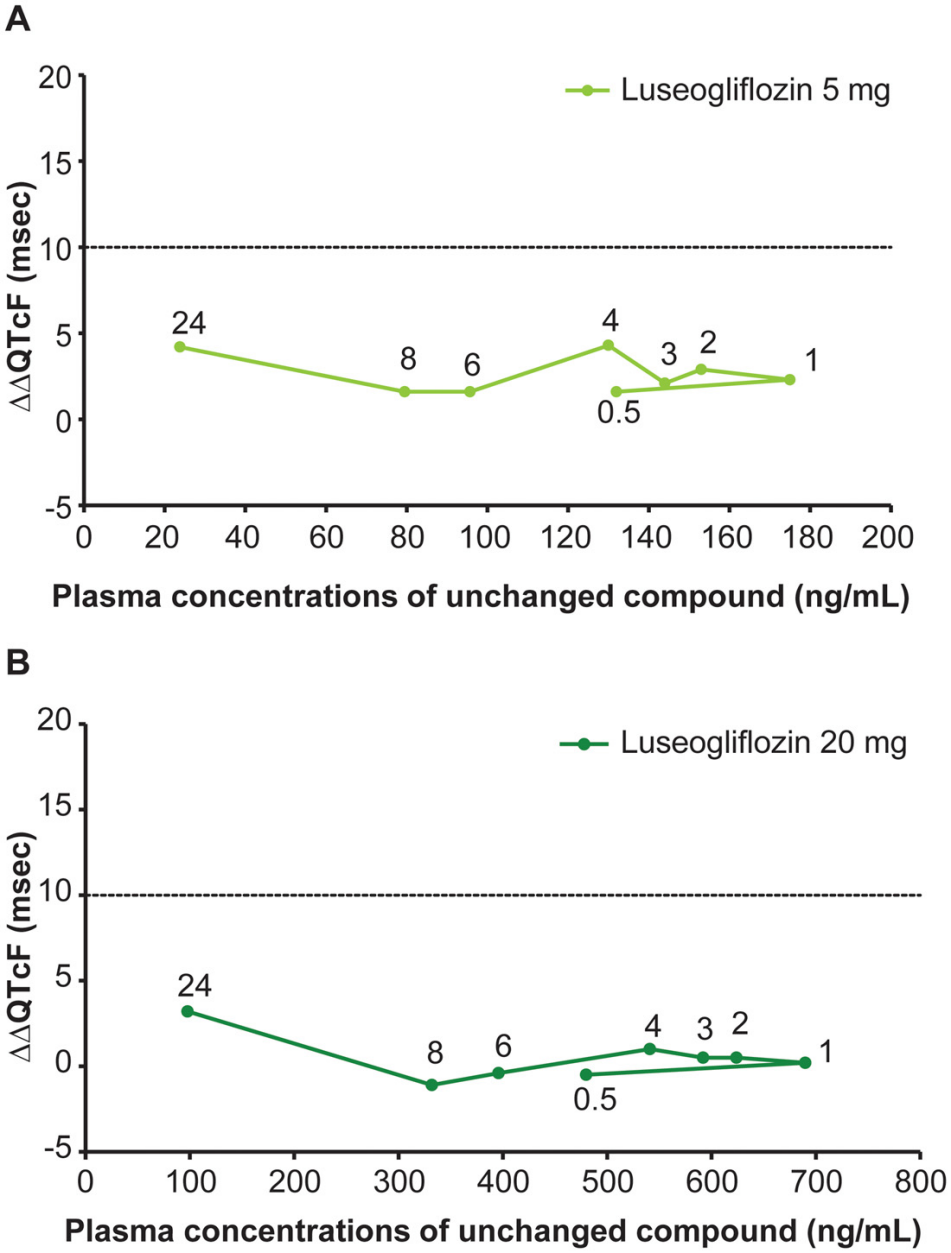
Hysteresis plots of ΔΔQTcF and plasma concentrations of luseogliflozin 5 mg and 20 mg. Here, Y-axis represents ΔΔQTcF (msec) and X-axis represents plasma concentration of unchanged Luseogliflozin (ng/mL). ΔΔQTcF (msec) was recorded at 0.5, 1, 2, 3, 4, 6, 8, and 24 hours post-dose.

**Fig 8:**
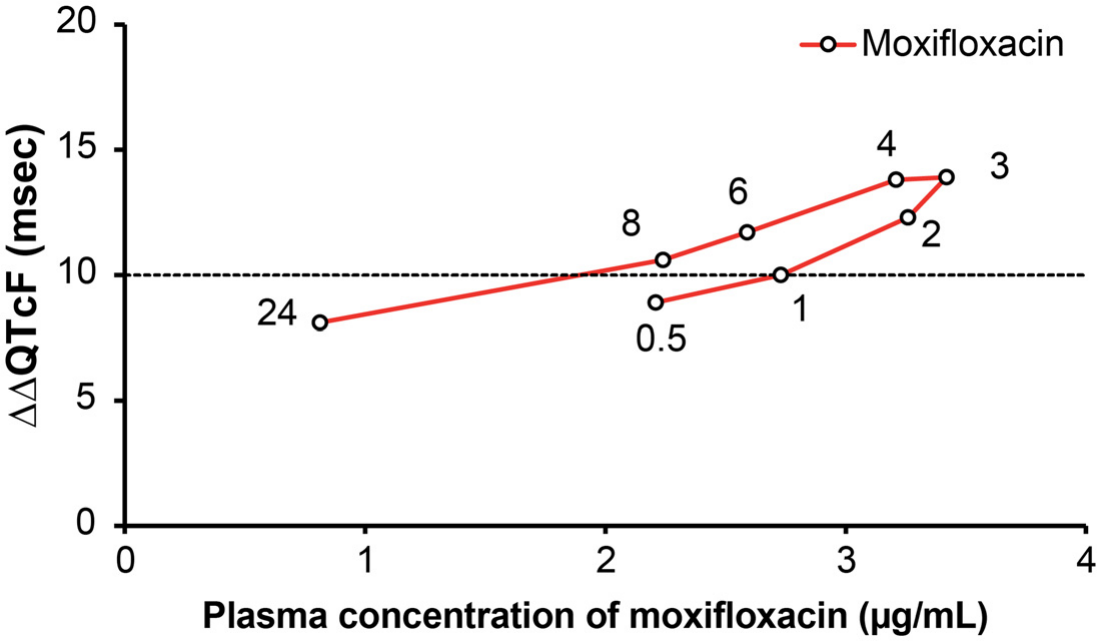
Hysteresis plots of ΔΔQTcF and plasma concentrations of moxifloxacin. Here, Y-axis represents ΔΔQTcF (msec) and X-axis represents plasma concentration of unchanged Moxifloxacin (μg/mL). ΔΔQTcF (msec) was recorded at 0.5, 1, 2, 3, 4, 6, 8, and 24 hours post-dose.

The plots of mean plasma concentrations of luseogliflozin unchanged compounds and mean ΔΔQTcF at each time point after administration demonstrated no clear hysteresis effect for either luseogliflozin dosage group (Fig 7). However, mean plasma concentrations of moxifloxacin unchanged compound and mean ΔΔQTcF at each time point after administration demonstrated a counter-clockwise hysteresis effect (Fig 8).

### Categorical analysis and cardiac abnormalities

In the categorical analysis, the number (%) of subjects who had a QTcF value greater than 450 ms after study drug administration was 1 of 56 subjects (1.8%) in the anticipated therapeutic dose group, 7 of 54 subjects (13.0%) in the moxifloxacin group, and 1 of 54 subjects (1.9%) in the placebo group. No subjects in any treatment group had a ΔQTcF value greater than 30 ms (Table 3). Cardiac abnormalities including abnormal T wave were observed in all treatment groups; however, the rate of cardiac abnormalities was lower in the anticipated therapeutic dose and supratherapeutic dose groups compared to the placebo group. The correlation coefficient for QTcF versus RR was smaller than that for QTcB or QT versus RR, suggesting that QT was corrected appropriately by Fridericia’s formula.

**Table 3:** Absolute QTcF interval prolongation and shift from baseline to the maximum QTcF.

Category	Number (%) of Subjects			
	Luseogliflozin	Luseogliflozin	Moxifloxacin	
	5 mg	20 mg	400 mg	Placebo
	(n = 56)	(n = 53)	(n = 54)	(n = 54)
**Value >500 ms**	0 (0.0)	0 (0.0)	0 (0.0)	0 (0.0)
**Value >480 ms**	0 (0.0)	0 (0.0)	0 (0.0)	0 (0.0)
**Value >450 ms**	1 (1.8)	0 (0.0)	7 (13.0)	1 (1.9)
Increase >60 ms	0 (0.0)	0 (0.0)	0 (0.0)	0 (0.0)
Increase >30 ms	0 (0.0)	0 (0.0)	0 (0.0)	0 (0.0)

### Safety

A single dose of luseogliflozin 5 mg and 20 mg was well tolerated. No serious adverse events occurred in the study. Two (2) subjects discontinued the study prematurely due to adverse events. One subject experienced mild pyrexia, rash, cheilitis and pruritus, and the other experienced first-degree atrioventricular block. The number (%) of subjects with an adverse event was as follows: 23 (41.1%) in the luseogliflozin 5 mg group, 19 (35.8%) in the luseogliflozin 20 mg group, 14 (25.9%) in the placebo group, and 39 (72.2%) in the moxifloxacin 400 mg group (Table 4). All the adverse events were reversible and mild in severity, except two events that was moderate in severity reported in the 5 mg luseogliflozin group. The incidence rates of adverse events and adverse drug reactions were higher in the 400 mg moxifloxacin group compared to any other treatment groups, and there were no clinically meaningful differences between the therapeutic-dose (5 mg) and supratherapeutic-dose (20 mg) luseogliflozin groups. Common adverse events, defined as occurring in two or more subjects in either of the luseogliflozin dose groups, were diarrhea, increased blood glucose, presence of blood urine, presence of urine ketone bodies, abnormal urinary sediment, and headache.

**Table 4:** Summary of adverse events (AEs).

		Luseogliflozin		Moxifloxacin
	Placebo	5 mg	20 mg	400 mg
	(n = 54)	(n = 56)	(n = 53)	(n = 54)
**Subjects with any AEs, n**	14	23	19	39
**AEs, n** *	17	48	32	56
**Drug-related AEs, n** *	7	20	16	36
**Frequent AEs**				
**(events reported in 2 subjects or more at any dose group), n**				
**Diarrhea**	5	6	3	4
**Nausea**	0	1	0	2
**Blood glucose increased**	1	1	2	1
**Prolonged QT**	0	1	1	23
**Blood urine present**	3	5	3	7
**Urine ketone body present**	1	5	7	1
**Urinary sediment abnormal**	4	8	3	7
**Headache**	1	4	2	4

*: number of events

The presence of urine ketone bodies was more frequent in the luseogliflozin groups. All events were mild in severity, requiring no treatment or action, and most of them were transient fluctuations observed only at 24 h after dosing. As cardiovascular adverse events, one subject in the 5 mg luseogliflozin group experienced first-degree atrioventricular block, and one subject each in the 5 mg and 20 mg luseogliflozin groups and 23 in the 400 mg moxifloxacin group experienced electrocardiogram QT prolongation. First-degree atrioventricular block was identified as the expression of prolonged PR interval to 452 ms at 48 h after administration of luseogliflozin 5 mg. This event was mild in severity and required no action or treatment; however, the subject was withdrawn from the study.

All cardiovascular adverse events reported following administration of luseogliflozin were assessed as not related to the study drug.

## Discussion

Diabetes mellitus is an important risk factor for cardiovascular diseases [7]. Although a decrease in morbidity and mortality from cardiovascular diseases is expected during the treatment of diabetes, drug therapies do not always decrease the morbidity of cardiovascular diseases, and some therapies increase the risk of these diseases. Some commonly used drugs have QT prolonging effects, which may consequently cause lethal arrhythmia. In addition, some diabetes drugs may also have potential QT prolonging effects [8, 11]. Therefore, cardiac safety evaluation is particularly important for diabetes drugs. Currently, new drug development requires examination of proarrhythmic effects via thorough QT (TQT) investigation for almost all drugs. Despite lagging behind the USA and EU, Japan fully adopted the ICH E14 guidelines in 2010 and has begun to conduct TQT studies. To the best of our knowledge, this investigation is the first placebo- and positive-controlled thorough QT/QTc study of an oral hypoglycemic agent performed in Japan. In this study, a supratherapeutic dose of luseoflozin (20 mg) was used at four times the clinical dose (5 mg) [4, 12, 13]. The data obtained to date have confirmed that pharmacokinetic changes in this drug are small in elderly people [14] as well as people with renal [15] and hepatic disorders, and that this drug has no interaction with other oral hypoglycemic agents or diuretics [16]; we believe that the supratherapeutic dose used in this study can fully encompass the maximum exposure expected in the clinical use. Moxifloxacin 400 mg, which has been used in many reports, served as a positive control [17, 18]. Although the ICH E14 guidelines state that ethnic factors are not expected to have an impact on the results of QT/QTc studies, the question remains as to whether specific ethnic studies are required to assess cardiac safety in Japanese subjects. For example, the QT prolonging effects of quinidine were observed to differ between Koreans and Caucasians [19], and the QT prolonging effects of norfloxacin demonstrated a slight difference between Japanese and Caucasians [20]. Therefore, whether moxifloxacin hydrochloride can be used as a positive control for TQT studies in Japanese subjects was one of our interests in this study.

The upper bound of the 90% two-sided CI for ΔΔQTcF was below 10 ms at all time points for both therapeutic and supratherapeutic doses of luseogliflozin, while the lower bound of the 90% two-sided CI for ΔΔQTcF of moxifloxacin was greater than 0 ms at all time points. These findings indicate that luseogliflozin does not affect QT prolongation in a clinical setting within the limits of assay sensitivity. Pharmacokinetic-pharmacodynamic analyses also revealed no relationship between plasma concentration of luseogliflozin and changes in QT interval. This observation indicates that luseogliflozin did not demonstrate a significant proarrhythmic effect.

When conducting TQT studies, ICH E14 guidance recommends using a positive control group to establish assay sensitivity. The positive control should have an effect on the mean QT/QTc interval of approximately 5 ms. Detecting the effect of the positive control on QT/QTc interval is important in terms of assuring the ability of the study to detect such an effect of the study drug. Japan has less experience in TQT studies, prompting some discussion concerning an appropriate positive control in Japanese subjects; in this study, we used moxifloxacin 400 mg, which had been previously reported as a positive control. We found that the effect of moxifloxacin on QT/QTc interval was sufficiently high in Japanese healthy subjects. The lower limit of the two-sided 90% CIs of the baseline adjusted and placebo adjusted mean QTcF (ΔΔQTcF) was more than 5 ms at all time points. The least squares mean ΔΔQTcF values for moxifloxacin ranged from 7.7 ms (24 h post-dose) to 13.4 ms (3 and 4 hour post-dose). Generally, QT interval-prolonging effects of moxifloxacin are supposed to relate linearly to serum concentrations [21]; in this study, however, counterclockwise hysteresis was observed in this relationship, which could be a reflection of differences in ethnicity. However, as the hysteresis loop was small and a linear correlation was also observed, the hysteresis effects may be attributed to factors such as the timing of measurements, method of measurement, and variability.

Based on the results of this study, the sufficient number of cases to demonstrate assay sensitivity associated with moxifloxacin is much smaller than that which has been commonly adopted. If the same design was used, the number of subjects could be reduced in a study in Japan, which requires further accumulation of data.

The limitation of a thorough QT/QTc study is to predict the frequency of rare cardiovascular events such as arrhythmia with mean change of QT/QTc interval which is recognized as imperfect biomarker for the potential occurrence of drug-induced TdP. Therefore prolongation of a QTc interval is not directly associated with cardiovascular events. The adverse events that occurred in this study were all transient and mild with no findings associated with safety problems. One subject withdrew from the study due to a first-degree atrioventricular block. This event was determined to be unrelated to the study drug because it developed 48 hours after administration, thus there was no temporal relationship between the event and the dose, considering the pharmacokinetic profile of luseogliflozin and its metabolites.

### Summary

The design of this thorough QT study, whose population is healthy Japanese subjects, is typical based on ICH E14 guidance. This study suggests that luseogliflozin carries no concerns for possible QT prolonging effects and demonstrates that moxifloxacin can be used as a positive control in Japanese thorough QT studies.

## Conclusion

A single dose of luseogliflozin 5 mg or 20 mg was safe and well tolerated in Japanese healthy male and female subjects. This study was established as a negative thorough QT/QTc study based on the development of rigorous assay sensitivity, as well as observation of moxifloxacin activity. No arrhythmogenic effects of either therapeutic (5 mg) or supratherapeutic (20 mg) doses of luseogliflozin were observed in healthy Japanese subjects.

## Supporting Information

S1 Appendix CONSORT Checklist. (DOC)

S2 Appendix Luseogliflozin TQT study protocol. (PDF)
