# Supplementary material for: Randomized, Controlled, Thorough QT/QTc Study Shows Absence of QT Prolongation with Luseogliflozin in Healthy Japanese Subjects
Source: PLoS One. 2015 Oct 7;10(10):e0139873. doi: 10.1371/journal.pone.0139873 (PMC4596473; doi:10.1371/journal.pone.0139873)
Supplement: S2 Appendix — (PDF) [file pone.0139873.s002.pdf]

The Thorough QT/QTc Study of TS-071 in Healthy Subjects

Protocol

Taisho Pharmaceutical Co., Ltd.

Protocol No.: TS071-02-11

Version No.: Ver. 2 *(translation of Japanese protocol Ver. 2 dated June 20, 2011)*

Date of preparation: June 23, 2011

Confidential
The information contained in this document is in the possession of Taisho Pharmaceutical Co., Ltd. and is provided to the parties that are involved in this clinical study including principal investigators, subinvestigators, clinical research coordinators, study sites and institutional review boards. Therefore, it cannot be disclosed to any other parties, except for the subjects who will participate in this study, without prior written consent of Taisho Pharmaceutical Co., Ltd. Besides, a part or whole of the results obtained in this clinical study may not be published at academic meetings or in scientific journals without prior written approval of Taisho Pharmaceutical Co., Ltd.

PROTOCOL SYNOPSIS

1. Study Objectives

The objective of this study is to evaluate the effect of TS-071 on QT/QTc interval in healthy subjects.

2. Study Subjects

Healthy Japanese male and female volunteers who meet the inclusion criteria and do not meet any of the exclusion criteria will be eligible for the study.

2.1 Inclusion Criteria

A subject must meet all of the following criteria:

(1) The subject is aged ≥ 20 and < 45 years at the time of giving informed consent.

(2) The subject's body mass index (BMI) is ≥ 18.5 and < 25.0, and weight is ≥ 40 kg at the screening examination.

(3) The subject's resting pulse rate is ≥ 45 and ≤ 90 beat/min at the screening examination and prior to study drug administration in Period 1.

(4) The subject is eligible for this study as assessed by the principal investigator or subinvestigator based on the screening and pre-treatment examinations up to prior to study drug administration in Period 1*.

(5) The subject is provided with the study procedures prior to participation in the study and is capable of understanding the study and gives his/her own written informed consent.

*: These are subjects who have no abnormalities in the screening as well as pre-treatment examinations up to prior to study drug administration including physical examination, body temperature, blood pressure, pulse rate and 12-lead ECG, and whose clinical laboratory tests at screening and Day -1 (day before study drug administration) in Period 1 are within the reference range of the study site. However, subjects with abnormal findings or values outside the reference range may be enrolled in the study if the findings or values are of no clinical significance and the subjects are eligible as medically assessed by the principal investigator or subinvestigator considering all other related examinations and findings. In that case, the reason for having enrolled such a subject in the study will be given in the case report form.

2.2 Exclusion Criteria

Any subject who meets any of the following criteria must be excluded:

(1) The subject has hepatic/biliary, respiratory, cardiovascular, gastrointestinal, urological, renal, endocrine or immunological disorder, malignant tumor, diabetes mellitus or impaired glucose tolerance, or has a history thereof within the previous 5 years prior to giving informed consent.

(2) The subject has a congenital disorder or cardiac disorder, or has a history thereof.

(3) The subject has a risk factor for or history of torsade de pointes (TdP), e.g., heart failure, hypokalemia

and a family history of Long QT Syndrome.

(4) The subject has a history of attack of unconsciousness possibly associated with TdP.

(5) The subject's 12-lead ECG waveform at screening and prior to study drug administration in Period 1 is not suitable for the evaluation of QT/QTc interval prolongation, e.g., drift, myogram, morphology of T wave, remarkable sinus arrhythmia and frequent extrasystoles.

(6) The subject's QTcF (QT interval corrected by Fridericia formula) in 12-lead ECG at screening and prior to study drug administration in Period 1 is ≥ 450 msec.

(7) The subject falls into any of the following criteria related to renal function:

・Serum creatinine at screening or prior to study drug administration in Period 1 exceeds the upper limit of reference range of the study site;

・Urinary protein at screening and prior to study drug administration in Period 1 is positive (≥ +1);

・Urinary occult blood at screening and prior to study drug administration in Period 1 is positive (≥ +1), with an exception when the positive result is obviously for a non-disease reason such as emmenia.

(8) The subject received an administration of any other study drug within the previous 16 weeks prior to giving informed consent.

(9) The subject previously received an administration of TS-071 tablet (active drug).

(10) The subject donated blood or underwent blood collection as described below:

Donation or collection of blood component within 2 weeks prior to the screening;

Donation or collection of < 400 mL of blood within 4 weeks prior to the screening;

Donation or collection of ≥ 400 mL of blood within 12 weeks prior to the screening for a male subject and within 16 weeks prior to the screening for a female subject

(11) The subject used any medication including an over-the-counter medication within one week prior to study drug administration in Period 1 except lacrimal fluid (eye lotion) for contact lenses.

(12) The subject is unable to abstain from alcohol drinking within 2 days prior to admission to the study site in each study period through discharge, and within 2 days prior to post-treatment examination through its completion.

(13) The subject smokes or smoked within 6 months prior to giving informed consent.

(14) If female, the subject is pregnant, lactating, possibly pregnant, intending to become pregnant during the course of the study, or shows positive pregnancy test at screening or prior to study drug administration in Period 1.

(15) The subject is not capable of using an adequate contraception after giving informed consent through completion of the post-treatment examination.

(16) The subject has a history of allergy to drug or food.

(17) The subject has a significant allergic diathesis, e.g., asthma requiring medical intervention.

(18) The subject is alcohol or drug dependence or a history thereof.

(19) The subject has a positive urine drug screen (phencyclidines, benzodiazepines, cocaines, stimulants, cannabis, morphines, barbiturates and tricyclic antidepressants) at screening.

(20) The subject has a positive result for HBs antigen, HCV antibody, HIV antigen, HIV antibody or serologic test for syphilis at screening.

(21) The subject is, in the principal investigator's or subinvestigator's opinion, unsuitable for any other

reason.

3. Study Drugs

3.1 Development Code

TS-071 (Drug substance identification code: SGL0176 hydrate)

3.2 Dosage Form and Contents

Study drug		Dosage form and contents
Test drug	TS-071 tablets 5 mg	White to grayish white film-coated tablets containing 5 mg of SGL0176 per tablet
Control drug	TS-071 tablets placebo	White to grayish white film-coated tablets containing no SGL0176 or other medicinal properties that are identical in appearance to TS-071 tablets 5 mg
Control drug (positive control)	Avelox® tablets	Pale reddish gray film-coated tablets with a cleavage containing 400 mg of moxifloxacin (436.8 mg as moxifloxacin hydrochloride)

4. Study Design

4.1 Study Design

This is a randomized, single dose, 4-period crossover study based on a Williams design consisting of 4 treatment sequences. Each treatment sequence comprises of 4 treatment groups, i.e., anticipated therapeutic dose, supratherapeutic dose, placebo and Avelox.

- Placebo and Avelox (positive control) treatment groups are included as controls.
- Treatments of anticipated therapeutic dose, supratherapeutic dose and placebo will be double-blinded.
- Treatment of Avelox will not be blinded.
- 12-lead ECG data for QT/QTc analysis will be blinded for all the treatments to the ECG readers at the central ECG analysis facility.

4.2 Study Procedures and Period

The study will be conducted according to the flowchart below:

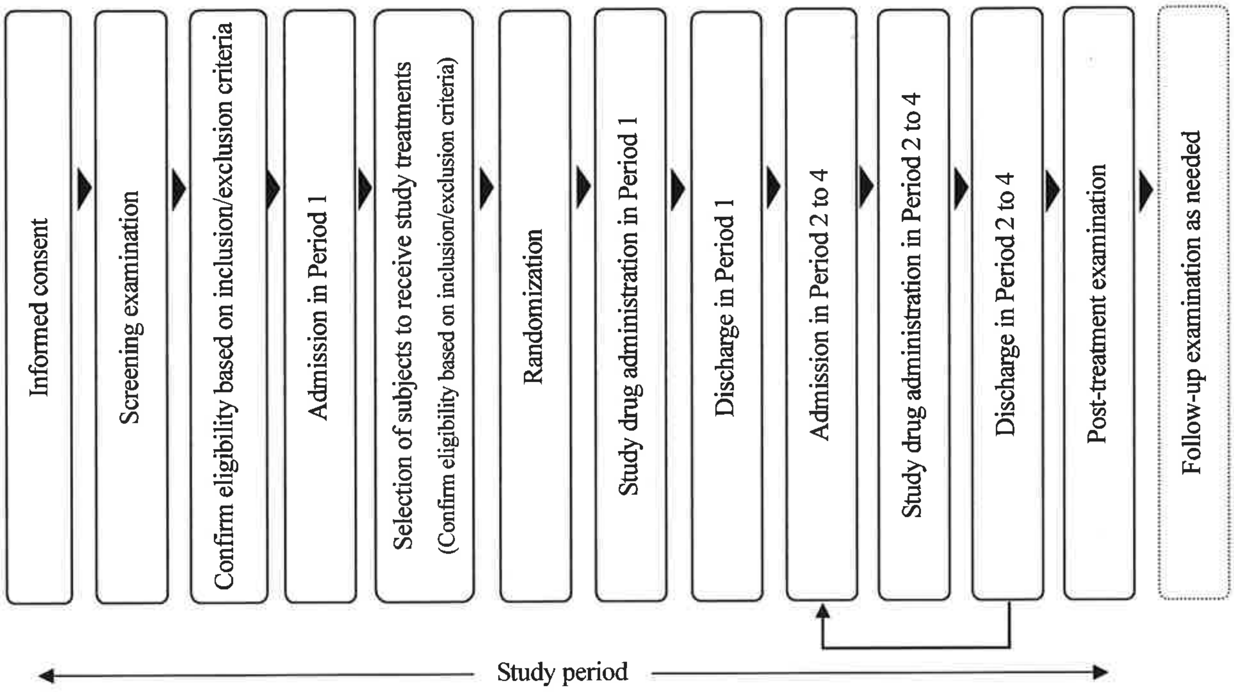

Duration of hospitalization will be 4 days and 3 nights.
Post-treatment examination will be conducted with an interval of ≥ 7 days after study drug administration in Period 4 (on Day 8 or thereafter when Day 1 is the day of study drug administration in Period 4).

4.3 Dose and Dosage Regimen

Dose of each treatment group will be as follows:

Anticipated therapeutic dose group: TS-071 5 mg
Supratherapeutic dose group: TS-071 20 mg
Placebo group: TS-071 placebo
Avelox group: moxifloxacin 400 mg

Subjects will receive a single oral dose of a study drug assigned in each study period with 200 mL of water in the fasted state in the morning of study drug administration day according to the randomization code. There will be a wash-out interval of ≥ 7 days between each study period, i.e., when the preceding dose is on Day 1, the subsequent dose will be on Day 8 or thereafter.

4.4 Study Period

From June 13, 2011 to October 31, 2011

4.5 Number of Subjects to be Enrolled

Fifty-six (56)

4.6 Endpoints

4.6.1 Variables for QT/QTc Interval Analysis

The primary endpoint is QTcF, QT interval corrected by Fridericia's formula, and the secondary endpoint is QTcB, QT interval corrected by Bazett's formula.

4.6.2 Safety Variables

(1) Adverse events and adverse drug reactions, including abnormal changes in body temperature, blood pressure, pulse rate, 12-lead ECG and clinical laboratory tests
(2) Effects on body temperature, blood pressure, pulse rate, 12-lead ECG and clinical laboratory tests

4.6.3 Pharmacokinetic Variables

Plasma concentrations of unchanged TS-071 and its metabolites (M-2 and M-17), and plasma concentrations of unchanged moxifloxacin

5. Study Procedures

5.1 Screening Procedures

The principal investigator or subinvestigator will obtain written informed consent from candidate subjects and perform the screening examination as described below within one to 28 days prior to study drug administration in Period 1, and select eligible subjects for the study. Height, weight, body temperature, blood pressure and pulse rate will be measured and blood and urine samples for clinical laboratory tests and other procedure will be collected, measured and stored in accordance with the procedures of the study site.

Screening Examination

Physical examination		Subjective symptoms, objective findings (inspection, auscultation, percussion, palpation) and medical interview (medical history, present illness and its history, family history, drug treatment history, allergy history, previous clinical study participation, previous blood donation/collection, drinking status, smoking status, other)
Height, weight, body temperature, blood pressure, pulse rate		Height, weight, (BMI*1), body temperature*2, blood pressure*3, pulse rate*3
ECG		12-lead ECG*4 (RR, PR, QRS, QT, QTcF)
Clinical laboratory tests	Hematology tests	White blood cell count, red blood cell count, hemoglobin, hematocrit, platelet count, WBC differentials (neutrophils, lymphocytes, monocytes, eosinophils, basophils)
	Blood chemistry tests	Total bilirubin, direct bilirubin, AST(GOT), ALT(GPT), AL-P, LDH, γ-GTP, CK(CPK), total protein, albumin, A/G ratio, BUN, uric acid, creatinine, cystatin C, total cholesterol, LDL cholesterol, HDL cholesterol, triglycerides, glucose, Ca, P, Na, K, Cl, Mg, CRP
	Urinalysis	Qualitative tests (urobilinogen, ketones, glucose, protein, occult blood, pH), sediment
Immunology tests		HBs antigen, HCV antibody, HIV antigen, HIV antibody, serologic test for syphilis
Urine drug screen		Phencyclidines, benzodiazepines, cocaines, stimulants, cannabis, morphines, barbiturates, tricyclic antidepressants
Pregnancy status (female only)		Pregnancy test

*1: BMI will be calculated as Weight (kg) / Height $(m)^2$ and rounded to one decimal place.
*2: Body temperature will be measured under armpit.
*3: Blood pressure and pulse rate will be measured at rest in the supine position.
*4: 12-lead ECG will be recorded in the supine position after resting for 5 min.

5.2 Evaluation of 12-Lead ECG Data for QT/QTc Analysis

12-lead ECG for QT/QTc analysis will be recorded according to a separate written procedure and recordings will be read at the central ECG analysis facility. The same model of 12-lead digital surface ECG monitor should be used for all the subjects at all the time points.

Three charts of 12-lead ECG for QT/QTc analysis will be recorded in 5 min for each time point shown in the table below. The interval between the start of each recording should be ≥ 1 min, and ECG will be recorded for 10 seconds for each chart. The acceptable time windows for recording ECG are given in "Appendix 3: Acceptable Time Windows for Examinations." All 12-lead ECG charts for QT/QTc analysis will be sent electronically by the responsible person of the study site to the central ECG analysis facility.

For all the ECG charts for QT/QTc analysis sent to the central ECG analysis facility, RR, QT, PR and QRS intervals will be measured in accordance with the procedure of the facility. The time of recording and subject identification information should be shielded before measurement. QTc intervals will be corrected by Fridericia's and Bazett's formulae.

The effect of TS-071 on QT/QTc interval prolongation will be regarded negative when the upper bound of the 95% one-sided confidence interval for the largest mean effect on the QT/QTc interval excludes 10 msec at all

the time points in the TS-071 groups.

In addition, pharmacokinetic parameters will be determined from plasma concentrations of unchanged TS-071 and moxifloxacin, and an exploratory investigation will be made to assess the relationship of drug concentrations to changes in QT/QTc interval.

Variable	Time after study drug administration
12-lead ECG for QT/QTc analysis*	Pre-dose and 0.5, 1, 2, 3, 4, 6, 8, 24 hr post-dose in each study period

*: 12-lead ECG will be recorded in the supine position after resting for 10 min.

5.3 Safety Assessments

The principal investigator or subinvestigator will conduct the following observations/examinations in accordance with "Appendix 2: Schedule of Study Procedures." The acceptable time window for each examination is given in "Appendix 3: Acceptable Time Windows for Examinations." Body temperature, blood pressure and pulse rate will be measured and blood and urine samples for clinical laboratory tests and other procedure will be collected, measured and stored in accordance with the procedures of the study site.

Variables for Safety Assessments

Physical examination		Subjective symptoms, objective findings (inspection, auscultation, percussion, palpation) and medical interview
Body temperature, blood pressure, pulse rate		Body temperature*1, blood pressure*2, pulse rate*2
ECG		12-lead ECG*3
Clinical laboratory tests	Hematology tests	White blood cell count, red blood cell count, hemoglobin, hematocrit, platelet count, WBC differentials (neutrophils, lymphocytes, monocytes, eosinophils, basophils)
	Blood chemistry tests	Total bilirubin, direct bilirubin, AST(GOT), ALT(GPT), AL-P, LDH, γ-GTP, CK(CPK), total protein, albumin, A/G ratio, BUN, uric acid, creatinine, cystatin C, total cholesterol, LDL cholesterol, HDL cholesterol, triglycerides, glucose, Ca, P, Na, K, Cl, Mg
	Urinalysis	Qualitative tests (urobilinogen, ketones, glucose, protein, occult blood, pH), sediment
Pregnancy status (female only)		Pregnancy test

*1: Body temperature will be measured under armpit.
*2: Blood pressure and pulse rate will be measured at rest in the supine position.
*3: 12-lead ECG will be recorded in the supine position after resting for 10 min.

5.4 Pharmacokinetic Measurements

Plasma concentrations of unchanged TS-071 and its metabolites (M-2 and M-17) and plasma concentrations of unchanged moxifloxacin will be determined. Blood will be collected at the time points shown in the table below and time of collection will be documented in the case report form (CRF). If blood collection coincides with 12-lead ECG recording, blood will be collected after ECG recording. Acceptable time windows for

blood collection are described in "Appendix 3: Acceptable Time Windows for Examinations." Collection, handling and shipment of blood specimens are detailed in a separate written procedure for the handling of specimens.

Variable*	Time after study drug administration
Plasma concentrations of unchanged TS-071<br>Plasma concentrations of TS-071 metabolites (M-2 and M-17)<br>Plasma concentrations of unchanged moxifloxacin	Pre-dose and 0.5, 1, 2, 3, 4, 6, 8, 12, 24, 48 hr post-dose in each study period

*: For the treatment groups of anticipated therapeutic dose and supratherapeutic dose, plasma concentrations of unchanged TS-071 and its metabolites will be determined; for the Avelox group, plasma concentrations of unchanged moxifloxacin will be determined.

5.5 Number of Blood Samplings and Volume of Blood to be Collected

The table below describes the number of blood samplings and blood volume to be collected from screening examination through post-treatment examination.

Examination	Volume of blood per time	Number of samplings	Blood volume
Screening examination	11 mL	1	11 mL
Hematology and blood chemistry tests, blood glucose	9 mL	10	90 mL
Pharmacokinetics (anticipated therapeutic dose group, supratherapeutic dose group, placebo group)	5 mL	33	165 mL
Pharmacokinetics (Avelox group)	7 mL	11	77 mL
Total			343 mL

List of Abbreviations and Definitions of Terms

Abbreviation	Definition
AUC	area under the plasma concentration-time curve
BMI	body mass index
Cmax	maximum plasma concentration
CRF	case report form
DPP-IV	dipeptidyl peptidase-IV
GCP	Good Clinical Practice
GLP-1	glucagons-like peptide-1
HBs	hepatitis B virus surface antigen
HCV	hepatitis C virus
HIV	human immunodeficiency virus
IC_{50}	50% inhibitory concentration
PTP	press-through package
SGLT	sodium-dependent glucose cotransporter
SGLT1	sodium-dependent glucose cotransporter 1
SGLT2	sodium-dependent glucose cotransporter 2
$T_{1/2}$	elimination half-life
TdP	torsade de pointes
Tmax	time to maximum plasma concentration
WHO	World Health Organization

Term	Definition
M-2	Derivative of SGL0176, in which ethyl in ethoxy group is substituted by a hydroxyl group.
M-17	Derivative of SGL0176, in which methyl in ethoxy group is substituted by a carboxyl group.
QTcB	QT interval corrected by Bazett's formula
QTcF	QT interval corrected by Fridericia's formula
ΔQTc	Change in QTc from baseline
ΔΔQTc	Difference in ΔQTc between TS-071 cohort or Avelox cohort and Placebo cohort
QT/QTc Guideline	The clinical evaluation of QT/QTc interval prolongation and proarrhythmic potential for non antiarrhythmic drugs (PFSB Notification 1023, No.1 dated October 23, 2009)
SGL0176 hydrate	Drug substance name of TS-071 tablets
SGL0176	Dose and concentration are presented as converted SGL0176 anhydrate.
TS-071 groups	Treatment groups receiving TS-071 active drug, i.e., anticipated therapeutic dose group and supratherapeutic dose group
Concentrations of unchanged TS-071	Drug concentrations as SGL0176 anhydride
Treatment sequence	An order of treatment, e.g., anticipated therapeutic dose, supratherapeutic dose, placebo and Avelox
Admission group	Group of subjects who are admitted to the study site and start study procedures on the same day
moxifloxacin	Ingredient of Avelox® tablet

Table of Contents

PROTOCOL SYNOPSIS i

1. Study Objectives i

2. Study Subjects i
  2.1 Inclusion Criteria i
  2.2 Exclusion Criteria i

3. Study Drugs iii
  3.1 Development Code iii
  3.2 Dosage Form and Contents iii

4. Study Design iii
  4.1 Study Design iii
  4.2 Study Procedures and Period iii
  4.3 Dose and Dosage Regimen iv
  4.4 Study Period iv
  4.5 Number of Subjects to be Enrolled iv
  4.6 Endpoints v
    4.6.1 Variables for QT/QTc Interval Analysis v
    4.6.2 Safety Variables v
    4.6.3 Pharmacokinetic Variables v

5. Study Procedures v
  5.1 Screening Procedures v
  5.2 Evaluation of 12-Lead ECG Data for QT/QTc Analysis vi
  5.3 Safety Assessments vii
  5.4 Pharmacokinetic Measurements vii
  5.5 Number of Blood Samplings and Volume of Blood to be Collected viii

List of Abbreviations and Definitions of Terms ix

1. Origin and Course of Development 1
  1.1 Diabetes Mellitus 1
  1.2 Urinary Glucose Reabsorption Mechanism in Renal Proximal Tubules Mediated by Sodium-Dependent Glucose Cotransporter (SGLT) 2
  1.3 Origin and Course of Development 3
  1.4 Rationale for the Planned Study 6

2. GCP Compliance 6

3. Study Administrative Structure 6

4. Study Objectives 6
4.1 Study Objectives 6
4.2 Type of Study 6
5. Study Subjects 6
5.1 Inclusion Criteria 6
5.2 Exclusion Criteria 7
6. Informed Consent and Subject Information Procedures 9
6.1 Preparation of Informed Consent Form and Other Written Information for Subjects 9
6.2 Elements Required for Written Consent Documents 9
6.3 Informed Consent Procedures 10
6.4 Points to Consider When Obtaining Informed Consent 11
6.5 Amendments of Informed Consent Form and Other Written Information (Supply of New Information) 11
7. Registration and Selection of Subjects 12
7.1 Subject Screening Log 12
7.2 Subject Registration List 12
7.3 Selection of Subjects 12
8. Study Drugs 12
8.1 Study Drugs 12
8.1.1 Test Drug 12
8.1.2 Control Drugs 13
8.2 Dosage Form and Contents 13
8.3 Manufacturing of Study Drugs 13
8.4 Packaging and Labeling 14
8.4.1 Packaging 14
8.4.2 Labeling 14
8.5 Storage Conditions 14
8.6 Procedures for Supply, Storage, Management and Retrieval of Study Drugs 14
9. Study Plan 15
9.1 Study Design 15
9.1.1 Design 15
9.1.2 Composition of Each Treatment Group 15
9.1.3 Study Procedure and Period 16
9.2 Dose and Dosage Regimen 17
9.3 Number of Subjects to be Enrolled 18
9.4 Study Period 19
9.5 Evaluation Variables 19

9.5.1 Variables for QT/QTc Interval Analysis 19
9.5.2 Safety Variables 19
9.5.3 Pharmacokinetic Variables 19
10. Study Procedures 19
10.1 Demographics 19
10.2 Diet, Fluid and Activity Control and other Restrictions 20
10.3 Confirmation of Study Drug Indistinguishability, Randomization and Dispensing Drugs 22
10.3.1 Confirmation of Study Drug Indistinguishability 22
10.3.2 Randomization 22
10.3.3 Dispensing Study Drugs 22
10.4 Blinding Procedure and Maintenance of Blinding 23
10.4.1 Treatments to be Blinded 23
10.4.2 Blinding of Study Treatments and Maintenance of Blinding 23
10.4.3 Blinding of Urine Glucose Test 23
10.4.4 Creating and Retaining Emergency Keys 23
10.4.5 Unblinding Procedures 24
10.5 Study Treatments 24
10.6 Schedule of Study Procedures 24
10.7 Screening Procedures 24
10.8 Evaluation of 12-Lead ECG Data for QT/QTc Analysis 25
10.9 Safety Assessments 26
10.10 Assessment of Abnormal Changes 28
10.11 Pharmacokinetic Measurements 28
10.12 Number of Blood Samplings and Volume of Blood to be Collected 29
11. Securing the Safety of Subjects 29
11.1 Basic Principles 29
11.2 Adverse Events 29
11.2.1 Definition of Adverse Events 29
11.2.2 Adverse Event Monitoring 29
11.2.3 Adverse Event Assessments 30
11.2.4 Follow-up of Adverse Events 32
11.3 Serious Adverse Events 33
11.3.1 Definition of Serious Adverse Events 33
11.3.2 Procedures in the Event of a Serious Adverse Event 33
11.3.3 Contact Information in the Event of a Serious Adverse Event 34
11.4 Predictable Adverse Drug Reactions 34
11.4.1 Adverse Drug Reactions Predictable from Nonclinical Studies of TS-071 34
11.4.2 Adverse Drug Reactions Predictable from Clinical Studies of TS-071 34
11.4.3 Measures to be Taken in the Event of Hypoglycemia 35

11.4.4 Adverse Drug Reactions Predictable from Clinical Studies of Avelox® Tablets 35
11.4.5 Measures to be Taken in the Event of Adverse Drug Reaction or Adverse Event Suggestive of TdP 35

12. Criteria and Procedures for Early Termination of a Subject 36
12.1 Criteria for Early Termination 36
12.2 Procedures for Early Termination 37

13. Statistical Analysis 37
13.1 Purpose of Analysis 37
13.2 Analysis Sets 37
13.3 Handling of Cases and Data 38
13.3.1 Handling of Cases 38
13.3.2 Handling of Data 38
13.4 Statistical Analysis Plan 39
13.4.1 Significance Level and Confidence Coefficient 39
13.4.2 Demographics and Other Baseline Characteristics 39
13.4.3 Evaluation Variables and Summary/Analysis Plan 39
13.4.4 Statistical Interpretation of Analysis Results 40
13.4.5 Multiplicity Adjustment 41
13.4.6 Interim Analysis 41
13.5 Fixation of Criteria for Handling of Cases and Data 41
13.6 Fixation of Statistical Analysis Plan 41

14. Protocol Compliance, Deviations or Changes and Amendments 41
14.1 Protocol Compliance and Deviations or Changes 41
14.2 Protocol Amendments 42

15. Study Completion, Premature Termination or Suspension 42
15.1 Study Completion 42
15.2 Premature Termination or Suspension of Study 43
15.2.1 Criteria for Premature Termination or Suspension of Study 43
15.2.2 Procedure for Premature Termination or Suspension of Study 43
15.3 Premature Termination or Suspension of Study at the Study Site 43
15.3.1 Criteria for Premature Termination or Suspension of Study at the Study Site 43
15.3.2 Procedure for Premature Termination or Suspension of Study at the Study Site 43

16. Case Report Forms 44
16.1 Completion and Submission of CRFs 44
16.2 Changes or Amendments of CRF Entries 44
16.3 Guidance for Completion of CRFs 45
16.4 Data Entered in CRFs as Source Data 45

17. Direct Access to Source Documents 45
17.1 Direct Access to Source Documents 45
17.2 Direct Access Procedure 46
18. Quality Control and Quality Assurance for the Study 46
18.1 Quality Control 46
18.2 Quality Assurance 46
19. Ethical Aspects of the Study 46
19.1 IRB Review 46
19.2 Continuing Review 46
19.3 Subject Confidentiality 47
20. Record Retention 47
20.1 Head of the Study Site 47
20.2 IRB Organizer 47
20.3 Principal Investigator 48
20.4 Sponsor 48
21. Payments 48
22. Insurance 48
23. Compensation/Indemnity for Study-Related Health Injury 48
24. Reporting and Publication 49
25. References 49

Appendices and Attachments

Appendix 1: Study Administrative Structure
Appendix 2: Schedule of Study Procedures
Appendix 3: Acceptable Time Windows for Examinations
Attachment: Avelox® tablet 400 mg Package insert

1. Origin and Course of Development

1.1 Diabetes Mellitus

Diabetes mellitus is a group of metabolic disorders that is characterized by chronic hyperglycemia caused by insufficient action of insulin. The number of diabetic patients in Japan is rapidly increasing with changes in lifestyle including dietary habits and insufficient exercise and social environment. According to the Patient Survey performed by the Ministry of Health, Labour and Welfare in 2008, the total number of diabetic patients (estimated number of patients on regular diabetic treatment) was about 2.37 million [(1)]. Furthermore, according to the National Health and Nutrition Survey performed by the Ministry of Health, Labour and Welfare in 2007, the number of people who were strongly suspected to be diabetic was about 8.9 million, and when people who were possibly diabetic were combined, the total number was estimated to be about 22.1 million [(2)]. Diabetes is classified into type 1 and type 2 diabetes by causal factors, and patients with type 2 diabetes account for 90 – 95% of the total number of diabetic patients. Insufficient insulin action is caused by impaired insulin secretion from β-cells in the pancreas and decreased insulin sensitivity (insulin resistance) in the liver and peripheral tissues. In the onset of diabetes, not only genetic factors but also environmental factors including aging, obesity, overeating, insufficient exercise and stress are deeply involved. If chronic hyperglycemia is neglected, microvascular damage and macrovascular damage may develop and cause diabetic complications such as retinopathy, nephropathy, neuropathy, heart diseases and foot gangrene. Further progression of these disorders may result in loss of vision or renal failure requiring dialysis therapy, with a great detriment to the quality of life.

The most important purpose of treatment of diabetes is to prevent onset and progression of these diabetic complications. To date, it has been shown in large-scale clinical trials in patients with type 1 and type 2 diabetes (DCCT, UKPDS, Kumamoto Study) [(3)-(5)] that onset and progression of retinopathy, nephropathy and neuropathy could be prevented by strict blood glucose control. The results of large-scale epidemiologic surveys (DECODE Study, Funagata Study) [(6), (7)] indicated that diabetic patients who had higher blood glucose levels 2 hours after glucose load had a higher relative risk of mortality. This suggests that postprandial hyperglycemia is significantly associated with complications of cardiovascular events. Thus it is desirable to control blood glucose level strictly from the early stage of onset of diabetes.

Basic therapies for diabetes are diet therapy and exercise therapy. If sufficient control of blood glucose cannot be achieved by these therapies, drug therapy is used. Currently, six types of oral antidiabetic drugs and insulin preparations are used in Japan.

- Sulfonylureas stimulate insulin secretion by acting on β-cells in the pancreas and exert a hypoglycemic effect, but these agents have such problems as secondary failure due to long-term use and induction of excessive hypoglycemia.
- Biguanides exert a hypoglycemic effect through their extra-pancreatic activity such as suppression of gluconeogenesis in the liver, inhibition of glucose absorption from the digestive tract and improvement of insulin sensitivity in peripheral tissues. The hypoglycemic effect of this type of agents is mild but they may cause lactate acidosis.

- Alpha-glycosidase inhibitors suppress postprandial hyperglycemia by delaying absorption of glucose from the small intestine. Abdominal fullness and an increase in flatulence have been noted as adverse drug reactions, and especially in elderly patients and patients who have a history of abdominal surgery, caution is necessary for such serious adverse drug reactions as ileus.
- Thiazolidine derivatives exert a hypoglycemic effect by reducing insulin resistance in peripheral tissues and the liver. Caution needs to be exercised for such adverse drug reactions as edema, elevation of serum LDH and an increase in body weight.
- Rapid-acting insulinotropic agents have the same mechanism of action as sulfonylureas and are effective in suppressing postprandial hyperglycemia in a short time after administration, but it is necessary to take caution for the adverse drug reaction of excessive hypoglycemia.
- Dipeptidyl peptidase-IV (DPP-IV) inhibitors exhibit a hypoglycemic effect by promoting activity of incretins, a group of gastrointestinal hormones. Caution should be exercised when they are used concomitantly with sulfonylureas as a risk of serious hypoglycemia may increase.

In addition to these oral antidiabetic drugs, insulin preparations that can be injected subcutaneously are widely used for not only type 1 but also type 2 diabetes. Also, glucagons-like peptide-1 (GLP-1) analogs for subcutaneous injection, which are incretin mimetics, have been marketed.

1.2 Urinary Glucose Reabsorption Mechanism in Renal Proximal Tubules Mediated by Sodium-Dependent Glucose Cotransporter (SGLT)

After being filtrated by glomeruli in the kidney, blood glucose is reabsorbed through the SGLT in renal proximal tubules [(8)]. SGLT2, which has a low affinity and high capacity for glucose, exists in the opening part of the renal proximal tubule where urinary glucose concentration is high, and SGLT1, which has a high affinity and low capacity for glucose, exists in the terminal part of the renal proximal tubule where urinary glucose concentration is low [(9), (10)]. Glucose is almost completely reabsorbed by this two-stage mechanism. In healthy individuals, about 180 g/day of glucose is filtrated by glomeruli in the kidney, and 99% or more of filtrated glucose is reabsorbed in renal proximal tubules and returned to the systemic circulation [(8)]. In a hyperglycemic state, non-absorbable excessive glucose is excreted into urine. Thus glucose reabsorption is limited by the threshold level in the reabsorption process. In renal glucosuria in which blood glucose level is normal while urinary glucose excretion is noted, the threshold level of glucose reabsorption may be assumed to be low, but clinically it is considered not necessary to treat it. It is suggested that the cause of renal glucosuria is related to the deficiency or functional disorder of SGLT2 [(11)].

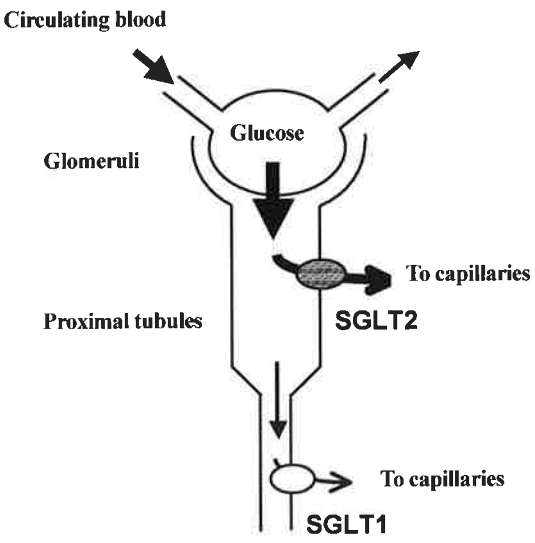

Figure 1-1 Urinary Glucose Reabsorption Mechanism in Renal Proximal Tubules

1.3 Origin and Course of Development

Hyperglycemia is a condition in which an excessively high level of glucose exists in the blood due to absorption of excessive glucose, decreased utilization of absorbed sugar or increased gluconeogenesis in tissues. The drugs currently used for hyperglycemia have such mechanisms of action as delaying glucose absorption, stimulating glucose utilization or suppressing gluconeogenesis. Meanwhile, since blood glucose level can be expected to be normalized in hyperglycemia by eliminating excessive blood glucose from the body, Taisho Pharmaceutical Co., Ltd. embarked on the development of a new type of antidiabetic drug by taking notice of the glucose reabsorption mechanism in renal proximal tubules. Phlorizin, which is a naturally occurring glucose derivative, is known to inhibit SGLT and have an effect of promoting excretion of glucose and lower blood glucose level. However, phlorizin has an *O*-glycoside bond in its molecule, and when it is orally administered, the *O*-glycoside bond is rapidly hydrolyzed by glycosidase in the small intestine and the pharmacological activity is lost. For this reason, the glucose moiety of phlorizin was replaced by thioglucose and the *O*-glycoside bond was changed to metabolically stable C-glycoside bond. By this means, SGL0176, which has an excellent oral absorption property, was created.

SGL0176 (development code: TS-071) is a novel 1-thio-D-glucitol derivative that specifically inhibits the activity of SGLT2 existing in renal proximal tubules and exhibits excellent hypoglycemic action by promoting urinary glucose excretion in various animal models. Since the action of SGL0176 on SGLT2 is highly selective and it normalizes hyperglycemia without stimulating insulin excretion, there are little risks of hypoglycemia and body weight gain. Thus SGL0176 is expected to be effective not only in type 2 diabetes but also in type 1 diabetes. Furthermore, SGL0176 is expected to reduce insulin resistance by avoiding glucotoxicity without placing a burden on β-cells in the pancreas.

In nonclinical studies, the effect of promoting urinary glucose excretion and the hypoglycemic effect were observed in various diabetic animal models. Estimated absorption rates of SGL0176 in rats and dogs were excellent at ≥ 80%, and its main excretion route was fecal excretion. Toxicological findings observed in

common in 26-week repeated dose toxicity study in rats and 52-week repeated dose toxicity study in dogs were diarrhoea or soft stools at dose levels ≥ 100 mg/kg in rats and ≥ 10 mg/kg in dogs, with no observed adverse effect levels being 4 mg/kg in rats and 2 mg/kg in dogs. In embryo-fetal developmental toxicity studies in rats, the incidence of skeletal anomaly increased in fetuses at the dose level of 500 mg/kg. No significant effects were observed in the cardiovascular system.

In the Phase I single-dose study, TS-071 was orally administered in single doses of 1 to 25 mg in healthy Japanese adult male subjects. No serious adverse events were observed, and all adverse drug reactions observed were mild in severity and reversible, suggesting that there would be no problem with tolerability of TS-071. Following administration of TS-071 under fasting conditions, Cmax and AUCinf for SGL0176 in plasma were ascertained to be dose-proportional. In addition, food intake was considered to have little effect on pharmacokinetic parameters. In pharmacodynamics, compared with the placebo group, a statistically significant effect of promoting urinary glucose excretion was noted in all groups receiving TS-071, including the 1 mg dose. This effect was maintained after evening meal at 12 hr post-dose, suggesting the possibility of once-daily administration. The accumulated urinary glucose excretion up to 24 hr post-dose increased with dose. Based on the urinary glucose excretion following each dose and clinical study results of other comparable drugs [(12)-(14)], the upper limit of clinical dose of TS-071 was suggested to be around 5 to 10 mg.
In the Phase I multiple-dose (repeated dose) study, TS-071 was orally administered in a dose of 5 mg or 10 mg for 7 days in healthy Japanese adult male subjects. No serious adverse events were observed. The only adverse drug reaction observed was loose stools in one subject, which were mild in severity and reversible. It was thus considered that there would be no problem with tolerability of TS-071. The plasma concentration of SGL0176 displayed nearly the same time-course change on both Days 1 and 7, with no drug accumulation occurring. In the pharmacodynamic study, compared with the placebo group, statistically significant urinary glucose excretion was maintained in each active drug group from Day 1 to Day 7. Mean urinary glucose excretion/day on Day 7 was similar in the 5 mg and 10 mg groups.
Based on the results of the above Phase I studies and the clinical study results reported with comparable drugs [(12)-(14)], the dose range of 0.5 to 5 mg was selected to explore the dose-response relationship of TS-071, and in a Phase II clinical study (exploratory study), 0.5 mg, 2.5 mg and 5 mg of TS-071 were administered to patients with type 2 diabetes mellitus for 12 weeks. In this study, statistically significant decreases in HbA1c, which was the primary endpoint, and in fasting blood glucose and 2-hr postprandial blood glucose, which were secondary endpoints, were observed in each TS-071 dose group compared with the placebo group. Body weight decreased significantly in the TS-071 groups receiving ≥ 2.5 mg compared with the placebo group; however, the decreases were similar between 2.5 mg and 5 mg groups. Regarding the safety of TS-071, no serious or severe adverse events occurred, the frequency of each event was low, and the incidence of events was not different among the dose groups. TS-071 was thus considered to be well tolerated.
In the clinical pharmacology study (patient PK/PD) conducted in parallel with the above Phase II clinical study (exploratory study), TS-071 was administered to patients with type 2 diabetes in doses of 0.5 mg, 1 mg, 2.5 mg and 5 mg for 7 days. In this study, the time courses of plasma SGL0176 concentrations on Days 1 and 7 were nearly in agreement with each other. In pharmacodynamics, on both Days 1 and 7, statistically significant increases in daily urinary glucose excretion were observed in every dose group compared with the

placebo group. In addition, on Day 7, TS-071 was noted to maintain a statistically significant hypoglycemic effect from breakfast to supper in the groups receiving ≥ 1 mg compared with the placebo group. Regarding safety, no serious adverse event occurred, and only one case of mild and reversible constipation was observed as an adverse drug reaction. TS-071 was considered to pose no particular tolerability problem.

A Phase II clinical pharmacology study (confirmatory study of dose response in patients with type 2 diabetes) was then performed with the doses of 1 mg, 2.5 mg, 5 mg and 10 mg to examine the dose response based on the results of the above-mentioned Phase II study (exploratory study in patients with type 2 diabetes) and clinical pharmacology study (patient PK/PD). In this study, 12-week administration of each TS-071 1 mg, 2.5 mg, 5 mg and 10 mg produced statistically significant decreases in HbA1c, which was the primary endpoint, fasting blood glucose, 2-hr postprandial glucose, glycoalbumin and body weight, which were the secondary endpoints, were observed compared to the placebo group. Of these endpoints, decreases in HbA1c and fasting blood glucose were comparable among the doses of ≥ 2.5 mg, and those in body weight was comparable between 5 mg and 10 mg dose groups. With regard to safety, a total of 3 serious adverse events were experienced by 3 subjects, however, all of them were evaluated as not related to the study medication. None of the observed adverse events was severe in severity. All the adverse drug reactions reported in this study were mild in severity and none of them was severe or moderate. There was no difference in the incidences of adverse events or adverse drug reactions among the dose groups, and no trend was noted in the incidence of each event to increase with the TS-071 dose. TS-071 was thus concluded to be well tolerated.

Furthermore, drug interaction studies (investigation of PK and safety) were performed to investigate the drug interaction in Japanese male subjects when TS-071 was concomitantly with existing oral hypoglycemic agents, i.e., glimepiride, metformin, voglibose, pioglitazone or sitagliptin. The plasma concentration profiles of SGL0176 over time and its pharmacokinetic parameters were almost comparable between administration of TS-071 alone and concomitant administration in each study. In addition, the plasma concentration profiles over time and the pharmacokinetic parameters of the tested hypoglycemic agents were almost comparable between the administration alone and concomitant administration, with an exception of voglibose; blood concentration of unchanged voglibose is not detected due to extremely low gastrointestinal absorption, indicating no effect of concomitant administration on the pharmacokinetics of each other. With respect to safety, there were no serious adverse events or events leading to discontinuation. There were also no findings associated with hypoglycemia following the administration of TS-071 alone or concomitant administration. TS-071 was thus concluded to be well tolerated when concomitantly administered with the existing hypoglycemic agents.

Investigation in patients with type 2 diabetes who show impaired renal function was performed in a clinical pharmacology study with a single dose of TS-071 5 mg. Although the plasma concentration profiles of SGL0176 over time and its pharmacokinetic parameters were affected by decreased renal function, the observed effects on the pharmacokinetics were considered not remarkable. In pharmacodynamics, urinary glucose excretion over 24 hrs after administration increased compared to the day before dosing and the extent of increase decreased with the decline in renal function. As for safety, there were no serious adverse events. Adverse drug reactions including rash and dizziness were observed, however, no effects of the presence or severity of renal impairment on safety were noted. All the adverse events observed in this study were

transient and mild in severity, indicating favorable tolerability.
With regard to 12-lead ECG results in all the clinical studies performed to date, there was only one subject showing a QT prolongation following administration of TS-071 1 mg in the Phase II study (confirmatory study), however, it was mild and did not require any medical intervention.

A phase III clinical study (long-term study) is currently in progress to investigate the safety and efficacy in patients with type 2 diabetes.

1.4 Rationale for the Planned Study

The present study was designed to evaluate the effect of TS-071 on QT/QTc interval in accordance with "The clinical evaluation of QT/QTc interval prolongation and proarrhythmic potential for non-antiarrhythmic drugs (PFSB Notification 1023, No.1 dated October 23, 2009)" (hereinafter referred to as "QT/QTc Guideline").

2. GCP Compliance

This study will be conducted in compliance with the ethical principles set forth in the Declaration of Helsinki, the standards stipulated in Article 14, Paragraph 3, and Article 80-2 of the Pharmaceutical Affairs Law, and the MHW Ordinance No. 28 of March 27, 1997 on the Standards for Conduct of Clinical Trials of Drugs (GCP).
The principal investigator will conduct this study after agreeing with the sponsor on the contents of the protocol and the CRF and compliance therewith.

3. Study Administrative Structure

See "Appendix 1: Study Administrative Structure."

4. Study Objectives

4.1 Study Objectives

The objective of this study is to evaluate the effect of TS-071 on QT/QTc interval in healthy subjects.

4.2 Type of Study

This is a clinical pharmacology study.

5. Study Subjects

Healthy Japanese male and female volunteers who meet the inclusion criteria and do not meet any of the exclusion criteria will be eligible for the study.

5.1 Inclusion Criteria

A subject must meet all of the following criteria:

(1) The subject is aged ≥ 20 and < 45 years at the time of giving informed consent.

(2) The subject's BMI is ≥ 18.5 and < 25.0, and weight is ≥ 40 kg at the screening examination.

(3) The subject's resting pulse rate is ≥ 45 and ≤ 90 beat/min at the screening examination and prior to study drug administration in Period 1.

(4) The subject is eligible for this study as assessed by the principal investigator or subinvestigator based on the screening and pre-treatment examinations up to prior to study drug administration in Period 1*.

(5) The subject is provided with the study procedures prior to participation in the study and is capable of understanding the study and gives his/her own written informed consent.

*: These are subjects who have no abnormalities in the screening as well as pre-treatment examinations up to prior to study drug administration including physical examination, body temperature, blood pressure, pulse rate and 12-lead ECG, and whose clinical laboratory tests at screening and Day -1 (day before study drug administration) in Period 1 are within the reference range of the study site. However, subjects with abnormal findings or values outside the reference range may be enrolled in the study if the findings or values are of no clinical significance and the subjects are eligible as medically assessed by the principal investigator or subinvestigator considering all other related examinations and findings. In that case, the reason for having enrolled such a subject in the study will be given in the case report form.

<Rationale>

(1) The lower age limit of ≥ 20 years is intended to obtain legally acceptable consent for participation in the study; the upper age limit of < 45 years is intended to minimize the effect of decreased metabolic function due to aging.

(2) The range of BMI for adult individuals is intended to minimize the effect of physical frame on the pharmacokinetics in reference to the BMI standards of the World Health Organization (WHO). The lower body weight limit is included because the precaution for use of Avelox® tablets regarding the dose and dosage regimen states that for patients with body weight of < 40 kg the drug should be administered with discretion, e.g., lower dose (200 mg) should be employed.

(3) The limitation of pulse rate is intended to exclude the effect of excessive pulse change on QT/QTc interval.

(4) This is intended to enroll healthy individuals.

(5) This is intended to conduct the study appropriately in compliance with GCP and the Declaration of Helsinki.

5.2 Exclusion Criteria

Any subject who meets any of the following criteria must be excluded:

(1) The subject has hepatic/biliary, respiratory, cardiovascular, gastrointestinal, urological, renal, endocrine or immunological disorder, malignant tumor, diabetes mellitus or impaired glucose tolerance, or has a history thereof within the previous 5 years prior to giving informed consent.

(2) The subject has a congenital disorder or cardiac disorder, or has a history thereof.

(3) The subject has a risk factor for or history of TdP, e.g., heart failure, hypokalemia and a family history

of Long QT Syndrome.

(4) The subject has a history of attack of unconsciousness possibly associated with TdP.

(5) The subject's 12-lead ECG waveform at screening and prior to study drug administration in Period 1 is not suitable for the evaluation of QT/QTc interval prolongation, e.g., drift, myogram, morphology of T wave, remarkable sinus arrhythmia and frequent extrasystoles.

(6) The subject's QTcF in 12-lead ECG at screening or prior to study drug administration in Period 1 is ≥ 450 msec.

(7) The subject falls into any of the following criteria related to renal function:
・Serum creatinine at screening or prior to study drug administration in Period 1 exceeds the upper limit of reference range of the study site;
・Urinary protein at screening and prior to study drug administration in Period 1 is positive (≥ +1);
・Urinary occult blood at screening and prior to study drug administration in Period 1 is positive (≥ +1), with an exception when the positive result is obviously for a non-disease reason such as emmenia.

(8) The subject received an administration of any other study drug within the previous 16 weeks prior to giving informed consent.

(9) The subject previously received an administration of TS-071 tablet (active drug).

(10) The subject donated blood or underwent blood collection as described below:
Donation or collection of blood component within 2 weeks prior to the screening;
Donation or collection of < 400 mL of blood within 4 weeks prior to the screening;
Donation or collection of ≥ 400 mL of blood within 12 weeks prior to the screening for a male subject and within 16 weeks prior to the screening for a female subject

(11) The subject used any medication including an over-the-counter medication within one week prior to study drug administration in Period 1 except lacrimal fluid (eye lotion) for contact lenses.

(12) The subject is unable to abstain from alcohol drinking within 2 days prior to admission to the study site in each study period through discharge, and within 2 days prior to post-treatment examination through its completion.

(13) The subject smokes or smoked within 6 months prior to giving informed consent.

(14) If female, the subject is pregnant, lactating, possibly pregnant, intending to become pregnant during the course of the study, or shows positive pregnancy test at screening or prior to study drug administration in Period 1.

(15) The subject is not capable of using an adequate contraception after giving informed consent through completion of the post-treatment examination.

(16) The subject has a history of allergy to drug or food.

(17) The subject has a significant allergic diathesis, e.g., asthma requiring medical intervention.

(18) The subject is alcohol or drug dependence or a history thereof.

(19) The subject has a positive urine drug screen (phencyclidines, benzodiazepines, cocaines, stimulants, cannabis, morphines, barbiturates and tricyclic antidepressants) at screening.

(20) The subject has a positive result for HBs antigen, HCV antibody, HIV antigen, HIV antibody or serologic test for syphilis at screening.

(21) The subject is, in the principal investigator's or subinvestigator's opinion, unsuitable for any other

reason.

<Rationale>

(1) to (7) , (9) , (12) These are intended to secure the safety of subjects and in consideration of the effect on evaluation. Criteria (2) to (6) are also intended to exclude individuals who show QT/QTc interval prolongation and who have a history or risks of such state in order to make proper evaluation of the effect of the test drug on QT/QTc interval.

(8) This is to secure the safety of subjects and in consideration of the effect on evaluation, and also in reference to the standards stipulated by the Japan Association of Contract Institutes for Clinical Pharmacology (JACIC).

(10) This is to protect the health of subjects because a series of blood collection is required and in reference to the standards for blood donation stipulated by the Japanese Red Cross Society.

(11) This is to secure the safety of subjects and to exclude the effect on evaluation.

(13) Smoking history may possibly affect the evaluation of QT/QTc interval.

(14) , (15) Safety in pregnancy is not established for the treatment of neither TS-071 or Avelox® tablets. In addition, effects of TS-071 exposure via semen on the safety cannot be ruled out at present.

(16) , (17) These are to secure the safety of subjects and to minimize the effect on evaluation.

(18) , (19) It is difficult to manage subjects who are alcohol or drug dependence during the study.

(20) This is to secure the safety of subjects and medical staff, and in consideration of the effect on evaluation.

(21) This is to allow the principal investigator or subinvestigator to judge the appropriateness of enrolling subjects by taking account of unexpected situations.

6. Informed Consent and Subject Information Procedures

6.1 Preparation of Informed Consent Form and Other Written Information for Subjects

The principal investigator, with the cooperation of the sponsor, will prepare an informed consent form and other written information to obtain informed consent from subjects for participation in this study and will revise them when necessary. These documents prepared or revised must be submitted to the sponsor and approved in advance by the Institutional Review Board (IRB).

6.2 Elements Required for Written Consent Documents

Written consent documents must embody at least the following elements:

(1) The study involves research.

(2) The purpose of the study

(3) The name, title and contact address of the principal investigator or subinvestigator

(4) The study procedures (including research aspect of the study, inclusion criteria of subjects and probability of randomization to each treatment group in case of a randomized study)

(5) The expected benefits of the investigational drug for mental and physical health of the subject (when

there is no expected benefit, the subject is to be made aware of this) and foreseeable risks or inconveniences for the subject

(6) The expected duration of the subject participation in the study

(7) The subject's participation in the study is voluntary and the subject may refuse to participate or withdraw from the study at any time without penalty or loss of benefits to which the subject is otherwise entitled.

(8) The monitor(s), auditor(s), the IRB, and the regulatory authorities will be granted direct access to the source documents pertaining to this study; in that case, the confidentiality of the subject will be assured. By signing/sealing a written informed consent form, the subject is authorizing such access.

(9) If the results of the study are published, the subject information will be kept confidential.

(10) The person to contact at the study site for further information regarding the study and the rights of study subjects, and who to inquiry about or contact in the event of study-related injury

(11) The compensation and treatment available to the subject in the event of study-related injury

(12) The approximate number of subjects involved in the study

(13) If information becomes available that may be relevant to the subject's willingness to continue participation in the study, the subject will be informed in a timely manner.

(14) The circumstances and/or reasons under which the subject's participation in the study may be terminated

(15) The anticipated expenses, if any, to the subject for participating in the study

(16) The anticipated payment, if any, to the subject for participating in the study

(17) The subject's responsibilities

(18) Type of the IRB responsible for review and discussion of the appropriateness of this study; other matters to be reviewed and discussed by the IRB; other information about the IRB.

(19) If the subject is receiving medical treatment by a physician other than the investigators of the study, the investigator will inform the physician of the subject's participation in the study with the subject's approval.

6.3 Informed Consent Procedures

Prior to any study procedure for each subject (before screening examination), the principal investigator or subinvestigator will fully explain the study by using the informed consent form and other written information and, after confirming that the subject has fully understood the content of the study, will obtain voluntary written consent from the subject for participation in the study. The consent form will be signed/sealed and personally dated by the subject, the principal investigator or subinvestigator who has explained the study. When a supplementary explanation is given by a clinical research coordinator, the consent form will also be signed/sealed and personally dated by the coordinator.

The principal investigator or subinvestigator will provide each subject giving consent with copies of the signed informed consent form and other written information, and this will be documented. This documentation can

be provided in the medical record or informed consent form when either has a relevant space for such documentation. Original consent forms will be kept in a prescribed place at the study site.

The date when informed consent was obtained will be recorded in the CRF by the principal investigator or subinvestigator.

6.4 Points to Consider When Obtaining Informed Consent

The principal investigator, subinvestigator or clinical research coordinator should take note of the following points when obtaining informed consent from each subject:

(1) No volunteer will be forced to participate and/or continue participation in the study, and no unjustifiable influence will be exerted on the subject's willingness.

(2) The explanation given to the subject should not contain any word or phrase that may lead the subject to waive his/her rights or that may imply that the legal responsibilities of the principal investigator, subinvestigator, clinical research coordinator, the study site and the sponsor involved in this study may be exempted.

(3) The explanation given to the subject should be provided in non-technical language as much as possible which is easily understandable to the subject.

(4) Before informed consent may be obtained, the subject should be given ample time and opportunity to inquire about the study and to decide whether or not to participate in the study. All questions should be answered to the satisfaction of the subject.

6.5 Amendments of Informed Consent Form and Other Written Information (Supply of New Information)

(1) When information becomes available that may be relevant to the subject's willingness to continue participation in the study or new information (e.g., serious adverse drug reaction reporting) becomes available that may affect the subject's consent, the principal investigator or subinvestigator must immediately communicate the information verbally to the subject and confirm whether the subject is willing to continue participation in the study. The information communicated and the subject's response as to whether or not to continue participation will be documented in the medical records or other source documents.

(2) When the principal investigator finds it necessary to revise the informed consent form and other written information based on the information in (1) above, he/she will immediately revise them and obtain the approval of the IRB for the amendments.

(3) The principal investigator or subinvestigator will explain the amendments again by using the revised informed consent form and other written information in (2) above to the subjects including those to whom the information in (1) has been verbally communicated and obtain written consent for continuing participation in the study. However, the subjects who have completed the study may be excluded. Informed consent will be obtained similarly in accordance with the procedure described in "6.3 Informed Consent Procedures."

7. Registration and Selection of Subjects

7.1 Subject Screening Log

The principal investigator will assign a screening number to each prospective subject from whom voluntary written consent has been obtained prior to the study procedure, and enroll them in the screening examination. A subject screening log showing the subjects enrolled in the screening examination will be created and retained, and a duplicate of the log will be provided to the sponsor. If a subject is not registered in the study, the reason thereof will be documented in the log.

To identify each subject, a screening number will be employed; his/her initials or medical record number should not be used.

7.2 Subject Registration List

The principal investigator will assign a subject identification code to each subject to receive administration of study drug among those who gave voluntary written consent and underwent the screening examination, and create and retain a subject registration list showing subjects to receive study drug.

Date of giving informed consent and subject identification code for each subject will be listed in the subject registration list.

7.3 Selection of Subjects

Final selection of subjects to receive administration of study drug will occur on the day of study drug administration (Day 1) of Period 1. The principal investigator will select eligible subjects, from ethical and scientific standpoints, based on the inclusion and exclusion criteria to satisfy the purpose of study. In selection of the subjects, the followings will be considered: the subject's health condition, age, gender, capability of giving consent, relationship to or dependency on the principal investigator or subinvestigator, any participation in other clinical trials, screening results and observation/examination results prior to study drug administration in Period 1 (for clinical laboratory tests, results up to the Day -1 in Period 1 will be reviewed). Results of observation/examinations up to the final subject selection will be documented in the CRF.

A subject who has been finally selected may be replaced by a reserve subject before starting study drug administration in Period 1 when he/she has to be withdrawn for some reason. Reserve subjects who have not replaced will be discharged from the study site with his/her agreement without receiving study drug administration. Those reserve subjects who have not received study drug administration may be enrolled in the study of another admission group.

8. Study Drugs

8.1 Study Drugs

8.1.1 Test Drug

Development code: TS-071 (Drug substance identification code: SGL0176 hydrate)

Chemical name: (1*S*)-1,5-Anhydro-1-[5-(4-ethoxybenzyl)-2-methoxy-4-methylphenyl]-1-thio-D-glucitol hydrate

Structural formula:

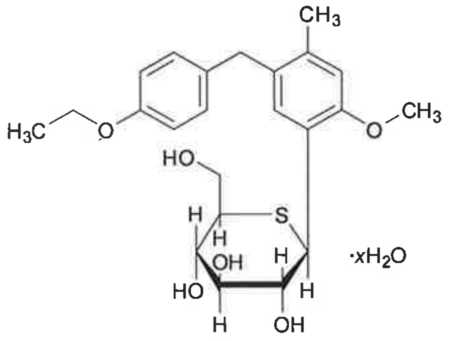

Molecular formula: $C_{23}H_{30}O_6S \cdot xH_2O$

8.1.2 Control Drugs

(1) TS-071 tablets placebo

(2) Avelox® tablets (positive control)

Generic name: moxifloxacin hydrochloride (JAN)

Chemical name: 1-Cyclopropyl-6-fluoro-8-methoxy-7-[(4a*S*,7a*S*)-octahydropyrrolo[3,4-*b*]pyridin-6-yl]-4-oxo-1,4-dihydroquinoline-3-carboxylic acid monohydrochloride

8.2 Dosage Form and Contents

"Table 8-1 Dosage Form and Contents of Study Drugs" shows the dosage form and contents of each study drug.

Table 8-1 Dosage Form and Contents of Study Drugs

Study drug		Dosage form and contents
Test drug	TS-071 tablets 5 mg	White to grayish white film-coated tablets containing 5 mg of SGL0176 per tablet
Control drug	TS-071 tablets placebo	White to grayish white film-coated tablets containing no SGL0176 or other medicinal properties that are identical in appearance to TS-071 tablets 5 mg
Control drug (positive control)	Avelox® tablets	Pale reddish gray film-coated tablets with a cleavage containing 400 mg of moxifloxacin (436.8 mg as moxifloxacin hydrochloride) per tablet

8.3 Manufacturing of Study Drugs

Taisho Pharmaceutical Co., Ltd. will manufacture the study drugs (TS-071 tablets) and package the commercially available Avelox® tablets (manufactured by Bayer Yakuhin, Ltd.) as study drugs.

8.4 Packaging and Labeling

8.4.1 Packaging

TS-071 tablets 5 mg and TS-071 tablets placebo will be provided in plastic bottles containing 50 tablets each. Avelox® tablets will be provided in press through packages (PTPs) containing 5 tablets each. Ten PTP sheets will be packaged in a transparent bag, which will further be packaged in a paper box.

8.4.2 Labeling

The study drugs will carry the following label.

<For clinical trial use><br>Name of study drug<br><br>Lot No. Storage condition: at room temperature<br>Taisho Pharmaceutical Co., Ltd.<br><br>Clinical Research<br>24-1, Takada 3-chome, Toshima-ku, Tokyo<br>TEL: 03-3985-1306

Figure 8-1 Sample Label on Lateral Side of Bottle or Box

Table 8-2 Name of Study Drug and Lot Number Displayed on the Label

Name of Study Drug	Lot No.
TS-071 tablets 5 mg	[redacted]
TS-071 tablets placebo	[redacted]
Avelox® tablets 400 mg	[redacted]

8.5 Storage Conditions

All the study drugs should be stored at room temperature. Refer to the "Procedure for study drug management" for the expiration dates.

8.6 Procedures for Supply, Storage, Management and Retrieval of Study Drugs

The sponsor will prepare "Procedure for study drug management", which will detail the procedures for the receipt, handling, storage, management, prescription and dispensing of study drug as well as procedures for return of unused drugs to the sponsor.

In accordance with this written procedure provided by the sponsor, the study drug manager at the study site will store and manage the study drugs appropriately.

9. Study Plan

9.1 Study Design

9.1.1 Design

This is a randomized, single dose, 4-period crossover study based on a Williams design consisting of 4 treatment sequences.

Each treatment sequence comprises of 4 treatment groups, i.e., anticipated therapeutic dose, supratherapeutic dose, placebo and Avelox.

- Placebo and Avelox (positive control) treatment groups are included as controls.
- Treatments of anticipated therapeutic dose, supratherapeutic dose and placebo will be double-blinded.
- Treatment of Avelox will not be blinded.
- 12-lead ECG data for QT/QTc analysis will be blinded for all the treatments to the ECG readers at the central ECG analysis facility.

<Rationale>

- Four treatment groups, i.e., anticipated therapeutic dose, supratherapeutic dose, placebo and Avelox, were selected in accordance with the QT/QTc Guideline.
- A crossover design can minimize the inter-individual variability by using a self-controlled method and enhance analytical precision.
- When TS-071 5 mg and 10 mg were administered once daily for 7 days in the multiple-dose study, there was no accumulation of plasma unchanged TS-071 or the metabolites (M-2 and M-17), and the mean elimination half-life ($T_{1/2}$) following the single doses of 5 mg, 15 mg and 25 mg was 9.72 hr, 13.8 hr and 12.6 hr, respectively. Based on these results, single administration of TS-071 will allow us to evaluate the effect on QT/QTc interval.
- Moxifloxacin (Avelox® tablets) will be used as a positive control to confirm the assay sensitivity for QT/QTc interval because it is known to prolong QT/QTc interval and generally employed as a positive control in thorough QT/QTc studies.
- A double-blind study for the anticipated therapeutic dose, supratherapeutic dose and placebo treatments can eliminate bias on evaluation.
- Evaluation of 12-lead ECG data for QT/QTc analysis of all treatment groups will be blinded. Unblinded treatment of Avelox can be justified because the evaluation variables will be measured and all treatment groups will be tested equally in a well-controlled study.

9.1.2 Composition of Each Treatment Group

Composition of each treatment group is as follows:

Anticipated therapeutic dose: Subjects will receive one tablet of TS-071 tablet 5 mg and 3 tablets of TS-071 tablet placebo.

Supratherapeutic dose: Subjects will receive 4 tablets of TS-071 tablet 5 mg.

Placebo: Subjects will receive 4 tablets of TS-071 tablet placebo.

Avelox group: Subjects will receive one tablet of Avelox® tablet 400 mg.

<Rationale>

There will be 2 treatment groups for the test drug, i.e., anticipated therapeutic dose and supratherapeutic dose in accordance with the QT/QTc Guideline. As controls, placebo and Avelox (positive control) treatments will be included.

9.1.3 Study Procedure and Period

The study will be performed according to "Figure 9-1 Study Flowchart." The study period for each subject is defined as "from the date of giving informed consent to the date of post-treatment examination (or date of early termination)."

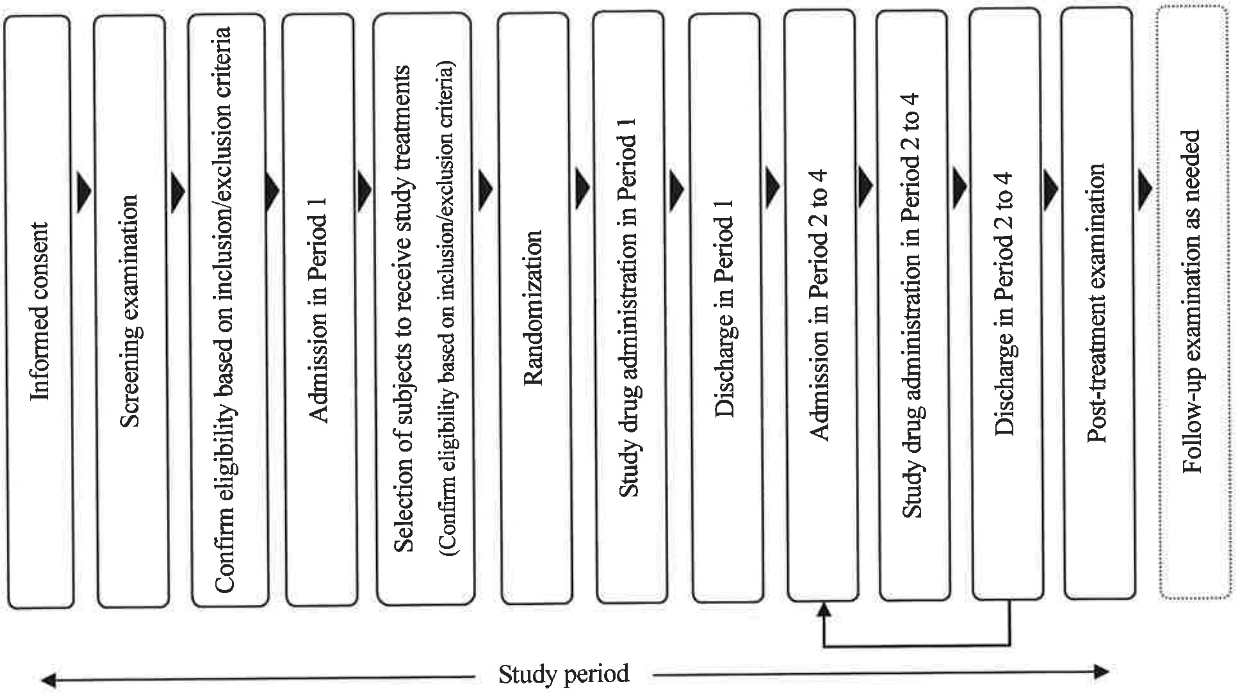

Figure 9-1 Study Flowchart

Duration of hospitalization will be 4 days and 3 nights.

Post-treatment examination will be conducted with an interval of ≥ 7 days after study drug administration in Period 4 (on Day 8 or thereafter when Day 1 is the day of study drug administration in Period 4).

<Rationale>

Duration of hospitalization will be 4 days and 3 nights considering the followings:

- Based on the time to maximum plasma concentration (Tmax) and $T_{1/2}$ data of each study drug observed in the previous clinical studies, safety and pharmacokinetic variables need to be monitored up to 48 hours after administration.

・Subjects need to be admitted to the study on Day -1 to compare the status before and after study drug administration.

The timing of post-treatment examination, ≥ 7 days after the final study drug administration, was selected to have an interval ≥ 5 times of $T_{1/2}$ in reference to "Guideline for Bioequivalence Studies of Generic Products" [15] and also considering the study feasibility.

Mean Tmax values following single oral dose in healthy adult males are:

TS-071 5 mg : 1.06 hr
TS-071 15 mg : 1.56 hr
TS-071 25 mg : 2.25 hr
moxifloxacin 400 mg : 1.75 hr

Mean $T_{1/2}$ values following single oral administration in healthy adult males are:

TS-071 5 mg : 9.72 hr
TS-071 15 mg : 13.8 hr
TS-071 25 mg : 12.6 hr
moxifloxacin 400 mg : 13.9 hr

(See "Investigator's Brochure of TS-071 (Version 6)" and "Attachment: Avelox® tablet 400 mg Package insert" for the Tmax and $T_{1/2}$ values.)

9.2 Dose and Dosage Regimen

Dose of each treatment group will be as follows:

Anticipated therapeutic dose: TS-071 5 mg
Supratherapeutic dose: TS-071 20 mg
Placebo: TS-071 placebo
Avelox: moxifloxacin 400 mg

Subjects will receive a single oral dose of a study drug assigned in each study period with 200 mL of water in the fasted state in the morning of study drug administration day according to the randomization code. There will be a wash-out interval of ≥ 7 days between each study period, i.e., when the preceding dose is on Day 1, the subsequent dose will be on Day 8 or thereafter.

<Rationale for the doses>

The doses for each treatment group, i.e., anticipated therapeutic dose, supratherapeutic dose, placebo and Avelox were determined based on the following results and assumptions:

・Anticipated therapeutic dose (TS-071 5 mg):

In the Phase II exploratory and confirmatory studies, statistically significant decreases in the change in HbA1c, which is the primary efficacy endpoint, and in the change in fasting blood glucose, which was

the secondary endpoint, were observed in all TS-071 dose levels (0.5 to 10 mg) compared to the placebo, whereas the extent of decreases was similar in the dose levels of ≥ 2.5 mg. Of the secondary endpoints tested, the changes in 2-hr postprandial blood glucose were larger in the groups receiving ≥ 5 mg compared to 2.5 mg in the Phase II exploratory study, and those in body weight were larger in the groups receiving ≥ 5 mg compared to 2.5 mg in the Phase II confirmatory study. With respect to safety, TS-071 was demonstrated to be well tolerated up to the dose of 10 mg.

The anticipated therapeutic dose is considered to be 2.5 mg, and the dose will be increased to 5 mg if sufficient effects are not demonstrated. The anticipated therapeutic dose for this study was thus determined to be 5 mg, which is the highest therapeutic dose of TS-071.

- Supratherapeutic dose (TS-071 20 mg):

  The observed Cmax of unchanged TS-071 (299 ng/mL on Day 7) following 7-day repeated administration of TS-071 5 mg in the clinical pharmacology study (patient PK/PD) in patients with type 2 diabetes is regarded as the maximum exposure anticipated in the therapeutic use. The dose of 20 mg, which is expected to show substantially larger exposure, was selected as the supratherapeutic dose according to the QT/QTc Guideline. Based on the Phase 1 single dose study in Japanese healthy male subjects, the TS-071 dose range of 1 to 25 mg will pose no particular tolerability problem.

- Avelox:

  In the thorough QT/QTc studies conducted in foreign countries Avelox 400 mg was generally employed as a positive control, which is also the approved daily dose in Japan.

<Rationale for the dosage regimen>

The amount of water to be taken with the study drug was determined according to "Guideline for Bioequivalence Studies of Generic Products" (15).

The interval between treatments of ≥ 7 days was selected to have an interval ≥ 5 times of $T_{1/2}$ (see "9.1.3 Study Procedure and Period") in reference to "Guideline for Bioequivalence Studies of Generic Products" (15) and also considering the feasibility of the study.

9.3 Number of Subjects to be Enrolled

Fifty-six (56)

The number of male and female subjects in each treatment sequence should be as equal as possible. There will be 2 or more reserve subjects per admission group to stand by for study drug administration in Period 1, who will replace any withdrawn subjects prior to administration.

<Rationale>

The sample size was selected in reference to thorough QT/QTc studies carried overseas with a crossover design similarly to the present study (16), (17) since the results of thorough QT/QTc studies in Japanese subjects have not been made public to date.

9.4 Study Period

From June 13, 2011 to October 31, 2011

9.5 Evaluation Variables

9.5.1 Variables for QT/QTc Interval Analysis

The primary endpoint is QTcF, QT interval corrected by Fridericia's formula, and the secondary endpoint is QTcB, QT interval corrected by Bazett's formula.

<Rationale>
Fridericia's and Bazett's formulae will be used to correct QT interval according to the QT/QTc Guideline.

9.5.2 Safety Variables

(1) Adverse events and adverse drug reactions, including abnormal changes in body temperature, blood pressure, pulse rate, 12-lead ECG and clinical laboratory tests
(2) Effects on body temperature, blood pressure, pulse rate, 12-lead ECG and clinical laboratory tests

<Rationale>
These measurements were selected to investigate the effect on the subjects and confirm the safety of subjects.

9.5.3 Pharmacokinetic Variables

Plasma concentrations of unchanged TS-071 and its metabolites (M-2 and M-17), and plasma concentrations of unchanged moxifloxacin

<Rationale>
Plasma concentrations of unchanged TS-071 and unchanged moxifloxacin will be determined to investigate the relationship of their single-dose pharmacokinetics to QT/QTc interval in accordance with the QT/QTc Guideline, and also to confirm the pharmacokinetic parameters of TS-071 and moxifloxacin including Cmax, Tmax, AUC and $T_{1/2}$.

10. Study Procedures

10.1 Demographics

The principal investigator or subinvestigator will capture the following subject's demographic information prior to study drug administration in Period 1 and document it in the CRF.

(1) Date of birth
(2) Ethnicity (Confirm that the subject is Japanese)
(3) Gender
(4) Present illness and its history that is ongoing at the time of study drug administration in Period 1
(5) Medical history within the previous 5 years prior to informed consent; information on medical

history relevant to the exclusion criteria (2) to (4) before that period should also be captured.

(6) Family history, i.e., present illness and its history and past medical history relevant to the risk factors for TdP

(7) Drinking and smoking status

(8) Height, weight and BMI at screening

(9) Use of medications within one week prior to study drug administration in Period 1, including over-the-counter medications

10.2 Diet, Fluid and Activity Control and other Restrictions

Subjects will stay under supervision of the principal investigator or subinvestigator and will be instructed to adhere to the following instructions. The principal investigator or subinvestigator will check the adherence status at the time of physical examination.

Subjects will be discharged from the study site after the principal investigator or subinvestigator confirms that they have no abnormality in their healthy conditions as determined by the safety data up to 48 hr post-dose in each study period including physical examination, body temperature, blood pressure, pulse rate, 12-lead ECG and clinical laboratory tests.

When a prolongation of hospitalization, re-examination or additional examination is required for a subject at the discretion of the principal investigator or subinvestigator, he/she will prolong the hospitalization or perform unscheduled examination to confirm the subject's safety after explaining the reason and obtaining the subject's approval until there are no health concerns. Such unscheduled examinations or prolonged hospitalization will be documented in the CRF.

<Instructions to subjects and other study restrictions>

(1) Subjects will rest in the supine position on the bed for 10 min (5 min at screening) prior to and during 12-lead ECG recording.

(2) Beverages other than those provided at the study site will not be allowed during hospitalization. Consumption of any beverages will be prohibited within 2 hours before and 3 hours after study drug administration except water for dosing.

Consumption of caffeinated beverages and foods (e.g., coffee, red tea, oolong tea, green tea, cola, cocoa and chocolate), alcoholic beverages and those containing grapefruit will be prohibited within 2 days before admission to the study site through discharge in each study period and within 2 days before post-treatment examination through its completion. Excessive consumption of such beverages and foods will not be allowed including in the periods other than those described above.

(3) Foods other than those provided at the study site will not be allowed during hospitalization.

Consumption of any food will be prohibited within 10 hours before screening examination, examination on Day -1 and post-treatment examination.

Subjects will start fasting 10 hours before study drug administration and keep fasting until 4 hours after administration. Contents of meals during hospitalization (from supper on Day -1 through breakfast at 48 hr post-dose) will be consistent for the same subject and between subjects from Period 1 through Period 4, however, number of calories will be different for male and female subjects. Time of starting meals (supper on Day -1, lunch on Day 1, breakfast at 24 hr post-dose

and breakfast at 48 hr post-dose in each period) will be documented in the CRF.

(4) Smoking will be prohibited after the time of informed consent through completion of post-treatment examination.

(5) Strenuous physical exercise will be prohibited within 2 days before admission to the study site through discharge in each study period and within 2 days before post-treatment examination through its completion.

(6) Use of medications other than the study drug including over-the-counter medications will not be allowed within one week before study drug administration in Period 1 through completion of post-treatment examination, except lacrimal fluid (eye lotion) for contact lenses. Medications may be used at the discretion of the principal investigator or subinvestigator when they are needed to secure the safety of the subject, e.g., to treat an adverse event. Use of other medications will be documented in the CRF according to "11.2.3 Adverse Event Assessments (6) Medical treatment for adverse event" when they are used to treat an adverse event; when they are used for the purpose other than an adverse event, name of medication, dosage regimen, period of administration (start and end dates) and purpose of use will be documented in the CRF.

(7) Subjects will be instructed to use an adequate contraception after giving informed consent through completion of post-treatment examination.

(8) Blood collection or donation for the purpose other than this study will be prohibited after giving informed consent through completion of post-treatment examination.

(9) Urine glucose test (qualitative or quantitative) will not be allowed after study drug administration in Period 1 through 3 days after the final administration.

<Rationale>

(1) This is intended to minimize the effect of excessive pulse change on 12-lead ECG.

(2) Fluid consumption is likely to affect pharmacokinetics. The duration to prohibit fluid intake was determined considering the Tmax values of TS-071 after single administration of 5 mg, 15 mg and 25 mg (1.06 hr, 1.56 hr and 2.25 hr, respectively) and that of Avelox® tablets after single administration (1.75 hr). Caffeine and alcohol are likely to impact on diuresis, hepatic and renal drug metabolism, QT/QTc interval and transaminase values. Grapefruit-containing foods and beverages are likely to affect pharmacokinetics.

(3) These restrictions are intended to exclude the effect on variables for evaluation and also in reference to "Guideline for Bioequivalence Studies of Generic Products" [(15)].

(4) Smoking is likely to affect gastrointestinal motility, drug absorption and QT/QTc interval.

(5) Escape enzymes from musculoskeletal muscle caused by strenuous exercise, such as AST (GOT) and CK (CPK), are likely to confound the safety evaluation and affect QT/QTc interval.

(6) Use of other medications may confound the protocol-directed evaluation. The principal investigator and subinvestigator will be allowed to prescribe concomitant medications at his/her own discretion to assure the safety of subjects.

(7) Safety in pregnancy is not established for the treatment of either TS-071 or Avelox® tablets. In addition, effects of TS-071 exposure via semen on the safety cannot be excluded at present.

(8) This is intended to assure the safety of subjects.
(9) This is intended to maintain blinding of TS-071 treatments.

10.3 Confirmation of Study Drug Indistinguishability, Randomization and Dispensing Drugs

Confirmation of study drug indistinguishability, randomization and dispensing drugs will follow the procedures below. Further details will be shown in a written procedure to be prepared by the random assignment manager.

10.3.1 Confirmation of Study Drug Indistinguishability

The random assignment manager will verify that the study drugs (TS-071) are identical in appearance in terms of their color, shape, size and smell prior to study drug administration of the first subject in Period 1 and at the time of retrieving study drugs to the sponsor to make sure that the blinding of study drugs is maintained. The random assignment manager will create 2 sets of documents verifying the maintenance of blinding after all the relevant procedures, retain one of the documents and provide the other to the sponsor.

10.3.2 Randomization

10.3.2.1 Assigning Treatment Sequences

The random assignment manager will randomly assign treatment groups to determine the order of treatments within each treatment sequence by the time of study drug administration to the first admission group in Period 1 according to the predetermined procedure. A randomization code will be created for each treatment sequence, sealed and securely retained until unblinding. The randomization codes of treatment sequences will be provided to the sponsor after unblinding. Prior to sealing the randomization codes, the random assignment manager will create randomization codes of study treatments for each admission group, and then seal the randomization codes of treatment sequences according to the procedure described in "10.3.2.2 Assigning Study Treatments."

10.3.2.2 Assigning Study Treatments

The random allocation manager will randomly assign study treatments according to the predetermined procedure. The random allocation manager will create randomization codes of study treatments that list subject identification codes and corresponding treatment sequences according to the randomization codes of treatment sequences, and seal the original codes and their duplicates separately. The randomization codes of study treatments may be unblinded by the random allocation manager according to the procedure described in "10.3.3 Dispensing Study Drugs." The randomization codes of study treatments will be securely retained by the random allocation manager and their duplicates will be provided to the drug assay laboratories.

10.3.3 Dispensing Study Drugs

The random assignment manager will dispense the study drugs and document the dispensing according to the "Procedure for study drug management."
The study drugs will be dispensed for each admission group prior to study drug administration in each study period. The randomization codes of study treatments will be unblinded every time the study drugs are

dispensed and sealed again after dispensing.

10.3.4 Assigning Study Treatments to Subjects

On Day 1 of Period 1, the principal investigator will allocate a subject identification code to each subject who is to receive study drug administration (see "7.2 Subject Registration List") and administer the dispensed study drug to each subject. When a subject who is to receive study drug administration is replaced by a reserve subject, the replacing subject will receive the study drug that is planned to be administered before replacement.

10.4 Blinding Procedure and Maintenance of Blinding

The procedures for blinding and maintaining blinding are described below. Further details will be shown in a written procedure to be prepared by the random assignment manager.

10.4.1 Treatments to be Blinded

Treatments of anticipated therapeutic dose, supratherapeutic dose and placebo will be double-blinded, and treatment of Avelox will not be blinded. 12-lead ECG data for QT/QTc analysis for all the treatments will be read by the blinded ECG readers at the central ECG analysis facility.

10.4.2 Blinding of Study Treatments and Maintenance of Blinding

The random allocation manager will securely retain the randomization codes of study treatments until unblinding, and should not disclose the allocated treatments to any party until unblinding, except when unblinding emergency keys.

The responsible person at the drug assay laboratories will securely retain the duplicates of the randomization codes of study treatments and unblind them prior to drug assay. The responsible person should not disclose the allocated treatments to any third party including the sponsor, central ECG analysis facility, study site and the drug assay consigner prior to unblinding.

The treatment of Avelox will not be blinded, however, the ECG readers at the central ECG analysis facility will be blinded to all the treatments. Appropriate measures should be taken to maintain the blinding of ECG data, e.g., shielding the time of recording 12-lead ECG charts and subject identification information in accordance with the procedure of the facility.

10.4.3 Blinding of Urine Glucose Test

Urine glucose (qualitative or quantitative) should not be tested to maintain blinding after study drug administration in Period 1 through 3 days after the final administration.

10.4.4 Creating and Retaining Emergency Keys

The random allocation manager will create 2 sets (original and duplicate) of emergency keys and seal them separately. The original keys will be retained by the sponsor and the duplicates by the random allocation manager. The emergency keys will only be unblinded in accordance with the predetermined procedure (see "10.4.5.2 Unblinding Emergency Keys" for unblinding.)

Emergency keys will be created and sealed separately for each subject in each study period so that the study treatment of a relevant subject in a relevant study period can be unblinded.

Prior to the unblinding procedures, the random allocation manager will verify that the original and duplicate emergency keys are sealed and provide both original and duplicate keys to the sponsor after unblinding. When an emergency key has been unblinded, the random assignment manager will verify that the emergency keys other than those unblinded are sealed.

10.4.5 Unblinding Procedures

10.4.5.1 Unblinding Randomization Codes

The random allocation manager will unblind the randomization codes of treatment sequences and those of study treatments after all the CRF data are entered, safety and QT/QTc analysis data including their handling are fixed, and the randomization codes are confirmed to be sealed. After unblinding, the randomization codes of treatment sequences and those of study treatments will be provided to the sponsor.

10.4.5.2 Unblinding Emergency Keys

When a serious adverse event occurs and the principal investigator or subinvestigator finds it necessary to unblind the subject's emergency key to secure the safety, he/she will contact the sponsor to unblind the subject's emergency key of the relevant study period and document in the CRF the information including the subject identification code, relevant study period, reason for unblinding and safety data. When the sponsor finds it necessary to unblind the emergency key, the sponsor monitor will confirm that the above information is entered in the CRF, unblind the emergency key without delay, and inform the principal investigator or subinvestigator of the allocated treatment. The principal investigator will record the unblinding procedure of the emergency key upon consultation with the sponsor and provide the sponsor with the record. The sponsor will report the allocated treatment and the reason for unblinding to the medical expert and the random allocation manager. The handling of the relevant subject's data will be determined by the sponsor.

10.5 Study Treatments

Study drug administration to each subject will be witnessed by the principal investigator or subinvestigator. The date and time of each administration will be documented in the CRF.

10.6 Schedule of Study Procedures

The principal investigator or subinvestigator will perform the study in accordance with the scheduled as provided in "Appendix 2: Schedule of Study Procedures". The dates of screening and post-treatment examination will be documented in the CRF.

10.7 Screening Procedures

The principal investigator or subinvestigator will obtain written informed consent from candidate subjects and perform the screening examination as described in "Table 10-1 Screening Examination" within one day (day of admission) to 28 days prior to study drug administration in Period 1 to select eligible subjects for the study. Results of screening examination will be documented or attached in the CRF. Physical examination data to be documented in the CRF consist of medical history, present illness and its history, family history, and

drinking and smoking status. 12-lead ECG results will include normal/abnormal assessment of the finding. ECG finding will be interpreted either as "within normal limits" or "clinically significant abnormal finding", and any "clinically significant abnormal finding" should be detailed in the CRF.

Height, weight, body temperature, blood pressure and pulse rate will be measured and blood and urine samples for clinical laboratory tests and other procedure will be collected, measured and stored in accordance with the procedures of the study site.

When a re-examination or additional examination is required for a subject at the discretion of the principal investigator or subinvestigator, he/she will perform such unscheduled examination to confirm the subject's safety after explaining the reason and obtaining the subject's approval, and document or attach the record in the CRF.

Table 10-1 Screening Examination

Physical examination		Subjective symptoms, objective findings (inspection, auscultation, percussion, palpation) and medical interview (medical history, present illness and its history, family history, drug treatment history, allergy history, previous clinical study participation, previous blood donation/collection, drinking status, smoking status, other)
Height, weight, body temperature, blood pressure, pulse rate		Height, weight, (BMI*1), body temperature*2, blood pressure*3, pulse rate*3
ECG		12-lead ECG*4 (RR, PR, QRS, QT, QTcF)
Clinical laboratory tests	Hematology tests	White blood cell count, red blood cell count, hemoglobin, hematocrit, platelet count, WBC differentials (neutrophils, lymphocytes, monocytes, eosinophils, basophils)
	Blood chemistry tests	Total bilirubin, direct bilirubin, AST(GOT), ALT(GPT), AL-P, LDH, γ-GTP, CK(CPK), total protein, albumin, A/G ratio, BUN, uric acid, creatinine, cystatin C, total cholesterol, LDL cholesterol, HDL cholesterol, triglycerides, glucose, Ca, P, Na, K, Cl, Mg, CRP
	Urinalysis	Qualitative tests (urobilinogen, ketones, glucose, protein, occult blood, pH), sediment
Immunology tests		HBs antigen, HCV antibody, HIV antigen, HIV antibody, serologic test for syphilis
Urine drug screen		Phencyclidines, benzodiazepines, cocaines, stimulants, cannabis, morphines, barbiturates, tricyclic antidepressants
Pregnancy status (female only)		Pregnancy test

*1: BMI will be calculated as Weight (kg) / Height $(m)^2$ and rounded to one decimal place.
*2: Body temperature will be measured under armpit.
*3: Blood pressure and pulse rate will be measured at rest in the supine position.
*4: 12-lead ECG will be recorded in the supine position after resting for 5 min.

10.8 Evaluation of 12-Lead ECG Data for QT/QTc Analysis

12-lead ECG for QT/QTc analysis will be recorded according to a separate written procedure and recordings will be read at the central ECG analysis facility. The same model of 12-lead digital surface ECG monitor should be employed for all the subjects at all the time points.

Three charts of 12-lead ECG for QT/QTc analysis will be recorded in 5 min for each time point as shown in

"Table 10-2 Time Points for 12-Lead ECG for QT/QTc Analysis." The interval between the start of each recording should be ≥ 1 min, and ECG will be recorded for 10 seconds for each chart. The acceptable time windows for recording ECG are given in "Appendix 3: Acceptable Time Windows for Examinations." All 12-lead ECG charts for QT/QTc analysis will be sent electronically by the responsible person of the study site to the central ECG analysis facility. The time of recording 12-lead ECG for QT/QTc analysis (3 charts to be measured by the central ECG analysis facility) will be documented in the CRF. For the time points when 12-lead ECG are recorded for safety evaluation only, the time of recording 12-lead ECG for safety evaluation except post-treatment examination will be documented in the CRF.

For all the ECG charts for QT/QTc analysis sent to the central ECG analysis facility, RR, QT, PR and QRS intervals will be measured in accordance with the procedure of the facility. The time of recording and subject identification information should be shielded before measurement. QTc intervals will be corrected by Fridericia's and Bazett's formulae.

The effect of TS-071 on QT/QTc interval prolongation will be regarded negative when the upper bound of the 95% one-sided confidence interval for the largest mean effect on the QT/QTc interval excludes 10 msec at all the time points in the TS-071 groups.

In addition, pharmacokinetic parameters will be determined from plasma concentrations of unchanged TS-071 and moxifloxacin, and an exploratory investigation will be made to assess the relationship of drug concentrations to changes in QT/QTc interval.

Table 10-2 Time Points for 12-Lead ECG for QT/QTc Analysis

Variable	Time after study drug administration
12-lead ECG for QT/QTc analysis*	Pre-dose and 0.5, 1, 2, 3, 4, 6, 8, 24 hr post-dose in each study period

*: 12-lead ECG will be recorded in the supine position after resting for 10 min.

<Rationale>

A standard 12-lead digital surface ECG monitor will be used to measure QT/QTc intervals with accuracy and precision. The central ECG analysis facility will be responsible for ECG readings because intervals of 12-lead ECG waveforms for QT/QTc analysis need to be read by skilled readers.

The time points that will enable a proper evaluation of the effect of study drugs on QT/QTc interval were selected considering the observed Tmax values following single doses of TS-071 5 mg, 15 mg, 25 mg and moxifloxacin 400 mg (1.06 hr, 1.56 hr, 2.25 hr and 1.75 hr, respectively).

10.9 Safety Assessments

The principal investigator or subinvestigator will conduct the observations/examinations described in "Table 10-3 Variables for Safety Assessment" in accordance with the schedule provided in "Appendix 2: Schedule of Study Procedures." The acceptable time window for each examination is given in "Appendix 3: Acceptable Time Windows for Examinations." Body temperature, blood pressure and pulse rate will be measured and blood and urine samples for clinical laboratory tests and other procedure will be collected, measured and stored in accordance with the procedures of the study site.

Table 10-3 Variables for Safety Assessment

Physical examination		Subjective symptoms, objective findings (inspection, auscultation, percussion, palpation) and medical interview
Body temperature, blood pressure, pulse rate		Body temperature*[1], blood pressure*[2], pulse rate*[2]
ECG		12-lead ECG*[3]
Clinical laboratory tests	Hematology tests	White blood cell count, red blood cell count, hemoglobin, hematocrit, platelet count, WBC differentials (neutrophils, lymphocytes, monocytes, eosinophils, basophils)
	Blood chemistry tests	Total bilirubin, direct bilirubin, AST(GOT), ALT(GPT), AL-P, LDH, γ-GTP, CK(CPK), total protein, albumin, A/G ratio, BUN, uric acid, creatinine, cystatin C, total cholesterol, LDL cholesterol, HDL cholesterol, triglycerides, glucose, Ca, P, Na, K, Cl, Mg
	Urinalysis	Qualitative tests (urobilinogen, ketones, glucose, protein, occult blood, pH), sediment
Pregnancy status (female only)		Pregnancy test

*1: Body temperature will be measured under armpit.
*2: Blood pressure and pulse rate will be measured at rest in the supine position.
*3: 12-lead ECG will be recorded in the supine position after resting for 10 min.

The principal investigator or subinvestigator will review the results of observation/examination and document or attach the results (except physical examination) as well as the time of measuring blood pressure and pulse rate (except post-treatment examination) in the CRF.
Normal/abnormal assessment of the 12-lead ECG finding will be documented in the CRF. ECG finding will be evaluated either as "within normal limits" or "clinically significant abnormal finding", and any "clinically significant abnormal finding" should be detailed in the CRF. All the 12-lead ECG data during the study will be evaluated as safety variables. For the time points when 12-lead ECG are recorded for safety evaluation only, the time of recording 12-lead ECG for safety evaluation except post-treatment examination will be documented in the CRF (see "Appendix 2: Schedule of Study Procedures".)
Appropriate measures must be taken when any abnormalities are noted in a subject's health conditions. When an unscheduled examination is required for the subject at the discretion of the principal investigator or subinvestigator, he/she will perform such examination after explaining the reason and obtaining the subject's approval until the laboratory value returns within the laboratory reference range or pre-treatment level to confirm the subject's safety, and document or attach the relevant information in the CRF.
Further investigation (excluding genetic testing) may be conducted to determine the cause of abnormality using remaining specimen for unforeseeable circumstances or when thorough investigation of the safety is required.

<Rationale>
These procedures are intended to investigate the effect on the safety of subjects and on laboratory values.

10.10 Assessment of Abnormal Changes

The principal investigator or subinvestigator will assess abnormal changes of the data for each interval, i.e., from pre-dose in a study period through pre-dose in the succeeding period or post-treatment examination (or early termination when the study was discontinued).

These assessments of abnormal changes will be made for each interval by reviewing the changes from pre- to post-dose of the study drug in body temperature, blood pressure, pulse rate, 12-lead ECG and clinical laboratory tests, and considering whether or not the changes are clinically significant.

When a change is assessed as “abnormal”, it will be handled as an adverse event, documented in the adverse event page of the CRF, and the laboratory value must be followed until it returns to within the reference range or to the pre-treatment level. A follow-up examination may be terminated if there is some medically justifiable reason for it, and the reason should be recorded in the CRF.

When a change is assessed as “not abnormal,” the reason should be documented in the CRF as either “physiological change (within the laboratory reference range or intraindividual variability)” or “other”.

10.11 Pharmacokinetic Measurements

Plasma concentrations of unchanged TS-071 and its metabolites (M-2 and M-17) and plasma concentrations of unchanged moxifloxacin will be determined. Blood will be collected at the time points shown in “Table 10-4 Time Points for Pharmacokinetic Measurements” and time of collection will be documented in the CRF. Any missing blood collection should be explained in the CRF. If blood collection coincides with 12-lead ECG recording, blood will be collected after ECG recording. Acceptable time windows for blood collection are described in “Appendix 3: Acceptable Time Windows for Examinations.”

Collection, handling and shipment of blood specimens are detailed in the “Procedure for the Handling of Specimens.”

Table 10-4 Time Points for Pharmacokinetic Measurements

Variable*	Time after study drug administration
Plasma concentrations of unchanged TS-071<br>Plasma concentrations of TS-071 metabolites (M-2 and M-17)<br>Plasma concentrations of unchanged moxifloxacin	Pre-dose and 0.5, 1, 2, 3, 4, 6, 8, 12, 24, 48 hr post-dose in each study period

*: For the treatment groups of anticipated therapeutic dose and supratherapeutic dose, plasma concentrations of unchanged TS-071 and its metabolites will be determined; for the Avelox group, plasma concentrations of unchanged moxifloxacin will be determined.

<Rationale for the time points>

The time points were selected to include the Tmax values of TS-071 after single administration of 5 mg, 15 mg, 25 mg and moxifloxacin 400 mg (1.06 hr, 1.56 hr, 2.25 hr and 1.75 hr, respectively) in reference to the QT/QTc Guideline as well as those required for pharmacokinetic evaluation and also considering the study feasibility.

10.12 Number of Blood Samplings and Volume of Blood to be Collected

The number of blood samplings and blood volume to be collected from screening examination through post-treatment examination are given in "Table 10-5 Number of Blood Samplings and Volume of Blood to be Collected."

Table 10-5 Number of Blood Samplings and Volume of Blood to be Collected

Examination	Volume of blood per time	Number of samplings	Blood volume
Screening examination	11 mL	1	11 mL
Hematology and blood chemistry tests, blood glucose	9 mL	10	90 mL
Pharmacokinetics (anticipated therapeutic dose group, supratherapeutic dose group, placebo group)	5 mL	33	165 mL
Pharmacokinetics (Avelox group)	7 mL	11	77 mL
Total			343 mL

11. Securing the Safety of Subjects

11.1 Basic Principles

(1) The principal investigator will be responsible for all medical decisions involved in the conduct of the study.

(2) The head of the study site and the principal investigator will assure that all subjects will be provided with adequate medical care for every adverse event that may occur in relation to this study during and after their participation in the study.

(3) The sponsor will be responsible for evaluating the safety of study drugs continuously.

(4) The sponsor will promptly provide the principal investigator and the head of the study site with any information that may affect the safety of subjects, the conduct of the study, or the approval of the IRB for continuation of the study.

11.2 Adverse Events

11.2.1 Definition of Adverse Events

In this study, an "adverse event" is defined as any unfavorable and unintended sign (including an abnormal change in clinical laboratory value), symptom or disease that occurs in a subject administered study drug, whether or not related to the study drug.

11.2.2 Adverse Event Monitoring

For all adverse events occurring during the course of the study, the principal investigator or subinvestigator will document in the CRF the information as described in "11.2.3 Adverse Event Assessments", any therapeutic and other intervention given, and other information required for the safety evaluation of the study drug. All adverse events must be followed until recovery. A follow-up examination may be terminated if

there is some medically justifiable reason as described below, and the reason should be recorded in the CRF.

Examples:

- When the causal relationship of an adverse event to the study drug can be definitely ruled out and it is judged that the safety of the subject can be fully secured.
- When an adverse event is irreversible and the condition of the subject is judged to have become stable.
- When additional follow-up is judged unnecessary by considering the course and other elements of an adverse event after completion of study drug administration.

<Rationale>

These procedures are intended to investigate the effect on subjects and to secure the safety of subjects.

11.2.3 Adverse Event Assessments

(1) Adverse event term

An adverse event term of a symptom/finding, disease, or laboratory value, will be identified.

(2) Date of onset

The date when an adverse event was noted will be the date of onset. For an abnormal change in laboratory value, the date of laboratory test will be the date of onset. Time of onset will be documented for adverse events occurring during hospitalization except abnormal changes in laboratory value. When some sign/symptom was noted and the cause was later identified, the date when the sign/symptom was first noted will be the date of onset.

(3) Severity

Severity of each adverse event will be determined according to the 3 categories characterized in "Table 11-1 Severity of Adverse Events." When the severity of an adverse event has changed during its course, the highest grade will be recorded.

Table 11-1 Severity of Adverse Events

Mild	Transient and does not disrupt normal daily activities (e.g., requires no specific treatment or needs only very light treatment).
Moderate	Disrupts normal daily activities (e.g., requires ambulatory treatment in a clinic)
Severe	Makes normal daily activities impossible (e.g., requires hospitalization for treatment)

(4) Seriousness

Whether an adverse event is serious or non-serious will be determined according to the definition in "11.3.1 Definition of Serious Adverse Events."

1) Serious

2) Non-serious

(5) Action concerning study drug

Action taken concerning the study drug will be determined and documented in the CRF as either;

1) None or

2) Study drug administration discontinued

(6) Medical treatment for adverse event

Any medical treatments provided for adverse event will be documented in the CRF including the name of medication and dosage regimen or therapy. Such information will be captured until completion of the relevant medical treatment. However, this provision will not apply when follow-up of an adverse event is terminated for some medically justifiable reason or when it becomes impossible due to the death of the subject that is not related to the adverse event, change to another hospital or change of address.

(7) Outcome

Outcome of an adverse event will be determined according to the 6 categories characterized in "Table 11-2 Outcome of Adverse Events." For an adverse event with an "unknown" outcome, if its evaluation before becoming impossible to follow up is available, the evaluation will be included for an outcome assessment. Any "unknown" outcome should be explained in the CRF.

Table 11-2 Outcome of Adverse Events

Recovered	Signs/symptoms or findings have resolved or reversed; laboratory values have become normal or returned to pre-treatment level.
Relieved	Signs/symptoms or findings have almost resolved or reversed; laboratory values have showed a tendency to become normal or return to pre-treatment level.
Not recovered	Signs/symptoms or findings have not resolved or reversed; laboratory values have not improved; or they have worsened.
Recovered with sequelae	Some signs/symptoms or findings or some laboratory values have resolved or reversed or improve, but there remain signs/symptoms that are unlikely to resolve or reverse.
Fatal	Occurrence of death directly related to the adverse event in question.
Unknown	The course of an adverse event has become impossible to be followed due to the subject's death unrelated to the adverse event, change to another hospital, change of address or some other reason.

(8) Date of outcome

The date of outcome is defined as the last day when the outcome of an adverse event could be directly confirmed or determined by the principal investigator or subinvestigator. In the case of laboratory values, the day when the laboratory test was performed will be the date of outcome. Time of outcome will be documented when an adverse event (except abnormal laboratory value) resolved during hospitalization. When an adverse event of a subjective symptom resolved during the interval between hospitalizations, the day when the event resolved will be the date of outcome.

An adverse event must be followed until recovery. However, when it is judged not necessary to follow the event until recovery, the day when such judgment was made will be the date of outcome.

When the date of outcome cannot be identified due to the subject's failure to return to the study site or some other reason, the date when the outcome was confirmed or determined by contacting the subject by telephone or correspondence will be the date of outcome.

(9) Causal relationship to study drug and definition of adverse drug reactions

The causal relationship of an adverse event to study drug will be determined according to the 5 categories characterized in "Table 11-3 Causal Relationship to Study Drug." Adverse events other than those determined to be "Not related" to study drug are defined as adverse drug reactions. When an adverse event is determined to be "Not related" to study drug, the reason should be given in the CRF.

Table 11-3 Causal Relationship to Study Drug

Definitely related	There is a temporal relationship between onset of an adverse event and administration of study drug (including the course after discontinuation of study drug), and the factors other than the study drug administered, such as present illness/history of present illness and concomitant medication/therapy, are excluded; or an adverse event is a known adverse drug reaction to the study drug. Or the causal relationship to study drug administered is strongly suggested by the following:<br>• A similar event is noted after re-administration of the study drug
Probably related	There is a temporal relationship between onset of an adverse event and administration of study drug (including the course after discontinuation of study drug), and the factors other than the study drug administered, such as present illness/history of present illness and concomitant medication/therapy, can almost be excluded.
Possibly related	There is a temporal relationship between onset of an adverse event and administration of study drug (including the course after discontinuation of study drug), and the factors other than the study drug administered, such as present illness/history of present illness and concomitant medication/therapy, may be presumed, but the possibility of involvement of study drug cannot be ruled out. For example, similar events have been reported with the study drug in the past or with similar compounds; or such an event can be expected from the pharmacological activity of study drug.
Not related	There is little temporal relationship between onset of an adverse event and administration of study drug. Other factors such as present illness/history of present illness and concomitant medication/therapy are quite likely to be involved.
Unknown	Lack of material by which to determine causal relationship.

11.2.4 Follow-up of Adverse Events

(1) When a medical treatment is necessary to treat an adverse event, the principal investigator or subinvestigator will inform the subject accordingly.

(2) When an adverse event has occurred, the principal investigator or subinvestigator should discontinue the study drug administration where necessary, secure the safety of the subject by providing appropriate medical treatment, and make an effort to find the cause.

(3) All subjects experiencing adverse events should be followed until the events resolved. However, a follow-up of an event may be terminated for some medically justifiable reason as described below, and the reason should be specified in the CRF.

Examples:

- When the causal relationship of an adverse event to study drug can be definitely ruled out and it is judged that the safety of the subject can be fully secured.
- When an adverse event is irreversible and the condition of the subject is judged to have become stable.
- When the effect of the study drug appears to have dissolved and further follow-up is not necessary by considering the course and other elements of the adverse event after completion of study drug administration.

11.3 Serious Adverse Events

11.3.1 Definition of Serious Adverse Events

A "serious adverse event" among adverse events is defined as follows:

(1) An event that results in death.

(2) An event that is life-threatening (i.e. may lead to death).

(3) An event that results in inpatient hospitalization or prolongation of existing hospitalization.

(4) An event that results in persistent or significant disability/incapacity.

(5) An event that results in congenital anomaly.

(6) Other medically important events that may expose the subject to danger, even though the event is not immediately life-threatening or fatal or does not result in hospitalization, or require medical intervention to prevent such outcome as (1) through (5) above.

11.3.2 Procedures in the Event of a Serious Adverse Event

In the event of a serious adverse event, the procedures as shown below must be followed in addition to those described in "11.2.4 Follow-up of Adverse Events."

(1) For all serious adverse events, the principal investigator will inform promptly the head of the study site and the sponsor (monitor) of the event verbally or by telephone or fax or otherwise, followed by a written report using the "Report on Serious Adverse Event Occurring during the Study." In this report, the principal investigator will determine whether or not the serious adverse event is a serious and unexpected adverse drug reaction (i.e., an adverse drug reaction that was not consistent in nature or severity with the information provided in the Investigator's Brochure.)

(2) When additional information on the reported serious adverse event (autopsy report, terminal-stage medical record or other information) is requested by the sponsor, the head of the study site or the IRB, the principal investigator will provide them with such information.

(3) The sponsor will report every 6 months all serious adverse drug reactions of which causal relationship with the study drug cannot be ruled out to the principal investigator and the head of the study site. Serious adverse events that are unexpected from the information provided in the investigator's brochure will be immediately reported to the principal investigator and the head of the study site.

11.3.3 Contact Information in the Event of a Serious Adverse Event

- Weekdays from 9:00 to 18:00: Clinical Research, Taisho Pharmaceuticals Co., Ltd.
  Phone: 03-3985-1306 (direct line) FAX: 03-3985-0650

- Weekdays from 18:00 to 9:00, Saturdays, Sundays, national holidays, yearend and new-year holidays, and summer holidays: Emergency Contact Center
  Phone: 0120-119-781

11.4 Predictable Adverse Drug Reactions

11.4.1 Adverse Drug Reactions Predictable from Nonclinical Studies of TS-071

The toxic findings reported in nonclinical studies of TS-071 included soft stools, diarrhoea, watery stools, suppression of body weight gain, decrease in body weight, dark-red focus and erosion in glandular stomach, macroscopic dilatation, dark-red focus, weight increase, mucosal thickening and perforating ulcer in cecum, increased trabecular bone in femur and sternum, decreases in erythrocytes count, hemoglobin and hematocrit, and decrease in the number of corpora lutea. In a one-month repeated-dose oral toxicity study in rats, the death of an animal receiving 500 mg/kg of TS-071 occurred on Day 25 of treatment possibly due to worsening of general condition.

In addition, the adverse events that may occur in association with the pharmacological properties of TS-071 include an increase in urine volume, dehydration, urinary tract infection, an effect on ketones in urine, hypoglycemia, an effect on renal function and an increase in urinary excretion of electrolytes (Ca, P, etc.) due to the promotion of urinary glucose excretion.

11.4.2 Adverse Drug Reactions Predictable from Clinical Studies of TS-071

The adverse drug reactions reported in the Phase I single dose study include occult blood positive, β_2 microglobulin urine increased, diarrhoea (soft stools) and urethritis. All these adverse drug reactions were mild in severity and reversible. In the Phase I 7-day repeated-dose study, a transient and mild diarrhoea (soft stools) were observed. A transient and mild constipation was reported in the clinical pharmacology study of 7-day repeated administration in patients with type 2 diabetes.

The adverse drug reactions occurring 2 events or more in the Phase II exploratory study include diarrhoea/soft stools, thirst, cystitis, β-N-acetyl-D-glucosaminidase increased, blood urine present, urine ketone body present, headache and pollakiuria. All these adverse drug reactions recovered or were recovering during the treatment period of after the study.

The adverse drug reactions reported in the drug interaction studies include one event of white blood cells urine positive in one subject receiving TS-071 concomitantly with glimepyride, 2 events of nausea in 2 subjects and one event of headache in one subject receiving TS-071 concomitantly with metformin. All these adverse drug reactions were mild in severity and reversible. There were no adverse drug reactions reported after concomitant administration with pioglitazone, voglibose and citagliptin in the drug interaction studies.

The adverse drug reactions reported in the clinical pharmacology study in patients with type 2 diabetes with renal dysfunction were rash and dizziness, both of which were transient and mild in severity.

In the Phase II confirmatory study, adverse drug reactions occurring 2 events or more include constipation, pollakiuria, polyuria/urine output increased, thirst, malaise, pruritus genital, albumin urine present, β_2 microglobuin urine increased, urine ketone body present, all of which were mild in severity.
Adverse drug reactions that are predicted to occur include effects on renal function, electrolytes and circulating plasma volume, hypoglycemia and genital and urological infections since TS-071 promotes urinary glucose excretion by inhibiting reabsorption of glucose in the renal tubules.

11.4.3 Measures to be Taken in the Event of Hypoglycemia

In the nonclinical studies [SGLT inhibitory effect in human expressing cells (*in vitro*)], TS-071 demonstrated an inhibitory effect of SGLT and it also inhibited SGLT1, although the effect was not as potent as it inhibited SGLT2: the 50% inhibitory concentration (IC_{50}) was 2.26 nmol/L for SGLT2 and 2900 nmol/L for SGLT1. The results of a series of efficacy pharmacology studies suggested that TS-071 exerts its hypoglycemic effect by inhibiting SGLT2 in the proximal renal tubules and promoting urinary glucose excretion. However, the mechanism of suppression of glucose absorption by inhibiting SGLT1 in the small intestines also conceivable. When a hypoglycemic symptom occurred, oral or intravenous glucose should be administered, depending on the symptom, e.g., oral dose for a mild symptom and intravenous dose when the symptom persists, or is severe or emergent.

11.4.4 Adverse Drug Reactions Predictable from Clinical Studies of Avelox® Tablets

The package insert of Avelox® tablets is located in the Attachment.
In the clinical studies of Avelox® tablets in Japan up to the time of approval, adverse drug reactions (including abnormal changes in laboratory tests) were experienced by 130 out of 505 subjects (25.7%). The major events include diarrhoea in 24 subjects (4.8%), liver function test abnormal in 22 subjects (4.4%), nausea in 18 subjects (3.6%), dyspepsia in 14 subjects (2.8%) and abdominal pain in 12 subjects (2.4%). In the foreign clinical studies adverse drug reactions were experienced by 2314 out of 9225 subjects (25.1%). The major events include nausea in 653 subjects (7.1%), diarrhoea in 461 subjects (5.0%) and dizziness in 233 subjects (2.5%).

11.4.5 Measures to be Taken in the Event of Adverse Drug Reaction or Adverse Event Suggestive of TdP

When an adverse drug reaction or adverse event suggestive of TdP occurred, the principal investigator or subinvestigator should discontinue the study drug administration of the subject and the enhancement factors, e.g., hypokalemia and bradycardia, must be corrected. Emergency resuscitation device including a defibrillator will be prepared where necessary. The subject will be sent to a specialized hospital if appropriate measures cannot be taken in the study site. Use of magnesium sulfate by intravenous administration (2 g for 2 to 5 min) should be considered to prevent repeated TdP. Since bradycardia is likely to induce TdP, it should be treated to increase heart rate by a drip infusion of isoproterenol, intravenous administration of atropine sulfate or external pacing.

12. Criteria and Procedures for Early Termination of a Subject

12.1 Criteria for Early Termination

An early termination is defined as "an occasion when the study is prematurely discontinued by the principal investigator or subinvestigator or because of a subject's inconvenience or his/her own will before completion of the protocol-directed study procedures."

The study of a subject will be discontinued according to the procedures provided in "12.2 Procedures for Early Termination" on the occurrence of any of the followings:

(1) When it is difficult to continue the study because of an adverse event.

(2) When a QTcF interval of 12-lead ECG for safety assessments increased to ≥ 500 msec or increased by > 60 msec from the baseline (prior to study drug administration in each study period), and the principal investigator or subinvestigator finds it difficult to continue the study.

(3) When a QRS interval of 12-lead ECG for safety assessments increased by ≥ 25% from the baseline (prior to study drug administration in each study period), and the principal investigator or subinvestigator finds it difficult to continue the study.

(4) When a significant or continued non-compliance with the protocol that will make it difficult to secure safety or confound the evaluation was identified.

(5) When a subject is found ineligible in terms of any of the inclusion and exclusion criteria.

(6) When a subject wishes to withdraw from the study because of an adverse event.

(7) When a subject wishes to withdraw from the study for a reason other than adverse events.

(8) When a subject fails to return to the study site and it is difficult to continue the study.

(9) When the principal investigator or subinvestigator finds it difficult to continue the study for reasons other than those described above.

<Rationale>

(1) This is in consideration of the safety of subjects.

(2) The QT/QTc Guideline specifies that if there is a marked prolongation of QT/QTc interval to > 500 msec or increase of > 60 msec over the baseline, discontinuation of a subject from a study should be considered.

(3) "The manual for handling disorders due to adverse drug reactions (MHLW, May, 2009)" states that the dosage should be decreased or drug administration should be discontinued if a QRS interval increased by ≥ 25% compared to the pre-treatment level.

(4) This is to eliminate the possibility of compromising the ethical and scientific appropriateness of the study.

(5) This is intended to ensure early termination of the study for a subject who fails to meet the entry criteria.

(6), (7) These are to respect the free will of subjects.

(8) This will interfere with the protocol-directed evaluation and observation.

(9) This is to provide against unforeseen contingencies that may require early termination of the study.

12.2 Procedures for Early Termination

When a subject meets any of the criteria provided in "12.1 Criteria for Early Termination" during the course of the study, the principal investigator or subinvestigator will perform the study procedures at early termination (see "Appendix 2: Schedule of Study Procedures" and document the results in the CRF. Date of and reason for early termination will also be documented in the CRF.

After discontinuation of the study, appropriate measures should be taken for the subject. When the study was prematurely terminated due to a safety problem, appropriate medical treatments should be provided to the subject and procedures described in "11. Securing the Safety of Subjects" should be followed.

When a subject failed to return to the study site, efforts should be made to ask the subject to return and undergo the study procedures provided in "Appendix 2: Schedule of Study Procedures."

When it is impossible to perform the study procedure for early termination, the principal investigator or subinvestigator will immediately contact the subject by telephone, correspondence or any other possible means to make a follow-up on the clinical course of the subject after the last observation and any occurrence of an adverse event, and document the results in the CRF. When necessary information on the subject was captured from a person other than the subject for some reason, the reason and the relation of the person to the subject will be documented in the CRF.

13. Statistical Analysis

13.1 Purpose of Analysis

The purpose of analysis is to evaluate the effect of anticipated therapeutic dose and supratherapeutic dose of TS-071 on QT/QTc interval with QTcF as the primary endpoint and QTcB as the secondary endpoint, and also to confirm the assay sensitivity of the study by employing moxifloxacin (Avelox® tablets) as a positive control.

Another purpose of analysis is to evaluate the safety of anticipated therapeutic dose and supratherapeutic dose of TS-071 with adverse events and adverse drug reactions as evaluation variables.

In addition, the relation of the pharmacokinetics of TS-071 and moxifloxacin to QTc interval will be investigated with plasma concentrations of unchanged TS-071 and unchanged moxifloxacin as evaluation variables.

13.2 Analysis Sets

Three kinds of analysis sets are defined: "12-lead ECG analysis set for QT/QTc analysis" for QT/QTc analysis, "Safety analysis set" for safety evaluation and "Pharmacokinetic analysis set" for pharmacokinetic evaluation. The definition of each analysis set is as follows:

(1) 12-lead ECG analysis set for QT/QTc analysis

This analysis set will include all subjects who fulfilled the minimum requirements of the study without any major protocol deviation and for whom 12-lead ECG data for QT/QTc analysis is available. Subjects who are excluded from analysis according to the "Criteria for Handling of Cases and Data" (which will be separately provided) will be excluded from this set.

(2) Safety analysis set

This analysis set will include all subjects who received at least one dose of study drug and for whom safety variable is available. Subjects who are excluded from analysis according to the "Criteria for Handling of Cases and Data" will be excluded from this set.

(3) Pharmacokinetic analysis set

This analysis set will include all subjects who fulfilled the minimum requirements of the study without any major protocol deviation and for whom pharmacokinetic variable is available. Subjects who are excluded from analysis according to the "Criteria for Handling of Cases and Data" will be excluded from this set.

13.3 Handling of Cases and Data

13.3.1 Handling of Cases

Handling of cases will be specified in the "Criteria for Handling of Cases and Data", which will be provided separately.

13.3.2 Handling of Data

Handling of data will be specified in the "Criteria for Handling of Cases and Data", which will be provided separately. Handling of data for summary/analysis is specified below.

13.3.2.1 Acceptability of Data

The acceptable time windows for each examination are given in "Appendix 3: Acceptable Time Windows for Examinations." The acceptable range for data will be specified in the "Criteria for Handling of Cases and Data."

13.3.2.2 Handling of Dates

When a date cannot be identified and documented as beginning, middle, end or unknown period of a month, it will be handled as "5th", "15th", "25th" or "15th" of a month, respectively. When a month cannot be identified, it will be handled as "June", and when a year cannot be identified, it will be handled as "unknown." When calculating a time period using dates and a negative value of the period is not reasonable but the value is calculated as a negative value, the time period will be handled as "0 day".

13.3.2.3 Handling of Missing Values

When a data is missing or excluded from analysis, continuous value of such data will be excluded from summary/analysis, and discrete value of such data will be handled as unknown.

13.3.2.4 Handling of Abnormal Data

Abnormality of data will be reviewed before data lock. Abnormal data will be excluded from summary/analysis as abnormal data if the exclusion can be explained reasonably. Abnormality of data will be reviewed from an overall medical point of view.

13.3.2.5 Handling of Values below/above Limit of Quantification

When clinical laboratory values or other laboratory values are below or above limit of quantification, the quantification limit values will be used in summary/analysis for continuous data, and the observed values will be used in summary/analysis for discrete data.

13.4 Statistical Analysis Plan

The person responsible for statistical analysis will perform the statistical analysis immediately after data lock based on a statistical analysis plan in accordance with the sponsor's relevant standard operating procedure.
The statistical analysis plan, which will provide further details regarding the contents in this section, will be prepared and fixed prior to the summary/analysis procedure.
When the statistical analysis plan needs to be revised due to a deviation from the original plan or for some other reasons, the person responsible for statistical analysis will create a record specifying the time of amendment, changes made, reason for amendment and the person responsible for amendment in accordance with the sponsor's relevant standard operating procedure.

13.4.1 Significance Level and Confidence Coefficient

The significance level of 5% (two-sided) will be applied in statistical significance testing unless otherwise specified. The confidence coefficient of 95% (two-sided) will be used unless otherwise specified.

13.4.2 Demographics and Other Baseline Characteristics

Regarding subject background characteristics, frequency will be presented for discrete data by treatment sequence, and summary statistics will be provided for continuous data by treatment sequence.

13.4.3 Evaluation Variables and Summary/Analysis Plan

13.4.3.1 Variables for QT/QTc Interval Analysis and Summary/Analysis Plan

<Variables>
QTcF will be the primary endpoint and QTcB will be the secondary endpoint.

<Summary/Analysis plan>
The following analyses will be performed in the 12-lead ECG analysis set for QT/QTc analysis:

(1) Calculation of summary statistics
Summary statistics will be provided for changes from baseline (prior to study drug administration in each study period) in QTc (ΔQTc) at each time point after administration and the 95% one-sided confidence intervals will be presented.

(2) Analyses of central tendency
Treatment comparisons between anticipated therapeutic dose or supratherapeutic dose (hereinafter referred to as TS-071 groups) and placebo (ΔΔQTc) will be analyzed using analysis of covariance with ΔQTc at each time point as a variable. Least square means and the upper bound of the 95% one-sided confidence intervals of ΔΔQTc at each time point will be presented. The lower bound of 95% one-sided confidence interval will be presented similarly for Avelox treatment.
The effect of TS-071 on QT/QTc interval prolongation will be regarded negative when the upper

bound of the 95% one-sided confidence interval for the mean effect on the QT/QTc interval excludes 10 msec at all the time points in the TS-071 groups. The assay sensitivity will be regarded as established when the lower bound of the above-described 95% one-sided confidence interval excludes 0 msec at any of the time points in the Avelox treatment.

(3) Categorical analyses

The number and percentage of subjects whose QTc value after study drug administration exceeded 450 msec, 480 msec or 500 msec will be calculated by treatment group. The number and percentage of subjects whose ΔQTc value exceeded 30 msec or 60 msec by treatment group.

(4) Graphical presentation

Scatter plots of QTc and RR will be presented by treatment group to verify the validity of QT corrections. Scatter plots of ΔΔQTcF and plasma concentrations will be presented for TS-071 groups and Avelox treatment to investigate the relationship between ΔΔQTcF and plasma concentrations.

13.4.3.2 Analysis Plan for Safety Variables

<Variables>

(1) Adverse events and adverse drug reactions, including abnormal changes in body temperature, blood pressure, pulse rate, 12-lead ECG and clinical laboratory tests

(2) Effects on body temperature, blood pressure, pulse rate, 12-lead ECG and clinical laboratory tests

<Summary/Analysis plan>

Following analyses will be performed in the safety analysis set:

Frequency of adverse events and adverse drug reactions based on each symptom and finding will be presented.

Number of adverse events and adverse drug reactions will be summarized by relationship to study drug and also by relationship to study drug ("Not related" and other than "Not related") and severity.

The incidence (%) of adverse events and adverse drug reactions will be calculated. Summary statistics will be provided for body temperature, blood pressure, pulse rate and clinical laboratory tests at each time point, and changes from baseline (prior to study drug administration in each study period) will also be summarized by summary statistics.

13.4.3.3 Analysis Plan for Pharmacokinetic Variables

<Variables>

Plasma concentrations of unchanged TS-071 and its metabolites (M-2 and M-17), and unchanged moxifloxacin

<Summary/Analysis plan>

Pharmacokinetic parameters including Cmax, Tmax, AUC and $T_{1/2}$ of the pharmacokinetic variables will be determined by noncompartment analysis in the pharmacokinetic analysis set.

13.4.4 Statistical Interpretation of Analysis Results

Effects on QT/QTc prolongation in the TS-071 groups and assay sensitivity of the study will be interpreted as

described in "13.4.3.1 Variables for QT/QTc Interval Analysis and Summary/Analysis Plan."
Clinical interpretation of the analysis results will be evaluated comprehensively by reviewing all the variables and described in the clinical study report.

13.4.5 Multiplicity Adjustment

The effect of TS-071 on QT/QTc interval prolongation will be regarded negative when the upper bound of the 95% one-sided confidence interval for the mean effect on the QT/QTc interval excludes 10 msec at all the time points in the TS-071 groups. There is no need for multiplicity adjustment because alternative hypothesis is expressed as an intersection of alternative hypothesis at each time point.

13.4.6 Interim Analysis

No interim analyses will be performed in this study.

13.5 Finalization of Criteria for Handling of Cases and Data

The "Criteria for Handling of Cases and Data" will be finalized prior to data lock. For finalizing the criteria, the handling of each data and case as well as the handling of problematic cases which were not stipulated when planning the protocol will be reviewed upon consulting with the medical expert where necessary.
When any of the handling criteria is changed, it should be described in detail in the clinical study report including the time of change, details of amendments and the reason.

13.6 Finalization of Statistical Analysis Plan

The statistical analysis plan will be finalized after the sponsor reviews the appropriateness of the summary/analysis methods and necessity to define the handling of data to be analyzed by the methods other than those described in the statistical analysis section of the protocol.
When the statistical analysis plan is revised, it should be documented by specifying the time of amendment, details of amendments, reason and the person responsible for amendment. The amendment will also be described in the clinical study report.

14. Protocol Compliance, Deviations or Changes and Amendments

14.1 Protocol Compliance and Deviations or Changes

The principal investigator or subinvestigator should not deviate from or change the protocol without prior written agreement between the principal investigator and the sponsor and without prior review and written approval of the IRB except where necessary to eliminate an immediate hazard to subjects or for clerical changes such as a change of phone number.

(1) The principal investigator or subinvestigator may deviate from or change the protocol to eliminate an immediate hazard to study subjects without prior written agreement between the principal investigator and the sponsor and without prior written approval of the IRB. In this regard, the principal investigator will notify the sponsor of the deviation or change together with its reason, and discuss and agree the proposed protocol amendment with the sponsor. A draft protocol amendment

should be reviewed and approved by the IRB via the head of the study site without delay.

(2) The investigator must immediately report to the IRB via the sponsor and the head of the study site every change in the protocol that will have significant impact on the conduct of the study or increase the hazard to subjects.

(3) The principal investigator or subinvestigator will record all actions deviated from the protocol. As to protocol deviations to eliminate an immediate hazard to study subjects or for other medically compelling reasons, the principal investigator will immediately provide the sponsor and the head of the study site with a document explaining the reason and retain the copy thereof.

14.2 Protocol Amendments

The protocol and the sample CRF will be revised according to the following procedure:

(1) When the protocol or CRF needs to be revised, the sponsor will provide the principal investigator with a draft amendment of the document and other necessary material or information.

(2) The sponsor will allow the principal investigator sufficient time to review and discuss with the sponsor the draft amendment and other information provided by the sponsor.

(3) After discussing with the principal investigator, the sponsor will agree with the principal investigator on the amendments of the protocol or CRF and compliance with the revised protocol. In witness of this agreement, the sponsor and the principal investigator will sign/seal and personally date a statement of mutual agreement on the amendments. A similar procedure will be followed when an amendment in the protocol or CRF requested by the head of the study site based on the IRB's opinion is acceptable to the sponsor and the document is revised.

(4) The sponsor will immediately submit the revised protocol or CRF to the head of the study site for approval by the IRB. When there are clerical changes in the protocol (e.g., changes of monitors or monitor's phone number), additions or deletions of study site, or changes in investigators at other study sites, the sponsor will notify the head of the study site and the principal investigator accordingly.

15. Study Completion, Premature Termination or Suspension

15.1 Study Completion

The date of study completion for each subject is defined as "the date when the protocol-directed study procedures are completed or the date of early termination." The date of study completion for each study site is defined as "the last date of study completion for subjects enrolled in the relevant study site."

(1) After completing the protocol-directed study drug administration and study procedures to the last subject at the relevant study site, the principal investigator will provide the head of the study site with a written report of study completion outlining the study results.

(2) The head of the study site will immediately report the study completion in writing to the IRB and the sponsor and provide them with a summary of study results based on the principal investigator's report.

15.2 Premature Termination or Suspension of Study

15.2.1 Criteria for Premature Termination or Suspension of Study

When the sponsor finds it difficult or of no significance to continue the study due to any of the following reasons, premature termination or suspension of the study will be discussed with the study site and medical expert without delay:

(1) An "unexpected" serious adverse event (illness, disorder or death) has occurred.

(2) Information has become available showing that the occurrence trend of "unexpected" significant adverse events, e.g., number of events and their frequency and conditions of occurrence, are unpredictable from the information available in the Investigator's Brochure.

(3) Information has become available showing that the occurrence trend of adverse drug reactions, e.g., number of events and their frequency and conditions of occurrence, are significantly worsening.

(4) Other information has become available that may impact on the study continuation.

15.2.2 Procedure for Premature Termination or Suspension of Study

(1) When the study is prematurely terminated or suspended, the sponsor will immediately notify the head of the study site and the regulatory authority accordingly in a written form specifying the reason.

(2) When the head of the study site receives notification of premature termination or suspension of the study from the sponsor, he/she will immediately inform the principal investigator and the IRB accordingly in a written form specifying the details.

(3) When the principal investigator receives notification of premature termination or suspension of the study, he/she will immediately inform the subjects accordingly and take appropriate measures.

15.3 Premature Termination or Suspension of Study at the Study Site

15.3.1 Criteria for Premature Termination or Suspension of Study at the Study Site

The sponsor will consider whether or not to continue the study at the study site when any of the following criteria is satisfied:

(1) The protocol needs to be amended and the study site is unable to comply with such amendments.

(2) The sponsor is unable to accept the amendments of the study directed by the head of the study site based on the IRB's opinion.

(3) The head of the study site instructs the sponsor to prematurely terminate the study based on the IRB's opinion.

(4) The study site is found in a significant or continuous violation of GCP, the protocol or the contractual agreement.

15.3.2 Procedure for Premature Termination or Suspension of Study at the Study Site

The study at the study site will be prematurely terminated or suspended according to the following procedure:

(1) When the study is prematurely terminated or suspended by the principal investigator, he/she will

immediately notify the head of the study site accordingly in a written form specifying the details.

(2) When the head of the study site receives notification of premature termination or suspension of the study by the principal investigator, he/she will immediately inform the sponsor and the IRB accordingly in a written form specifying the details.

(3) When the head of the study site receives notification of premature termination or suspension of the study from the IRB, he/she will immediately inform the principal investigator and the sponsor in a written form specifying his/her instructions and decisions.

16. Case Report Forms (CRFs)

16.1 Completion and Submission of CRFs

(1) The principal investigator will complete the CRFs for all subjects receiving study drug according to "16.3 Guidance for Completion of CRFs." The CRFs will be signed/sealed and personally dated, submitted to the sponsor, and the duplicates thereof will be retained by the principal investigator. The subinvestigator completing the CRFs will also sign/seal them.

(2) All CRFs completed by the subinvestigator will be reviewed, checked, signed/sealed and personally dated (date of confirmation) before they are submitted to the sponsor. All changes/amendments made by the subinvestigator will be reviewed, signed/sealed and dated similarly by the principal investigator.

(3) The principal investigator will be responsible for the accuracy and completeness of the CRFs to be submitted to the sponsor.

(4) CRF entries that are based on the source documents must be consistent with the latter. Any discrepancies must be explained in a written report by the principal investigator, which will be provided to the sponsor, and a duplicate of which will be retained by the principal investigator.

(5) The study site person who is appointed as a clinical research coordinator of the study by the head of the study site and authorized to transcribe the data in the source documents to the CRFs is allowed to assist in completing CRFs.

(6) Reports on pharmacokinetic measurements will be produced and directly provided to the sponsor by the drug assay laboratories.

16.2 Changes or Amendments of CRF Entries

(1) The principal investigator or subinvestigator will make changes or amendments in the CRF entries according to "Guidance for Completion of CRFs."

(2) The principal investigator will provide the sponsor with "Record on Changes or Amendments of CRF Entries" and retain a duplicate thereof.

(3) The CRFs submitted by the principal investigator to the sponsor will also serve as a "Record on Changes or Amendments of CRF Entries."

16.3 Guidance for Completion of CRFs

(1) CRFs will be entered by using a writing tool such as indelible black ballpoint pen.

(2) Amendments or deletions of entries or additional entries after the date of preparation will be made by crossing out with a double line so that the original entries remain legible, and by a signature or seal/sign with the date. Changes in important data should be accompanied by an explanation. Important data will include the subject identification code, entries relating to informed consent, assessment of eligibility (except comments), normal/abnormal assessment of 12-lead ECG, normal/abnormal assessment of urine sediment, abnormal change in examinations (body temperature, blood pressure, pulse rate, 12-lead ECG and clinical laboratory tests), entries relating to adverse events (presence/absence of adverse event, adverse event term, severity, seriousness and relationship to study drug) and entries relating to early termination (except comments.)

(3) The signatures/seals of the principal investigator, subinvestigator and clinical research coordinator to be used in the CRFs must be consistent with those presented in a separate "List of Signatures and Seal Impressions."

(4) Except for blank spaces to be used for follow-up examination and documentation of medical history, concomitant medications and adverse events, all blank spaces will be crossed out with a diagonal stroke or explained to distinguish them from missing entries.

(5) Strips of laboratory reports will be attached to the relevant pages of the CRF. The principal investigator, subinvestigator or clinical research coordinator will sign or affix a seal over the edges of the two documents.

16.4 Data Entered in CRFs as Source Data

The following data entered in the CRFs will be regarded as source data unless documented elsewhere such as medical records.

(1) Abnormal change assessments and reasons for no abnormal change of body temperature, blood pressure, pulse rate, 12-lead ECG and clinical laboratory tests

(2) Normal/abnormal assessments of 12-lead ECG

(3) Normal/abnormal assessments of urine sediment

(4) Severity, seriousness and outcome of adverse events and relationship to study drug

(5) Reasons for early termination

(6) All comments

17. Direct Access to Source Documents

17.1 Direct Access to Source Documents

The principal investigator and the head of the study site will accept the sponsor's monitoring and auditing activities and inspection by the IRB and the regulatory authority, and grand direct access to all study-related records including source documents.

17.2 Direct Access Procedure

Direct access procedure will be determined through mutual discussion by the study site and the sponsor.

18. Quality Control and Quality Assurance for the Study

The sponsor will implement and maintain quality assurance and quality control systems based on the relevant standard operating procedures to ensure that the study is conducted and data are generated, recorded and reported in compliance with the protocol, Pharmaceutical Affairs Law and GCP.

18.1 Quality Control

The sponsor will implement quality control at each stage of data handling to ensure that all study-related data are reliable and processed correctly.

The sponsor-designated monitors will verify that the study is conducted in compliance with the protocol, standard operating procedures and GCP through monitoring visits to the study site including direct access to the source documents and other study-related records.

The monitors and the person responsible for quality control system, data management and statistical analysis will implement quality control at each stage of data handling in accordance with the sponsor-directed standard operating procedures.

18.2 Quality Assurance

The sponsor will organize an audit unit independent of the unit responsible for the study conduct to ensure the quality of the study. The auditors will ensure the quality of the study at the sponsor or through auditing visits to the study site and other study facilities if necessary, at appropriate times in accordance with the sponsor's standard operating procedure.

19. Ethical Conduct of the Study

19.1 IRB Review

Conduct of this study will be reviewed from ethical, scientific and medical standpoints by the IRB selected by the study site.

19.2 Continuing Review

Continuing of this study at the study site will be reviewed at least once annually or in any of the following cases.

(1) The sponsor has notified the head of the study site of an occurrence of serious and unexpected adverse drug reaction.

(2) The principal investigator has notified the head of the study site of an occurrence of a serious adverse event.

(3) The principal investigator has informed the head of the study site of an amendment of informed

consent form and other written information for subjects.

(4) The principal investigator has submitted to the head of the study site a report outlining the study progress for continuing review.

(5) The head of the study site finds that a continuing review is necessary for reasons other than those described above.

The head of the study site will seek an opinion from the IRB as to whether it will be appropriate to continue the study at the study site.

19.3 Subject Confidentiality

Subjects will be identified by using their identification codes to protect their privacy and personal information. The parties involved in this study affirm and uphold the principle of protection of the subject's privacy and personal information in creating and handling of CRFs, monitoring, auditing and other study activities, and publishing the study results by the sponsor.

20. Record Retention

20.1 Head of the Study Site

The head of the study site will retain the study-related records until the day specified in either (1) or (2), whichever is the later. These records include source documents to be retained by the study site, contractual agreements, informed consent form and other written information for subjects, protocol and drug dispensing records. When the sponsor requires a longer retention, the head of the study site will consult with the sponsor about the period and methods of retention. The head of the study site will appoint person responsible for the retention of each record. The head of the study site and the person responsible for record retention will take necessary measures to prevent the records from loss or disposal during the retention period and to present them whenever requested.

(1) The date of approval for manufacture and distribution of the relevant investigational product (when the sponsor notifies that the study has been terminated, until at least 3 years after the date of development discontinuation)

(2) The day at least 3 years after the date of the termination or completion of the study

20.2 IRB Organizer

The IRB organizer will retain relevant records until the day specified in either (1) or (2), whichever is the later. These records include standard operating procedures, list of IRB members (including their qualifications), list of IRB members' occupations and affiliations, material related to contractual agreements, documents submitted to the IRB, IRB meeting minutes and correspondence exchanged. When the sponsor requires a longer retention, the IRB organizer will consult with the sponsor about the period and methods of retention. These records will be presented to the regulatory authority when requested.

The IRB organizer will present the standard operating procedures and list of members of the IRB when

requested by the head of a study site (who is not the organizer of the relevant IRB and who seeks an opinion from the IRB) or the sponsor.

(1) The date of approval for manufacture and distribution of the relevant investigational product (when the sponsor notifies that the study has been terminated, until the date of development discontinuation)

(2) The day at least 3 years after the date of the termination or completion of the study

20.3 Principal Investigator

The principal investigator will retain the study-related documents and records in accordance with the instructions from the head of the study site.

20.4 Sponsor

(1) The sponsor will retain the records (including documents and data) to be retained by the sponsor until the day specified in either (1) or (2), whichever is the later.

  1) The date at least 5 years after the date of approval for manufacture and distribution of the relevant investigational product (in case of discontinuing its development, until at least 3 years after the date of development discontinuation), or the date of completing the re-examination when the investigational product is subject to re-examination and the time to its completion is 5 years or more.

  2) The day at least 3 years after the date of the termination or completion of the study

(2) When the sponsor decides that the records (including documents and data) no longer need to be retained by the head of the study site or the IRB organizer, they should notify the head of the study site accordingly and to the IRB organizer via the head of the study site.

21. Payments

Payments for the study will be made to the study site by the sponsor in accordance with the relevant contractual agreement.
Payments to the subjects will be made by the study site in accordance with its relevant internal regulations.

22. Insurance

The sponsor will take appropriate measures such as insurance to ensure the compensation and indemnity liability in case of study-related health injury or loss of subjects.

23. Compensation/Indemnity for Study-Related Health Injury

In the event of a health injury to a subject as a result of participating in the study, the study site and the sponsor will follow the procedures as described below for the subject or aggrieved family:

(1) The study site will make every effort to treat the health injury.

(2) When the health injury has not caused through the intentional or unintentional negligence of the

sponsor and there is no other party that is legally responsible for it, the sponsor will compensate for the health injury under the compensation system. However, if the injury is proven to have been caused through the subject's own intentional or gross negligence, the compensation may be reduced or no compensation may be provided. If any new health injury that has developed is not causally related to this study, no compensation will be provided for it.

(3) When a study-related health injury has occurred through the intentional or unintentional negligence of the sponsor, the study site, or any other party involved in this study under contract with the sponsor or the study site, the party that is legally responsible for the health injury will compensate for it.

24. Reporting and Publication

The sponsor is entitled to use the information obtained from this study for such purposes as an application for approval of manufacture and distribution of the investigational product.

25. References

(1) Outline of Patient Survey 2008, Ministry of Health, Labour and Welfare, 2009.

(2) Outline of National Health and Nutrition Survey 2007, Ministry of Health, Labour and Welfare, 2008.

(3) The diabetes control and complications trial research group. The effect of intensive treatment of diabetes on the development and progression of long-term complications in insulin-dependent diabetes mellitus. N. Engl. J. Med. 1993; 329: 977-86.

(4) UK prospective diabetes study (UKPDS) group. Intensive blood-glucose control with sulphonylureas or insulin compared with conventional treatment and risk of complications in patients with type 2 diabetes (UKPDS 33). Lancet 1998; 352: 837-53.

(5) Ohkubo Y, Kishikawa H, Araki E, Miyata T, Isami S, Motoyoshi S, et al. Intensive insulin therapy prevents the progression of diabetic microvascular complications in Japanese patients with non-insulin-dependent diabetes mellitus: a randomized prospective 6-year study. Diabetes Res. Clin. Pract. 1995; 28: 103-17.

(6) The DECODE study group on behalf of the European diabetes epidemiology group. Glucose tolerance and mortality: comparison of WHO and American Diabetes Association diagnostic criteria. Lancet 1999; 354: 617-21.

(7) Tominaga M, Eguchi H, Manaka H, Igarashi K, Kato T, Sekikawa A. Impaired glucose tolerance is a risk factor for cardiovascular disease, but not impaired fasting glucose. The funagata diabetes study. Diabetes Care 1999; 22: 920-4.

(8) Silverman, M and R. J. Turner. Glucose transport in the renal proximal tubule. Handbook of Physiology ~ Renal Physiology. Am. Physiol. Soc. 1992; 8: 2017-38.

(9) Kanai Y, Lee WS, You G, Brown D and Hediger MA. The human kidney low affinity Na+/glucose cotransporter SGLT2. Delineation of the major renal reabsorptive mechanism for D-glucose. J. Clin. Invest. 1994; 93: 397-404.

(10) You G, Lee WS, Barros EJ, Kanai Y, Huo TL, Khawaja S. et al. Molecular characteristics of Na+ -coupled glucose transporters in adult and embryonic rat kidney. J. Biol. Chem. 1995; 270: 29365-71.

(11) Santer R, Kinner M, Lassen CL, Schneppenheim R, Eggert P, Bald M. et al. Molecular analysis of the SGLT2 gene in patients with renal glucosuria. J. Am. Soc. Nephrol. 2003; 14: 2873-82.

(12) Komoroski B, Vachharajani N, Boulton D, Kornhauser D, Geraldes M, Li L, Pfister M. Dapagliflozin, a novel SGLT2 inhibitor, induces dose-dependent glucosuria in healthy subject. Clin. Pharmacol. Ther. 2009; 85: 520-6.

(13) Komoroski B, Vachharajani N, Feng Y, Li L, Kornhauser D, Pfister M. Dapagliflozin, a novel, selective SGLT2 inhibitor, improved glycemic control over 2 weeks in patients with type 2 diabetes mellitus. Clin. Pharmacol. Ther. 2009; 85: 513-9.

(14) List JF, Woo V, Morales E, Tang W, Fiedorek FT. Sodium-glucose co-transport inhibition with Dapagliflozin in type 2 diabetes. Diabetes Care. 2009; 32: 650-7.

(15) "Guideline for Bioequivalence Studies of Generic Products" (PFSB/ELD Notification No.487 dated December 22, 1997, No.786 dated May 31, 2001 and No.1124004 dated November 24, 2006)

(16) Hulhoven R, Rosillon D, Letiexhe M, Meeus MA, Daoust A, Stockis A. Levocetirizine does not prolong the QT/QTc interval in healthy subjects: results from a thorough QT study. Eur. J. Clin. Pharmacol. 2007; 63: 1011-7.

(17) Kubitza D, Mueck W, Becka M. Randomized, double-blind, crossover study to investigate the effect of Rivaroxaban on QT-interval prolongation. Drug Safety 2008; 31: 67-77.

<Appendix 1>

Protocol No.: TS071-02-11
Version No.: Ver.2
Date of preparation: October 13, 2011

Study Administrative Structure

1 Sponsor

1.1 Sponsor

Taisho Pharmaceutical Co., Ltd.
3-24-1, Takada, Toshima-ku, Tokyo, 170-8633
TEL: 03-3985-1111 / FAX: 03-3985-0650

1.2 Project Manager

Takashi Watanabe
General Manager, Clinical Research, Taisho Pharmaceutical Co., Ltd.

<Primary responsibilities>

(1) Assume responsibility for the overall management and control of the study.
(2) Survey and select study sites and principal investigators.
(3) Collect and evaluate continuously information regarding the quality, efficacy and safety of the investigational products and other requirements for the proper conduct of the study. Notify such information to all principal investigators and study sites participating in the study in a timely manner.
(4) Produce protocol, sample CRF and Investigator's Brochure. Revise these documents, if necessary, when important information becomes available that may be relevant to the quality, efficacy and safety of the investigational products or requirements for the proper conduct of the study. Review the Investigator's Brochure at least once yearly and revise it if necessary.
(5) Notify the principal investigator, the head of the study site and the regulatory authority of an occurrence of serious and unexpected adverse drug reaction in a timely manner.
(6) Assist the creation of informed consent form and other written information for subjects. Notify the principal investigator of an occurrence of serious and unexpected adverse reaction and new information that may affect the subject's willingness to continue participating in the study, and cooperate with the principal investigator when revising the informed consent form and other written information for subjects.
(7) Assign monitors for each study site.
(8) Request the study conduct to the heads of the study sites.
(9) Provide and retrieve investigational products that were manufactured in accordance with GMP.
(10) Produce clinical study report of the study.
(11) Compensate subject's health injury.
(12) Retain study-related documents or records.

(13) Provide cooperation to the study audits.

(14) Delegate a part of the development operation to Contract Research Organizations, as required.

1.3 Medical Expert

Yuji Kumagai
Professor, Department of Pharmacology, Kitasato University School of Medicine
Director, Clinical Trial Center, Kitasato University East Hospital
2-1-1, Asamizodai, Minami-ku, Sagamihara-shi, Kanagawa, 252-0380
TEL: 042-748-9111 / FAX: 042-741-1743

<Primary responsibilities>

(1) Make overall medical decisions and provide medical guidance and advice.

(2) Make actions to safety information.

(3) Provide advice on statistical analysis and clinical study report of the study.

(4) Provide advice on study results.

(5) Provide advice on clinical pharmacology issues.

1.4 Monitors

Clinical Research, Taisho Pharmaceutical Co., Ltd.
3-24-1, Takada, Toshima-ku, Tokyo, 170-8633
TEL: 03-3985-1306 / FAX: 03-3985-0650
Emergency Contact Center on out-of-hours
Clinical Research, Taisho Pharmaceutical Co., Ltd.
TEL: 0120-119-781

Responsible monitor: Koji Ishihara

Monitors: Iwao Kitajima, Yoichi Kiuchi, Nobuyuki Hiraga, Takasuke Nogi, Mai Muramatsu, Hitomi Nakanou, Keisuke Morikawa, Mami Ezumi, Koichi Onishi, Junya Hamanaka, Soichi Sakai, Ikuko Yataba, Nobuhiro Oba, Yoshiki Kasai, Mayumi Shibasaki, Yuri Sato, Takako Oba, Toshio Takei, Akie Yamaguchi, Rumiko Matsumoto, Takenori Sasaki, Tomohisa Shoda, Toru Bito, Morito Takahashi, Michito Ubukata, Atushi Yoshii, Hiroyuki Tamaki, Megumi Nakanishi, Koji Morikawa, Hiroki Takano, Takayuki Ugai, Daisuke Kusuhashi, Rina Matsura, Takeshi Ono, Hisae Imazeki, Yasuyuki Murase, Naofumi Arakawa, Hirohisa Omiya, Shinsuke Wada, Hirotaka Watase, Masahiro Tagawa, Ayako Yamazaki, Goro Okada, Makoto Kamiya, Noritsugu Hosoki, Hiroshi Sampei, Yusuke Kubo, Masaki Hashimoto, Haruka Kakiuchi, Yohei Shinfuku, Aya Sagara, Yukikazu Kamada, Kenji Tomatsu, Maiko Suzuki, Azusa Ubukata, Yumiko Imadera, Chie Hashimoto, Airi Takano, Chihiro Kawasaki, Takuya Hirose, Tomoyuki

Inoue, Suguru Matsuo, Ryuji Kuroishi, Satoshi Yamane, Hiromasa Sugimoto, Yuta Hasegawa, Hitomi Iemura, Hiromichi Hayashi, Yuko Horie, Shouta Tokuyamka, Kumiko Sugio, Atsushi Furuya, Kazuo Noguchi, Tomohiro Omura, Shuichi Terasaka, Yoshiki Ban, Takeshi Tani, Hideo Umeuchi, Kaori Nakamura, Masaaki Isobe, Kunika Kikumori, Mika Yamazaki, Naoki Tachibana

<Primary responsibilities>

Perform study monitoring in accordance with GCP and the sponsor's standard operating procedures.

1.5 Person Responsible for Quality Control System

Atsuko Kawano
Clinical Research, Taisho Pharmaceutical Co., Ltd.
<Primary responsibilities>

Implement quality control of the study in accordance with the sponsor's standard operating procedures.

1.6 Person Responsible for Data Management

Takayuki Magara
Clinical Research, Taisho Pharmaceutical Co., Ltd.
<Primary responsibilities>

Implement management of study data in accordance with the sponsor's standard operating procedures.

1.7 Person Responsible for Statistical Analysis

Nobuo Mizui
Biostatistics, Taisho Pharmaceutical Co., Ltd.
<Primary responsibilities>

Perform statistical analysis in accordance with the sponsor's standard operating procedures.

1.8 Auditors

Responsible auditor: Hideki Watanabe
Auditors: Kiyoshi Nakazawa, Shigenobu Sintake, Yuichi Hatada, Yoshikuni Nakamura, Nobuo Umeki, Yasuko Katsumata, Yoshinobu Yamai, Saori Ono, Seiichi Imagawa, Shizuho Noda
Study Audit, Taisho Pharmaceutical Co., Ltd.
TEL: 03-3985-1047 / FAX: 03-3590-2080
<Primary responsibilities>

Perform audits in accordance with GCP and the sponsor's standard operating procedures.

1.9 Study Drug Manager

Akiko Takahashi

Clinical Research, Taisho Pharmaceutical Co., Ltd.

<Primary responsibilities>

Implement management of the study drugs in accordance with the sponsor's standard operating procedures.

2 Study Site and Principal Investigator

Bio-Iatric Center, Kitasato University Research Center for Clinical Pharmacology
5-9-1, Shirokane, Minato-ku, Tokyo, 108-8642
TEL: 03-5791-6350 / FAX: 03-3440-5469

Principal Investigator: Tomoko Hasunuma, Director

<Primary responsibilities>

(1) Conduct the study in accordance with the protocol and GCP.
(2) Assume responsibility for overall medical decisions relating to the study.
(3) When a part of important study-related activities are delegated to a subinvestigator and/or a clinical research coordinator, prepare a list of activities and persons to be delegated, and obtain approval from the head of the study site for their designation in advance.
(4) Confirm whether or not subjects are receiving medical treatment by other primary physicians, and when applicable, inform the physician of the subject's participation in the study with the subject's approval and capture information on the medications and treatments that the subject is currently receiving.
(5) Create an informed consent form and other written information to obtain consent from subjects to participate in the study with the sponsor's assistance and revise them where necessary.
(6) Review the contractual agreement for the study and sign/seal the original or a duplicate thereof.
(7) Provide the subinvestigator and clinical research coordinator with sufficient information including protocol and guide and supervise them.
(8) Record all actions deviated from the protocol. As to protocol deviations to eliminate an immediate hazard to study subjects or for other medically compelling reasons, provide immediately the sponsor and the head of the study site with a document explaining the reason.
(9) Secure the safety of subjects by providing appropriate medical treatments for adverse events where necessary.
(10) Report immediately to the head of the study site and the sponsor in writing for all emergency incidences such as serious adverse events.

(11) Produce accurate and complete CRFs, sign/seal them and submit them to the sponsor.
(12) Review and check the CRFs produced by the subinvestigator.
(13) Discuss with the sponsor when the protocol or sample CRF needs to be revised.
(14) Provide cooperation for direct access by monitoring and audits.
(15) Retain study-related documents or records.

3 Random Assignment Manager

Yasuhito Imai
Bio-Iatric Center, Kitasato University Research Center for Clinical Pharmacology
5-9-1, Shirokane, Minato-ku, Tokyo, 108-8642
TEL: 03-5791-6350 / FAX: 03-3440-5469

<Primary responsibilities>

(1) Confirm the indistinguishability of study drugs, document the procedure and retain the record.
(2) Assign treatment sequences and study treatments. Create, retain and unblind the randomization codes of treatment sequences and study treatments.
(3) Dispense study drugs. Create and retain records for dispensing.
(4) Confirm that the blinding of the study was maintained until unblinding.
(5) Create and retain emergency keys.

4 ECG-Related Facilities

4.1 Central ECG Analysis Facility

Quintiles Cardiac Safety Services
Responsible person: Riddhi Pimputkar
Quintiles Data Processing Centre, 502-A Leela Business Park, M.V.Road, Andheri (East), Mumbai 400059. India
TEL: + 91-22-6695-0150 / FAX: + 91-22-6695-0159

<Primary responsibilities>

(1) Read the ECG data for QT/QTc analysis that are electronically sent by the study site.
(2) Analyze the ECG data based on the predetermined analysis plan.
(3) Provide the analysis report to the sponsor.

4.2 Help Desk for 12-Lead Digital Surface ECG Monitor

Quintiles Transnational Japan K.K.

Responsible person: Susumu Sakata
Forefront Tower, 3-12-1, Kachidoki, Chuo-ku, Tokyo, 104-0054
TEL: 03-3531-9581 / FAX: 03-3531-9223

<Primary responsibilities>

Provide the study site with assistance for inquiries on the 12-lead digital surface ECG monitor.

5 Person Responsible for Drug Assay

Shigeji Jingu
Pharmacokinetics, Research Center for Safety and Kinetics, Taisho Pharmaceutical Co., Ltd.
1-403, Yoshinocho, Kita-ku, Saitama-shi, Saitama, 331-9530
TEL: 048-669-3036 / FAX: 048-652-7254

<Primary responsibilities>

(1) Request the responsible laboratory to measure the drug concentrations for anticipated therapeutic dose and supratherapeutic dose treatments, and receive the assay report.
(2) Arrange the drug assay for Avelox treatment at the responsible laboratory and receive the assay report.
(3) Provide the assay reports to the product manager.

6 Drug Assay Laboratories

6.1 Assay Laboratory for Anticipated Therapeutic Dose and Supratherapeutic Dose Groups

Nishiwaki Laboratory, JCL Bioassay Corporation
Responsible person: Fumi Sekiguchi
17-18, Nakahata-cho, Nishiwaki-shi, Hyogo, 677-0032
TEL: 0795-23-5725 / FAX: 0795-23-5793

<Primary responsibilities>

Measure the plasma concentrations of unchanged TS-071 and the metabolites (M-2 and M-17) for the specimens (anticipated therapeutic dose and supratherapeutic dose groups) that are sent from the study site, and provide the assay report to the drug assay consigner (Pharmacokinetics, Research Center for Safety and Kinetics, Taisho Pharmaceutical Co., Ltd.)

6.2 Assay Laboratory for Avelox Group

Pharmacokinetics, Research Center for Safety and Kinetics, Taisho Pharmaceutical Co., Ltd.
Responsible person: Konosuke Kinoshita

1-403, Yoshinocho, Kita-ku, Saitama-shi, Saitama, 331-9530
TEL: 048-669-3036 / FAX: 048-652-7254

<Primary responsibilities>

Measure the plasma drug concentrations for the specimens (Avelox group) that are sent from the study site, and provide the assay report to the person responsible for drug assay.

<Appendix 2>
Schedule of Study Procedures

Protocol No.: TS071-02-11
Version No.: Ver.2
Date of preparation: June 23, 2011

Event	Informed consent	Screening examination	Day -1 Admission	Day 1 Day of study drug administration											Day 2			Day 3 Discharge	Post-treatment examination *5	Early termination
Time (hour) after study drug administration				Pre	0	0.5	1	2	3	4	6	8		12	24			48		
Informed consent	X																			
Demographics		X	(X)	(X)																
Hospitalization (4 days & 3 nights)			←															→		
Study drug administration					X															
Meal *1			Supper *2							Lunch			Supper		Breakfast	Lunch	Supper	Breakfast		
Physical examination		X	←															→	X	X
Height, weight		X																		
Body temperature, blood pressure, pulse rate		X		X		X	X	X	X	X					X			X	X	X
12-lead ECG for QT/QTc analysis				X		X	X	X	X	X	X	X			X					
12-lead ECG for safety analysis		X *3		X *3		X	X	X	X	X	X	X		X	X			X	X	X
Blood collection for pharmacokinetics *4				X		X	X	X	X	X	X	X		X	X			X		X*6
Clinical laboratory tests *4: Hematology		X	(X)	X											X				X	X
Clinical laboratory tests *4: Blood chemistry		X	(X)	X											X				X	X
Clinical laboratory tests *4: Urinalysis		X	(X)	X											X				X	X
Immunology tests		X																		
Urine drug screen		X																		
Pregnancy status (female only)		X		X															X	X
Adverse event monitoring					←													→	X	X

The schedule from Day -1 to Day 3 will be consistent for each period. The examination/observation displayed as "(X)" will be conducted in Period 1 only.

*1: If meal time coincides with examination or blood collection, meal will be provided after the examination or blood collection.
Contents of meals during hospitalization (from supper on Day -1 through breakfast at 48 hr post-dose) will be consistent for the same subject from Period 1 through Period 4.

*2: The supper will be provided ≥ 10 hours prior to study drug administration.

*3: Exclusion criteria (5) and (6) will be checked at screening and prior to study drug administration in Period 1.

*4: If blood collection coincides with 12-lead ECG recording, blood will be collected after 12-lead ECG.

*5: Post-treatment examination will be conducted with an interval of≥ 7 days after the final study drug administration (on Day 8 or thereafter when Day 1 is the day of study drug administration in Period 4).

*6: These will be conducted only when the study was terminated between study drug administration and 48 hours post-dose in each period.
